# Phenotypic and genomic comparison of *Photorhabdus luminescens* subsp. *laumondii* TT01 and a widely used rifampicin-resistant *Photorhabdus luminescens* laboratory strain

**DOI:** 10.1186/s12864-018-5121-z

**Published:** 2018-11-29

**Authors:** Maria-Antonia Zamora-Lagos, Simone Eckstein, Angela Langer, Athanasios Gazanis, Friedhelm Pfeiffer, Bianca Habermann, Ralf Heermann

**Affiliations:** 1 Computational Biology Group, Max-Planck-Institute of Biochemistry, Am Klopferspitz 18, 82152 Martinsried, Germany; 2 Biozentrum, Bereich Mikrobiologie, Ludwig-Maximilians-Universität München, Großhaderner Str. 2-4, 82152 Martinsried, Germany; 3 CNRS UMR 7288, Computational Biology Group, Developmental Biology Institute of Marseille (IBDM), Aix Marseille Université, 13009 Marseille, France

## Abstract

**Background:**

*Photorhabdus luminescens* is an enteric bacterium, which lives in mutualistic association with soil nematodes and is highly pathogenic for a broad spectrum of insects. A complete genome sequence for the type strain *P. luminescens* subsp. *laumondii* TT01, which was originally isolated in Trinidad and Tobago, has been described earlier. Subsequently, a rifampicin resistant *P. luminescens* strain has been generated with superior possibilities for experimental characterization. This strain, which is widely used in research, was described as a spontaneous rifampicin resistant mutant of TT01 and is known as TT01-Rif^R^.

**Results:**

Unexpectedly, upon phenotypic comparison between the rifampicin resistant strain and its presumed parent TT01, major differences were found with respect to bioluminescence, pigmentation, biofilm formation, haemolysis as well as growth. Therefore, we renamed the strain TT01-Rif^R^ to DJC. To unravel the genomic basis of the observed differences, we generated a complete genome sequence for strain DJC using the PacBio long read technology. As strain DJC was supposed to be a spontaneous mutant, only few sequence differences were expected. In order to distinguish these from potential sequencing errors in the published TT01 genome, we re-sequenced a derivative of strain TT01 in parallel, also using the PacBio technology. The two TT01 genomes differed at only 30 positions. In contrast, the genome of strain DJC varied extensively from TT01, showing 13,000 point mutations, 330 frameshifts, and 220 strain-specific regions with a total length of more than 300 kb in each of the compared genomes.

**Conclusions:**

According to the major phenotypic and genotypic differences, the rifampicin resistant *P. luminescens* strain, now named strain DJC, has to be considered as an independent isolate rather than a derivative of strain TT01. Strains TT01 and DJC both belong to *P. luminescens* subsp. *laumondii*.

**Electronic supplementary material:**

The online version of this article (10.1186/s12864-018-5121-z) contains supplementary material, which is available to authorized users.

## Background

*Photorhabdus spp.* are pathogenic enteric bacteria that maintain a mutualistic interaction with heterorhabditid nematodes and can infect a wide variety of insect species. To date, three *Photorhabdus* species are known: *P. luminescens*, *P. temperata*, and *P. asymbiotica* [1]. Whereas the first two species are highly pathogenic toward insects, *P. asymbiotica* is additionally associated with severe soft-tissue and systemic infections in humans, and is considered as an emerging threat [2]. Commonly, the bacteria colonize the gut of the infective juvenile stage of *Heterorhabditis spp.* nematodes. Upon entering insect larvae, the nematodes inject the bacteria by regurgitation into the insect’s hemocoel. Once inside the insect, the bacteria replicate rapidly and quickly establish a lethal septicaemia in the host by production of a broad range of different toxins that kill the insect within 48 h. Bioconversion of the insect’s body by *Photorhabdus spp.* produces a rich food source for the bacteria as well as for the nematodes. Nematode reproduction is supported by the bacteria, probably by providing essential nutrients that are required for efficient nematode proliferation [3]. Furthermore, the bacteria produce several secondary metabolites like antibiotics to defend the insect cadaver from invasion by other microorganisms. *P. luminescens* glows because of bacterial luciferase production. When the insect cadaver is depleted, the nematodes and bacteria re-associate and emerge from the carcass in search for a new insect host (see [﻿4﻿, ﻿5] for review).

*P. luminescens* subsp. *laumondii* strain TT01 (DSM 15139) was originally isolated from *Heterorhabditis bacteriophora* nematodes in Trinidad and Tobago [6]. Since strain TT01 was difficult to access for genetic manipulation methods, a rifampicin resistant strain was isolated by the group of David J. Clarke (University College Cork, Ireland) by growing strain TT01 in the presence of the antibiotic [7]. This strain showed enhanced suitability for genetic manipulation due to the resistance marker, and was described as a spontaneous rifampicin resistant mutant of strain TT01 (TT01-Rif^R^) [7]. In the scientific literature, authors working with either TT01-Rif^R^ or the original TT01 strain commonly refer only to TT01, making this assignment highly ambiguous [8–10].

Here we performed a phenotypic comparison between *P. luminescens* strains TT01 and TT01-Rif^R^. Since both strains differed in many phenotypic traits, we performed detailed genomic analysis, generating a finalized complete genome sequence based on the PacBio long read approach [11]. We compared the genomes of the two strains in detail and report extensive sequence differences, indicating that TT01-Rif^R^ is an independent isolate from type strain TT01. Therefore, we renamed TT01-Rif^R^ to DJC.

## Results

### Phenotypic comparison of *P. luminescens* strains TT01 and DJC

As a first step to investigate the differences between *P. luminescens* TT01 and DJC we started by comparing some of the most important phenotypes of *Photorhabdus spp.* like growth rate, pigmentation, bioluminescence, insect pathogenicity and nematode support.

**Fig. 1:**
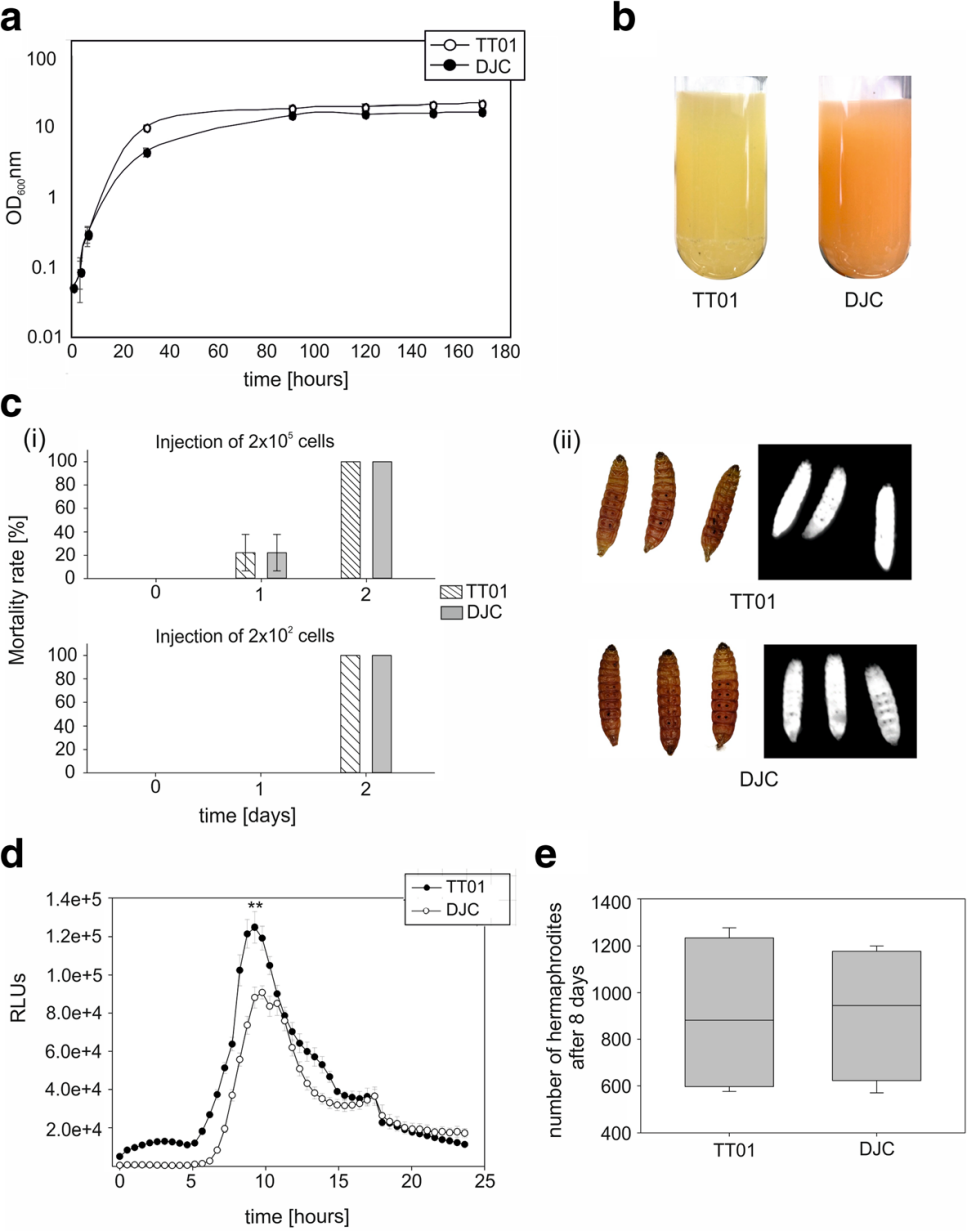
Growth, pigmentation, pathogenicity, symbiosis and luminescence of *P. luminescens* strains TT01 and DJC. **a** Growth curve of *P. luminescens* TT01 and DJC cultivated in LB broth at 30 °C for 7 days. **b** Pigmentation of a liquid culture of *P. luminescens* strain TT01 and DJC after 48 h of growth in LB broth at 30 °C. **c** Insect pathogenicity. Mortality rate of *Galleria mellonella* larvae after injection of 200,000 or 200 cells, respectively of *P. luminescens* strain TT01 or DJC (i). Pigmentation (left panel) and bioluminescence (right panel) of dead larvae 48 h after being infected by either *P. luminescens* strain TT01 or DJC (ii). **d** Quantification of bioluminescence of *P. luminescens* strain TT01 and DJC cultivated in LB broth. RLUs = Relative Light Units. **e** Symbiosis assays. Number of *Heterorhabditis bacteriophora* hermaphrodites 8 days after addition of nematodes to the respective *P. luminescens* strain as read-out for the ability to support nematode development. The asterisks (**) indicate statistically significant differences with a *p*-value smaller than 0.001. Error bars represent standard error of three independently performed experiments.

*Growth behaviour. P. luminescens* strains TT01 and DJC showed differences in growth behaviour. The growth rate in the exponential growth phase was higher for strain TT01 (μ = 0.39/h) compared to DJC (μ = 0.16/h). Furthermore, in LB broth strain TT01 (OD_600_ = 21) reached higher cell densities compared to strain DJC (OD_600_ = 16) in the stationary growth phase (*t* > 90 h) (Fig. 1a). The maximal cell density remained constant over a long period (up to 170 h) and no cell lysis was observed neither for strain TT01 nor strain DJC.

*Pigmentation.* Pigment production of both cultures was different after 48 h of cultivation. Whereas the medium containing strain TT01 became dark yellow, the one inoculated with strain DJC turned orange, revealing that both *P. luminescens* strains have differences in secondary metabolite production and/or the regulation of the corresponding genes (Fig. 1b).

*Pathogenicity and bioluminescence.* We next analyzed pathogenicity against *Galleria mellonella* wax moth larvae of both *P. luminescens* strains. For that purpose, *G. mellonella* larvae were infected with either 200 or 200,000 cells, respectively, of *P. luminescens* strain TT01 or DJC. However, we could not observe major differences in pathogenicity between the two strains: 100% of the larvae died within 48 h after infection either with strain TT01 or DJC, respectively. Approximately 1/3 of the larvae even died after 24 h for both strains at the higher bacterial load (Fig. 1c). Furthermore, *G. mellonella* larvae killed by either TT01 or DJC both turned red due to anthraquinone production and were both positive for bioluminescence (Fig. 1c). Additionally, light production of populations of both strains was quantified in liquid culture. Here we observed that bioluminescence of *P. luminescens* strain TT01 was significantly higher compared to strain DJC (*p*-value < 0.001), especially at the time point of growth when the cells entered the stationary growth phase (Fig. 1d).

*Nematode symbiosis*. To investigate the symbiotic capacity of both *P. luminescens* strains, we tested whether the bacteria were able to support nematode development. For that purpose, infective juveniles (IJs) of *Heterorhabditis bacteriophora* were added to lipid agar plates containing either *P. luminescens* strain TT01 or DJC, respectively. After 8 days of incubation, the number of hermaphrodites that developed from the IJs were counted. No significant differences between *P. luminescens* strain TT01 and DJC were observed (Fig. 1e).

**Fig. 2:**
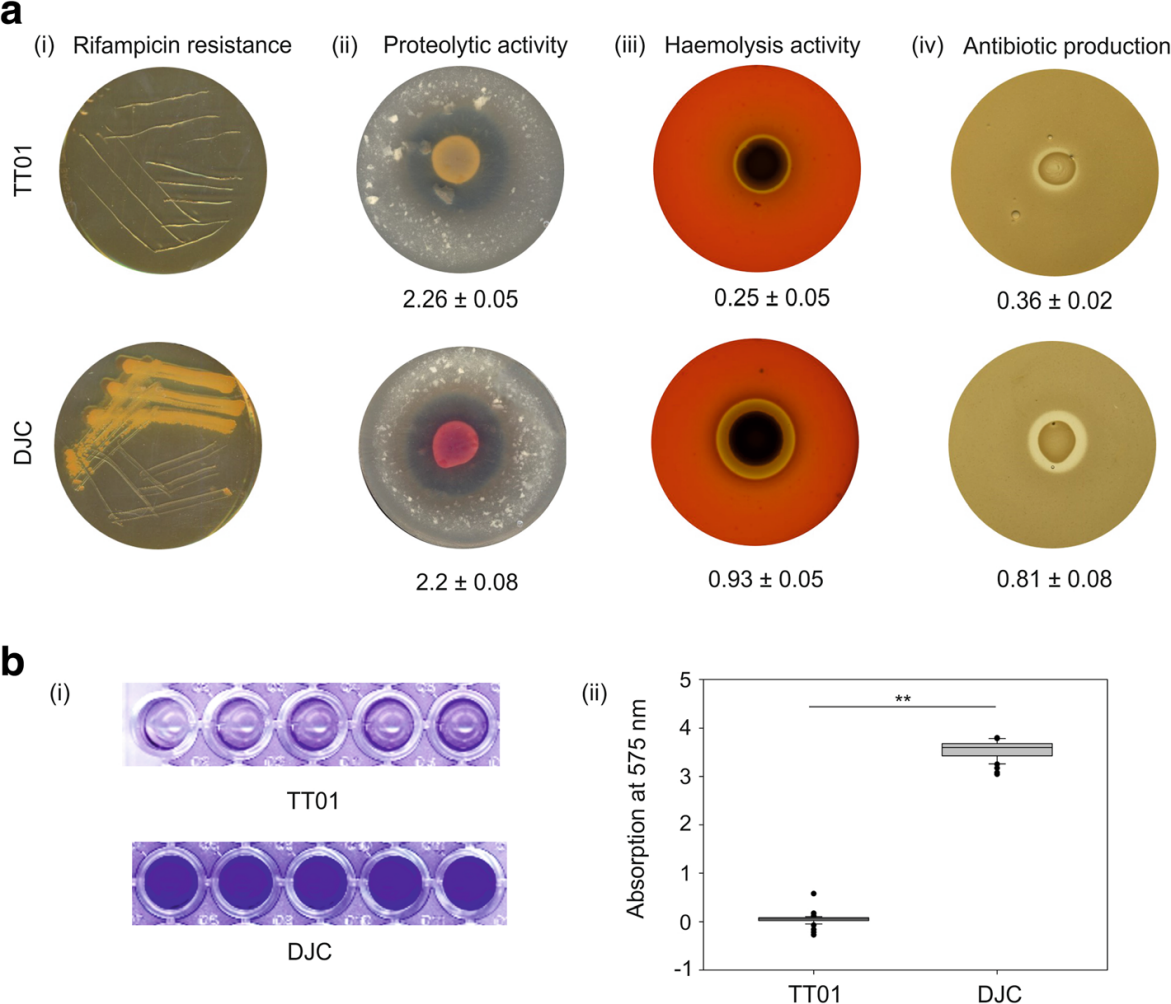
Rifampicin resistance, proteolytic and haemolytic activity, production of antibiotics and biofilm formation of *P. luminescens* strains TT01 and DJC. **a** Growth and extracellular activities of *P. luminescens* strain TT01 (upper panel) and strain DJC (lower panel). (i) Resistance towards rifampicin (50 μg/ml) after 48 h of incubation at 30 °C. (ii) Proteolytic activity on Ca-caseinate plates after 48 h of incubation at 30 °C. (iii) Secreted haemolytic activity of *P. luminescens* TT01 and DJC on sheep red blood agar plates after 4 days of incubation at 30 °C. (iv) Antibiotic effect on *B. subtilis* agar plates after 48 h of incubation at 30 °C. **b** Biofilm formation. Crystal violet staining of *P. luminescens* strain TT01 and DJC grown in LB broth and cultivated for 72 h under gentle shaking (150 rpm) at 30 °C. The planktonic cells were removed and the sessile cells, i.e. biofilm, was stained violet. The stained plates are shown on the left panel (i), whereby the quantification of the staining is shown right (ii). The asterisks (**) indicate statistically significant differences with a *p*-value smaller than 0.001. Error bars and values represent standard error of three independently performed experiments

*Rifampicin resistance*. Strains DJC and TT01 were tested for rifampicin resistance, and only strain DJC was found to be resistant (Fig. 2a). Furthermore, we tested both strains for their ability to produce exoproteinases, their ability to perform haemolysis and for antibiotic production (Fig. 2a). To compare proteolytic activity, we spotted *P. luminescens* strain TT01 and DJC, respectively, on Ca-caseinate agar plates. Both strains showed comparable protein degradation (Fig. 2a). Furthermore, we plated both strains on sheep blood agar plates and LB agar plates to investigate haemolysis and antibiotic production, respectively. Surprisingly, *P. luminescens* DJC showed a significantly higher haemolytic activity (*p*-value < 0.001) as well as antibiotic production (*p*-value < 0.05) compared to strain TT01 (Fig. 2a).

*Biofilm formation*. Finally, we analysed both strains for their ability to form biofilms. Both strains were incubated under gentle movement in cavities of 96 well plates to allow them to attach to the surface, before the medium was gently removed. The remaining cells that organized in a biofilm were re-suspended and quantified by crystal violet staining. Remarkably, strain DJC showed a significantly higher ability for biofilm production (*p*-value < 0.001) compared to TT01 (Fig. 2b). Summarizing, we found that strain DJC not only differs from strain TT01 in resistance against rifampicin, but also in many other phenotypes that are important for the *P. luminescens* life cycle, such as bioluminescence, haemolysis, antibiotic production, and biofilm formation, revealing that both strains are more different from each other than initially thought. To investigate these differences further, we decided to compare the two *P. luminescens* strains at genome level.

### Genome sequencing and assembly for *P. luminescens* strains TT01 and DJC

The genomes of *P. luminescens* strain DJC and of a variant of strain TT01 were sequenced using the long read PacBio technology with at least 180-fold coverage. This allowed us to assemble the sequences in one step into a single contig representing the final complete circular genome with high sequence reliability. The reconstructed TT01 wild-type sequence was used for all subsequent analyses (see Methods section). Further on, we refer to this genome sequence as TT01m. The overall characteristics of the genomes are shown in Table 1.

**Table 1:** General characteristics of the sequenced *P. luminescens* genomes

	*P. luminescens* DJC	*P. luminescens* TT01m	*P. luminescens* TT01
Reference	This paper	This paper	[6]
Accession	CP024900	CP024901	BX470251 (refseq:NC_005126)
Length (bp)	5,536,539	5,687,677	5,688,987
Protein-coding genes	4841	4943	4839
Pseudogenes	329	351	157
Genome coverage	194-fold	182-fold	7-fold

The type of data is indicated in the 1st column. The data are shown for the newly sequenced *P. luminescens* genomes DJC and TT01m. For comparison, data are also provided for the published version of the strain TT01 genome. Data were taken from [6]. Disrupted genes (pseudogenes) may be annotated as multiple independent genes, especially if targeted by a mobile genetic element. Such genes may not have been rated to be pseudogenes in [6]

### The newly sequenced *P. luminescens* TT01m genome sequence is highly similar to the previously published TT01 genome sequence

We first attempted to estimate the divergence between the two versions of the strain TT01 genome. We only found 30 differences between the published TT01 and our newly sequenced TT01m genome (see Additional file 1: Table S1), confirming the overall high reliability for both sequencing efforts. Observed differences included point mutations, one-base indels, copy number variations, genome inversions, and two long indels.

*Coding regions affected by genomic differences between* P. luminescens *TT01m and TT01.* We found 14 protein-coding genes that are affected by the 30 differences between the genomes. In the published TT01 genome, 2 mutations are synonymous and 5 non-synonymous, 3 mutations result in aberrant termini, 2 proteins are split, with N- and C-terminal parts annotated as independent proteins and 2 proteins are affected in multiple ways. One point mutation is located in an rRNA gene. Our new genome sequence consolidates disrupted genes in the published TT01 genome, which points to a higher reliability of the sequence we have obtained (Additional file 1: Table S1).

*Copy number variations of tandem repeats.* There were 4 differences between the genomes due to tandem repeats of 8–16 bases. In some of these, two distinct repeats are tandem-repeated directly adjacent to each other. Some tandem repeats exist in many copies (up to 47); and some show copy number differences also in the *P. luminescens* DJC genome (see below).

*Large genome inversions.* We encountered two large inversions (3.4 and 5.8 kb), one of which was associated with a frameshift difference. In both cases, the inverted region is bounded by a long inverted repeat (35 and 84 bp) and is located in a prophage region.

**Fig. 3:**
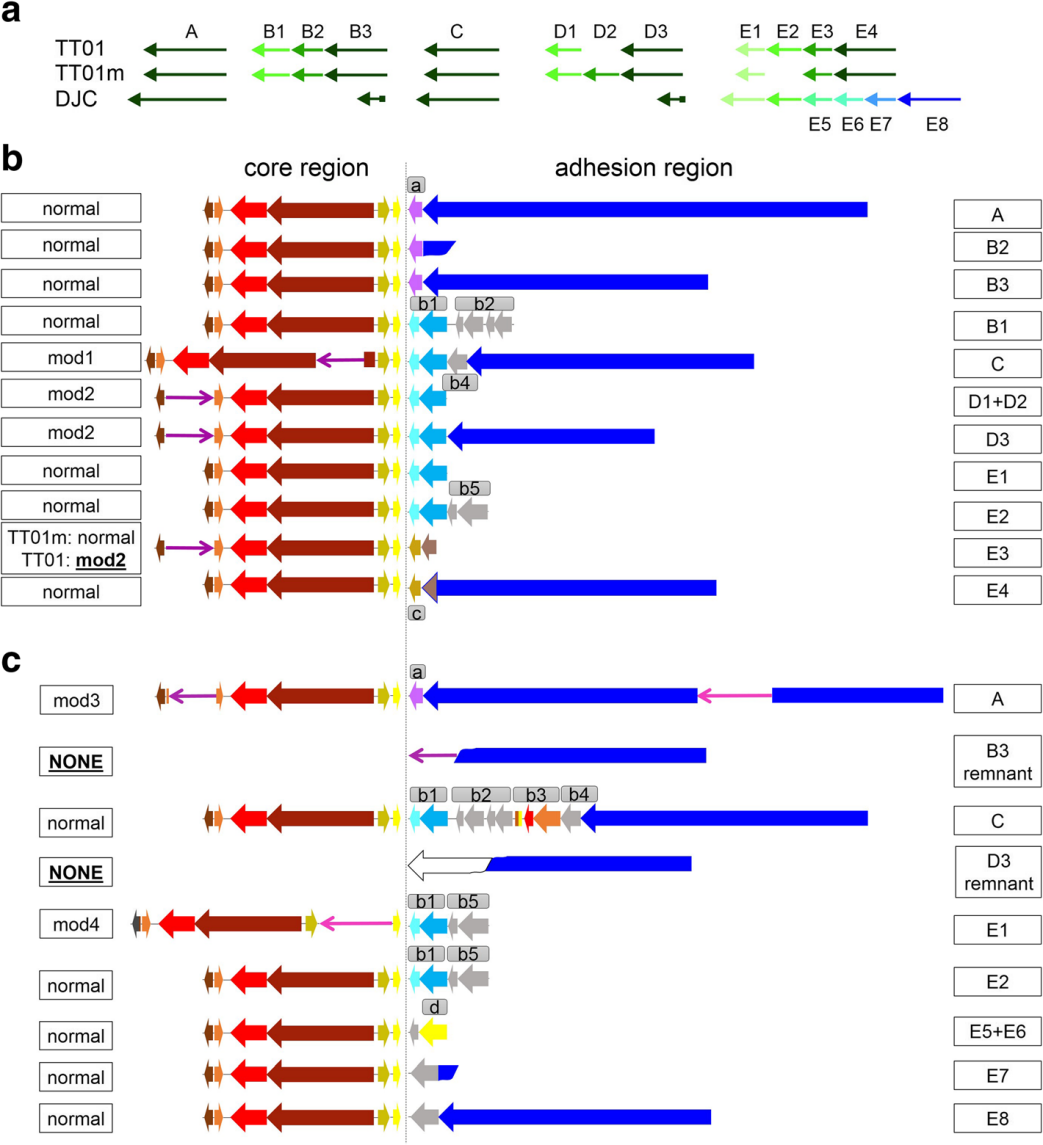
Presence of phage-related repeat PhRepA in the *P. luminescens* TT01, TT01m and DJC genome. **a** The PhRepA genes are organized in five clusters (A, B, C, D, E). Homologous clusters are drawn in similar color. (**b**, **c**) Detailed view of the prophage-related regions PhRepA in the *P. luminescens* TT01/TT01m (**b**) and DJC (**c**) genome. The different clusters are named with letters already used in the overview. The PhRepA regions can be subdivided into two parts, the “core region” (left of the vertical line) and the adhesion region (to the right). Normal = normal composition and presence of core genes; mod = modifications of the normal composition and presence of core genes. Homologous genes are displayed in similar colors. See text for details

*Large indels.* We found two large indels, one additional region in each *P. luminescens* TT01 genome version. In both cases, the observed indel is due to a highly conserved repeat, which we refer to as phage-related repeat A (PhRepA). The originally reported genome sequence for TT01 lacks the 2nd of 3 tandem copies at 4.23 Mb, while the TT01m sequence lacks the 2nd of 4 tandem copies at 4.35 Mb (Fig. 3a).

### *P. luminescens* strain DJC is an independent isolate rather than a spontaneous mutation of strain TT01

Next, we compared the sequences of the newly obtained *P. luminescens* TT01m genome with that of strain DJC. This revealed many more differences than expected given the reported genealogy, i.e. that strain DJC was a spontaneous Rif^R^ mutant [7].

We performed a detailed comparison between the two genomes based on MAFFT pairwise alignments [12] (see Methods for details). In brief, our method splits the genomes in “matching segments” (matchSEGs), most of which have less than 1% sequence difference and in “divergent segments” (divSEGs), which are either indels or regions of higher sequence divergence. The genome switches between these two types of segments.

*Matching genome segments.* The genomes were split into a total of 225 matchSEGs. These cover the majority of both genomes, 91.5% for the *P. luminescens* TT01m genome and 94.0% for strain DJC. They have an overall sequence identity of 99.7% (Additional file 1: Table S2). The majority of the matchSEGs (178 in total) cover 5.02 Mbp and have less than 1% sequence divergence (99.8% cumulative sequence identity). The residual 47 matchSEGs cover 180 kb and have more than 1% sequence difference, with 97.8% cumulative sequence identity. These are generally shorter, but only 5 are longer than 10 kb. Overall, we detected 12,967 point mutations and 333 frameshifts in the 225 matching genome segments.

*Divergent genome segments*. The strain-specific sequences (divergent segments, divSEGs) sum up to 333,729 bp for *P. luminescens* strain DJC or 6% of the DJC genome and 484,908 bp for strain TT01m (8.5% of the genome).

DivSEGs were separated into four categories according to the following characteristics (Additional file 1: Figure S1, Additional file 1: Table S2): (a) indels: an indel is continuous in one genome and has an insertion in the other so that the extra sequence can be pinpointed to an exact position. There are 83 insertions in TT01m and 68 insertions in strain DJC. (b) approximate inserts: these are inserts which can only be positioned with an error tolerance of up to 10 bp due to unaligned bases in the other genome. We encountered 13 approximate inserts, 8 in the DJC and 5 in the TT01m genome. (c) replacements: there are 47 replacements that have dissimilar sequences in both strains, located at an equivalent position with 1-base resolution. These are either completely unrelated or homologous and may reach more than 90%, but less than 95% sequence identity. (d) copy number variations: there are 6 copy number variations of tandem repeats (7–12 bp), where the number of copies differs from 10 to 49 copies.

The majority of the inserted sequences (indels and approximate inserts) are mobile genetic elements, which are described in more detail below. The remainder of the inserted sequences and the replacement sequences frequently represent genome-internal duplications (flagged InternallyRepeated in Additional file 1: Table S2). A total of 10 long insertions in either genome TT01m or DJC are prophages. Several of the larger indels or replacements represent copies of the closely related repeat PhRepA. A small number of strain-specific sequences were found to be unrelated to the other genome, having either no or only a partial BLASTn hit. On several of these, a mobile genetic element was present as a passenger along with other sequences.

Both genomes contain 6 CRISPR arrays with two variants of the repeat (GTKCACTGCCGTACAGGCAGCTTAGAAA, whereas K can be G or T). In each of the CRISPR arrays, at least some of the spacers differ (see Additional file 1: Table S2). At the end of the 2nd array, the TT01m genome has a deletion, which truncates the *cas1* gene. Some spacers occurring in one strain match to strain-specific sequences of the other strain.

In summary, the extensive differences in the two genomes make it likely that strain DJC represents an independent isolate rather than a mutant or a derivative of strain TT01. These findings support our assignment of a new strain name (DJC) instead of the original one (TT01-Rif^R^). We used our genome alignment to look at differences in protein-coding genes, prophages, as well as mobile elements between the two strains in more detail.

*Taxonomic analyses*. We compared the sets of 16S rRNA genes between TT01m and DJC. Each genome has 7 operons. When the 16S rRNAs encoded within in the TT01m genome are compared to detect polymorphisms, there are up to 10 base differences. When comparing the TT01 and DJC genomes, the 7 rRNA operons are found at equivalent positions, so that position-correlated 16S rRNA sequences can be compared. We found that 4 are identical and 2 differ by only a single base. The 7th operon is the one with the highest number of polymorphic bases in TT01m and shows 9 base differences to the 16S rRNA sequence of strain DJC. However, the DJC sequence differs by only a single base from that of another 16S rRNA, likely an effect of sequence harmonization by genome-internal translocation. We also analysed 4 conserved genes which have been proposed as taxonomic markers (*recA*, *gyrB*, *dnaN*, *gltX*) [13]. They show up to 5 point mutations, of which up to 4 are non-silent. Strain DJC shown an ANIb value of 99.49, based on 94% of its genome. From these data it can be concluded that both strains share a common taxonomic position at the subspecies level.

### Comparison of the protein-coding genes between the *P. luminescens* genomes TT01, TT01m and DJC

#### Comparing the protein-coding genes between the two versions of the P. luminescens *strain TT01 genome*

We correlated the ORFs sets of the two versions of the *P. luminescens* TT01 genome, which reflect genome annotation inconsistencies rather than genome sequence differences, with just a few exceptions. If discrepancies pointed to a problem in the newly sequenced genome, we applied manual curation to improve the annotation. The main purpose of this comparison was to provide the community with a full mapping of the established ORF codes (plu numbers) with the ORF codes as assigned by the PGAP pipeline (PluTT01m numbers). The data, which also contain the mapped codes for the DJC strain (PluDJC numbers), are provided as Additional file 2: Table S3b, and a detailed legend is provided with a sample table as Additional file 1: Table S3a.

#### Comparing the protein-coding genes between the P. luminescens *strain TT01m and strain DJC*

We correlated the ORFs sets initially predicted by the PGAP annotation pipeline for the genomes DJC and TT01m. With all genome regions assigned into matchSEGs and divSEGs and the MAFFT alignments for each segment, we could compute positional correlations and use these data for ORF mapping (for details see Methods). For cases of perfect mapping, where both termini were assigned to equivalent positions in the two genomes and were located in the same segment, and to which identical protein names had been given, we accepted the automatic annotation. All other ORFs were subjected to manual curation.

We were interested in differences between the two strains with respect to the set of their protein-coding genes. We thus extracted strain-specific protein coding genes (Additional file 1: Table S4) and those that were disrupted in one strain (pseudogene) and regular in the other (Additional file 1: Table S5). To focus on genes of higher relevance for *P. luminescens*, various gene categories were excluded, such as transposases, ORFs on the PhRepA repeat or phage-related proteins. We also excluded strain-specific genes with a close homolog of at least 75% protein sequence identity in the other strain and disrupted strain-specific genes. In total, strain DJC encodes 155 proteins that are not encoded in the TT01m genome, while 244 proteins that are found in TT01m are not present in DJC. The majority, 104 unique to DJC and 136 unique to TT01m, were annotated as hypothetical and could not be assigned a function. Both strains have sets of unique DNA-binding, DNA-modifying, restriction and DNA-replication enzymes, transcription factors, different types of toxin-antitoxin systems, as well as a set of unique proteins containing conserved domains of unknown function (DUF). However, the strain-specific proteins cannot be directly attributed to the observed phenotypic differences.

Furthermore, there are 31 and 32 disrupted genes in DJC and TT01m, respectively, which encode full-length proteins in the other strain (Additional file 1: Table S5).

Interestingly, both strains have two homologous CRISPR/CAS systems. One of the Cas3 helicases is disrupted in DJC. Most likely, prophage targeting resulted in two fragments of Cas3. In summary, both *P. luminescens* strains differ in presence or absence of a large number of genes, the majority encoding proteins of yet unknown function.

*Investigating rifampicin resistance in the DJC genome.* Rifampicin (Rif) is an antibiotic that inhibits the bacterial transcription machinery by interacting with the β-subunit of the RNA polymerase, which is encoded by *rpoB*. Mutations in *rpoB* can lead to resistance to rifampicin [14]. We investigated the genomic locus of *rpoB* in strain DJC (Rif^R^), as rifampicin resistance is the distinguishing characteristic reported for this strain. The genome of *P. luminescens* strain DJC shows 9 point mutations compared to the TT01m genome, which are located within the *rpoB* gene. While 7 mutations are silent, 2 point mutations cause amino acid replacements H526Y and E995G in the RpoB protein. It is noteworthy to mention that the H526Y replacement is located within the rifampicin-resistance hotspot 1 described for *E. coli* [15].

### Prophages and phage-related repeat PhRepA in *P. luminescens*

Many of the large-scale divergences between the genomes of *P. luminescens* TT01, TT01m, and DJC seemed phage-related. Therefore, we performed an extensive analysis of prophages. We used PhiSpy [16, 17], as well as Prophinder from the ACLAME web server [18] (see Methods for details) to predict prophages (Additional file 1: Table S6). We found considerable differences in the predictions, even if the same method was applied to near-identical genomes. If the predictions from the two programs were overlapping, we combined them as “prophage region”.

*The majority of long indels are integrated prophages.* We encountered a total of 12 long insertions (> 10 kb), 7 in the TT01m genome (up to 79 kb) and 5 in the DJC genome (up to 35 kb). Of these, 10 were assigned to be prophages according to PhiSpy and ProPhinder. An indel with 26 kb in TT01m corresponds to PhRepA copy D. An indel with 12.7 kb in DJC is unlinked to prophages. The longest sequence in the replace category of divSEGs is a 57 kb region predicted to be a prophage in the DJC genome. An unrelated 5.7 kb sequence is at the equivalent position in the TT01m genome.

*Prophage integration in coding sequences.* We observed three cases where a prophage might have targeted a protein-coding gene. The gene fragments were located more than 25 kb apart and the intervening sequences were part of predicted prophages. Coding sequence disruption is not uncommon as revealed by the bioinformatics prediction and analysis of 36,000 prophages [17]. As mentioned above, one prophage has targeted the *cas3f* gene in strain DJC. One prophage in each of the strains seems to have integrated into a pre-existing prophage, leading to a prophage conglomerate. Such conglomerates may explain the heterogeneity of the prophage prediction results from the two programs. In strain DJC, a prophage has integrated into a holin gene, in the TT01m genome, a prophage was found integrated into a restriction methylase.

*Prophages with internal inversions.* Two prophages contain an inverted region when comparing the newly sequenced TT01m genome to the published TT01 genome. The first inversion is specific to the TT01m genome while both, the published TT01 and the strain DJC genome contain this segment in the same orientation. The second inversion occurs only in the published TT01 genome while the TT01m and DJC genomes have this segment in the same orientation. However, within the same prophage region, part of the sequence is inverted in the DJC genome, whereas both versions of *P. luminescens* TT01 contain the segment in the same orientation. An additional 0.9 kb inversion in strain DJC differs from both TT01 genomes. This region however is not predicted to be a prophage.

*The phage-related repeat A region.* One prophage covers a repeat, which is a patchwork of highly conserved but also of highly diverse sequences among the analysed strains. We have named these sequences the phage-related repeat A (PhRepA) region, since some of them are in regions assigned to be prophages (Additional file 1: Table S7). The two large indels between the two versions of TT01 represent extra copies of this repeat, one in each genome (Fig. 3a). In general, there are 10 copies present in each of the TT01 genomes. In strain DJC, there are 8 copies of which 4 correspond to those of TT01m/TT01 while the other 4 are specific for strain DJC. The copies of PhRepA in the analysed *P. luminescens* genomes TT01, TT01m and DJC are schematically drawn in Fig. 3a and listed in Additional file 1: Table S7. As it can be seen, the PhRepA repeat has a tendency to form tandem duplications. Only two elements are singlets (copies A and C in both, TT01 and DJC). The other copies occur as tandem duplicates with 2 to 6 copies within each cluster (copies B, D, and E). In those clusters, the terminal copy is complete while the other copies are truncated. Many strain differences are due to heterogeneity in these clusters of tandem duplications. Two long indels between TT01 and TT01m are copies of PhRepA. Many of the PhRepA copies differ between strains TT01 and DJC: there are six tandem copies in strain DJC but only the first two correspond to the four copies found in strain TT01 in cluster E (Fig. 3a). DJC contains only remnants of clusters B and D.

Theoretically, the observed additional copies of PhRepA could represent genome assembly errors rather than biological differences. However, we consider misassembly of the TT01m genome as unlikely. Though PhRepA elements have extremely high similarity over several kb, the PacBio long read technology was shown to efficiently cope with duplications of that size [19].

PhRepA consists of two subregions that we refer to as the “core region”, which is complete in all copies and encodes 6 genes, and the “adhesion region”, which is rather diverse between different copies of PhRepA and is affected by truncation. The overview of the “core” and “adhesion” regions present in the TT01/TT01m genomes is displayed in Fig. 3b, copies and organization of these regions in the DJC genome is shown in Fig. 3c and details are described in Additional file 1: Text S1 and listed in Additional file 1: Tables S7 and S8.

The core region codes for a central gene pair, one gene containing a DNA primase (IPR13264 and IPR034151) and the other an integrase/recombinase (IPR011010) domain. This gene pair is highly conserved among all copies of PhRepA. Adjacent to the integrase is a short gene coding for a DNA-binding protein with a Cro/C1-type HTH domain (IPR001387), which is not well conserved among PhRepA copies. Located next to the gene encoding the DNA-binding protein is a gene coding for a protein with a SymE-like toxin domain (IPR014944), which also occurs in several distinct subtypes.

The adhesion region of complete PhRepA elements, which can either be singlets or terminal copies of clusters, codes for a long protein (2135–4582 amino acids) with adhesion-related domains. These include several copies of pectin lyase fold domains (IPR012334) and of hemagglutinin repeats (IPR025157). Between this gene and the core region is a rather variable set of 1 to 7 genes. Adjacent tandem-duplicated copies of the PhRepA repeat have a tendency to share the same gene set and may contain an adhesion protein remnant as a truncated gene. All genes encoded on the different copies are schematically drawn in Fig. 3b (TT01 and TT01m) and Fig. 3c (DJC) and are described in Additional file 1: Text S1 and listed in Additional file 1: Tables S7 and S8.

### Mobile genetic elements in the *P. luminescens* TT01/TT01m and DJC genomes

We performed a detailed transposon analysis of the *P. luminescens* DJC and both TT01 genomes. According to ISFinder, there are 22 distinct transposons present in *P. luminescens* [20], some of which have been submitted in the course of this study. Some of these have a high number of copies (up to ~ 20 complete copies). We also identified a few types of MITEs.

*Transposons identified in the three* P. luminescens *genomes.* Many insertions in the indels and approximate inserts represent mobile genetic elements. They commonly include a target site duplication (TSD). The relative frequency of individual transposon classes is shown in Additional file 1: Figure S2.

The transposons with the highest mobility are related to IS630. These belong to the IS630/Tc1/mariner superfamily which is found in both, prokaryotes and eukaryotes [21–23]. Although this class of transposons has been preferentially analysed in plants, such elements have also been identified in nematodes. We categorized IS630-type elements from *P. luminescens* as CCC-type (ISPlu3, ISPlu8, ISPlu19) and as AATAA-type (ISPlu10, ISPlu16), according to characteristic sequences at or very close to the beginning of the element (Fig. 5).

*MITEs identified in the three* P. luminescens *genomes.* MITEs are mobile genetic elements, which are too short to carry a transposase gene. However, they have inverted terminal repeats related to other transposons and thus are mobilized *in trans* by the corresponding transposase [24]. During our analysis, we identified 6 new MITE types and submitted these to ISFinder.

**Fig. 4:**
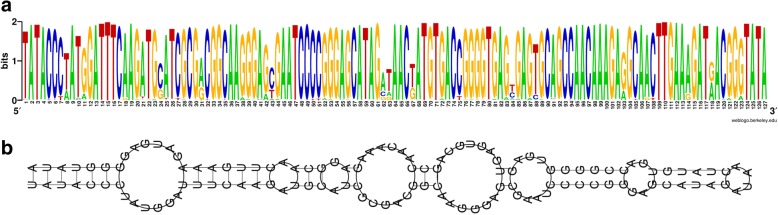
MITEPlu5 elements present in *P. luminescens* TT01 and DJC. 467 elements in the TT01 genome are complete and are 123 bp long. **a** The Weblogo of the multiple sequence alignment of the 467 MITEPlu5 element sequences from TT01, which are 123 bp long. The targeting site and target site duplication (terminal TA dinucleotide) were included in the alignment. **b** Secondary structure of the MitePlu5 consensus sequence (as read from the WebLogo) by RNAfold

**Fig. 5:**
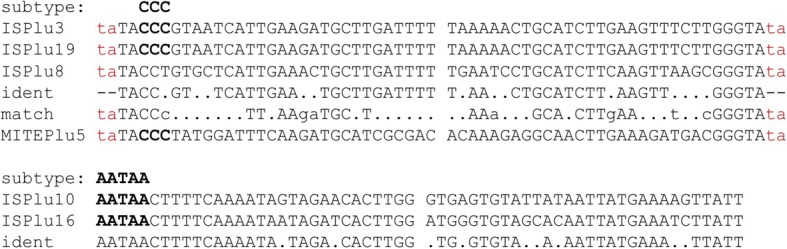
Terminal regions of IS630-type transposons in *P. luminescens* TT01 and DJC. The terminal 30 bp from IS630-type (left 5′ end, right 3′ end) including the targeting site (ta dinucleotide) and target site duplication (also ta, both in lowercase red) are shown. The MITEPlu5 sequence was read from the WebLogo (Fig. 4a). MITEPlu5 shares similarity to the CCC subtype of IS630-type transposons. Ident: bases that are identical for the transposons of the corresponding subtype are shown. Dots: differing bases. Hyphens: the targeting site and target site duplication are not part of the element. Match: bases that match between MITEPlu5 and the transposons. In the match line, uppercase: full match, lowercase: match to one of the transposons. Conservation of targeting site/target site duplication is indicated by lowercase red letters

The most frequent repeat with 552 complete copies in the TT01 genome and a typical length of 123 bp is MITEPlu5. Of the 552 complete copies, 467 have a length of 123 bp and were used to compute a sequence logo (Fig. 4a), and subsequently a consensus sequence. Given the obvious high sequence conservation, it is remarkable that only a few of these elements are truly identical to each other and that none of the copies matches exactly to the consensus sequence. This MITE seems to be highly mobile, as 47 of these elements represent indels between the TT01m and the DJC genome. A related element has been described as an ERIC sequence [25] and is reported in ISFinder as MITEEc1. MITEPlu5 shows an extremely strong secondary structure when analysed by RNAfold [26] (Fig. 4b) as also previously reported for MITEYpe1 [27]. We analysed this element in more detail (Fig. 5). We detected sequence similarities between MITEPlu5 and a subset of the IS630-type transposons with marked conservation of a CCC trinucleotide close to the terminus as found for ISPlu3, ISPlu8 and ISPlu19. For an extended description of this element see Additional file 1: Text S2.

We consider MITEPlu5 as non-coding. However, some of the copies lack stop codons in some frames. This has resulted in protein coding gene annotation by the PGAP pipeline [45]. We have retained these ORFs but have assigned the protein name “pseudocoding frame MITEPlu5” as a warning for annotation robots.

Our observations suggest that MITEs and potentially other transposable elements can lead to mis-annotations by the PGAP pipeline. Short ORFs consisting largely of MITEPlu5 and only few bases from adjacent unique genome sequence (< 100 bp) were mis-annotated to have specific protein names. The ORFs were annotated as “riboflavin synthase”, “chorismate lyase”, “addiction toxin module relE”, “SprT family protein”, “pirin family protein”. We performed BLASTx comparisons against the UniProt and NCBI nr databases to validate that the genome-derived section does not support the mis-assigned protein name. In several cases, identical mis-annotations have been made for both genomes. To avoid mis-annotation in the future, we suggest that automated annotation robots should be optimized to deal with such situations.

### Differentiation between *P. luminescens* strain TT01 and DJC via PCR

**Fig. 6:**
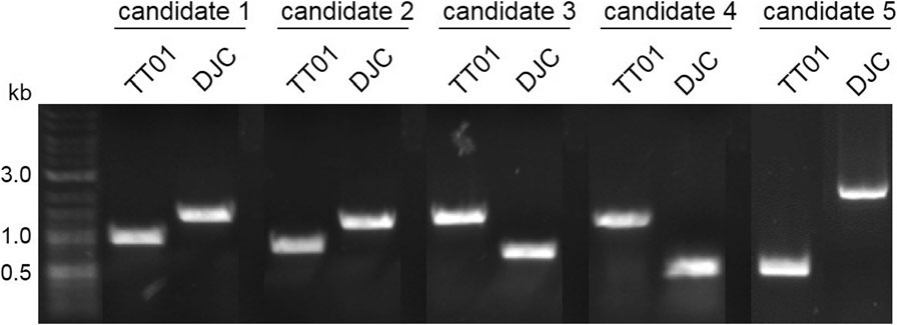
Polymerase chain reaction for *P. luminescens* strain diagnostics. In total, five candidate genetic regions were chosen, which differ in length in *P. luminescens* strain TT01 and DJC, if amplified with the same primer pair. The primer pairs chosen for the PCR reactions are listed in Additional file 1: Table S9 and the characteristics of the five candidate genes and the exact specific sizes in the DJC and TT01 genome are listed in Table 2

The knowledge that *P. luminescens* DJC and TT01 are two independent strains and the fact that scientists working with either Rif^R^ or the Rif sensitive strain refer to each of them as TT01 prompted us to design primer pairs for easy distinction between DJC and TT01 (Additional file 1: Table S9). We chose five gene regions where the same pair of primers can be used, but the PCR product length differs by at least 400 bp (Table 2; Fig. 6).

**Table 2:** Characteristics of gene regions used for PCR diagnostics to distinguish between *P. luminescens* strain TT01 and DJC

	Gene region	Putative function	TT01	DJC
Candidate 1	Parts of *plu4513–4514*	N/A	969	1443
Candidate 2	Parts of *plu2222*	Probable membrane protein	829	1217
Candidate 3	Parts of *plu2649–2651*	Hypothetical secreted protein	1264	695
Candidate 4	Parts of *plu2372–2373*	N/A	1199	487
Candidate 5	*plu1790/* insert	N/A	547	1952

The length of the amplified DNA using the primers presented in Additional file 1: Table S9 are listed in the right two columns (respective for strain TT01 and DJC)

The DJC strain was sent to the Clarke laboratory in July 2000 by the laboratory of Dr. Noel Boemare (Université de Montpellier). However, it is standard to send the *Heterorhabditis bacteriophora* nematodes carrying the bacteria rather than the isolated *Photorhabdus luminescens* strains, so that detection of phenotypic differences between TT01 type strain and another isolate is impossible. With the PCR reactions using the primers mentioned here it was demonstrated that the original frozen stock of the DJC parent strain (prepared in August 2000) produces the same profile as the Rif^R^ derivative and a distinct profile from TT01 (Dr David Clarke, data not shown). This suggests that the divergence between strain TT01 and DJC predates the arrival of this isolate in the Clarke laboratory. Although most likely being independent isolates, both strains interact specifically with *Heterorhabditis bacteriophora* nematodes.

## Discussion

We aimed to clarify the ambiguous designation of *P. luminescens* TT01. Until now *P. luminescens* strain DJC was known as a Rif^R^ derivative of strain TT01 (TT01-Rif^R^) [7]. However, we found major phenotypic as well as genomic differences between both strains. Our data in fact suggest that strain DJC is an independent *P. luminescens* isolate.

The Rif^R^ phenotype of strain DJC is an advantage in experiments where selection is required, such as genetic manipulations or strain checking. Rifampicin inhibits the bacterial transcription machinery by interacting with the *rpoB* gene. Among the two non-silent point mutations in *rpoB* in strain DJC, one (H526Y) locates within the rifampicin-resistance hotspot 1 described for *E. coli* [15]. It has been shown earlier for *P. luminescens* strain LN2 that a rifampicin resistance-causing mutation in the *rpoB* gene leading to amino acid replacement P564L developed nematocidal activity to axenic nematodes of *Heterorhabditis bacteriophora* H06 [28]. Moreover, the rifampicin resistant *P. luminescens* LN2 even supported nematode growth and development of the animals, which are normally non-compatible with the bacteria. It is assumed that at least seven putative proteins including DsbA, HlpA, RhlE, RplC, NamB, and two hypothetical proteins of unknown function were probably involved in the nematocidal activity of rifampicin resistant *P. luminescens* LN2 cells against H06 nematodes [28]. It is further assumed that altered expression of the corresponding genes is responsible for this phenotype. Here we found genomic differences concerning genes that encode putative secretion factors, regulators and genes encoding proteins of unknown function between *P. luminescens* strain DJC and TT01. However, although not checked for nematocidal activity, we found no difference in nematode symbiosis between *P. luminescens* strain DJC and TT01.

However, phenotypically both *P. luminescens* strains differed in pigmentation. The red colour of strain DJC is caused by the production of several anthraquinones [29]. The biosynthesis pathway is encoded in the *antABCDEFGHIJ* operon, which is present in both *P. luminescens* strains. The regulation of the *ant* operon has been investigated in strain DJC (earlier described as TT01-Rif^R^), and there is positive regulation of a novel type of regulator named AntJ [30]. However, a set of other proteins has been found to bind to the P_*antA*_ promoter, which might act as further repressors to tightly control anthraquinone production under different life styles of *P. luminescens* [30]. We found that both strains produced similar anthraquinone levels in insect larvae, because both turned red after infection with the bacteria. Consequently, the *ant* operon required for strain pigmentation must be regulated differently in the two strains, for instance by the presence of different inducer signals and an altered gene regulation.

Since *P. luminescens* strain DJC was initially described as a spontaneous mutant of strain TT01, the number of genome sequence differences towards strain TT01 was expected to be relatively small, and in the magnitude of a direct offspring of strain TT01, TT01m, which we have sequenced. We expected its genome sequence to be identical to the type strain except for the altered genome region, as well as a low number of anticipated genome sequencing errors. The number of sequence differences between the TT01 and TT01m genomes was only 30 and thus very low. Most differences were ambiguous with respect to distinguishing the correct and incorrect sequence version. Yet, our newly obtained *P. luminescens* TT01m genome sequence has resolved a number of obvious frameshift errors while none have been newly introduced. The encountered inversions within prophages may have occurred during strain manipulation. It is, however, also possible that there is heterogeneity within the population, which is either fixed by single cell cloning or even by random selection of one variant during genome assembly.

The observed number of differences between *P. luminescens* TT01m and DJC genomes is significantly higher, with thousands of point mutations, hundreds of frameshifts, indels, replacements, inversions and differences in transposable elements. We identified several genes and therefore proteins that are absent in either of the strains. Both strains mainly differ in the number of proteins of unknown function and those containing conserved protein domains of unknown function, which makes it difficult to correlate these with the different phenotypic traits of strains TT01 and DJC. However, as several regulatory proteins are different in both strains, also the expression of several genes that are present in both strains might be differentially regulated and mediate the different phenotypes. Furthermore, we identified several types of phage-related repeats that are present in different copy numbers in both strains. *P. luminescens* DJC lacks several clusters (B1, B2, B3-truncated, D1, D2, D3-truncated, E3, E4), but also has several repeats that are not present in strain TT01/TT01m (E5, E6, E7, E8). Since each of the repeats also contains adhesion elements, the presence or differential expression of these genes compared to strain TT01 might be involved in the higher ability of strain DJC to organize in biofilms.

It has been suggested earlier that temperate phages may play an important role in the evolution and genomic diversification of bacterial pathogens [31]. Many bacterial genomes contain a range of intact and remnant prophage elements, and important bacterial traits like bacteriocins are discussed to be phage-derived [32, 33]. Furthermore, phage-related sequences have more frequently been observed in pathogenic than in non-pathogenic bacteria, and the acquisition of prophages can also be associated with changes in pathogen virulence [34–36]. Although we have not observed major differences in pathogenicity against insects between both *P. luminescens* strains, bacterial biofilm formation is frequently known to be a virulence factor. Temperate phages have recently been observed to be involved not only in bacterial biofilm formation for the human pathogen *Pseudomonas aeruginosa*, but are also described as major drivers of host cell evolution [37].

Another interesting feature was the high number of MITEPlu5 elements that we identified in both *P. luminescens* genomes. We were astonished to find approximately 450 complete copies per genome. We were also intrigued by their similarity to eukaryotic transposable elements. Whether the MITEPlu5 elements play a role in host pathogenicity or phenotypic heterogeneity is unclear. Their similarity to eukaryotic transposable elements could point to a possible function in interacting with their hosts: in the ciliate *Tetrahymena*, an RNAi-related mechanism produces small noncoding RNAs that induce heterochromatin formation, which is followed by DNA elimination. Therefore, many transposon-related sequences are removed from the somatic macronucleus of ciliates during sexual reproduction [38]. For that reason, it is conceivable that the *P. luminescens* derived MITEPlu5 elements interfere with transposable elements in the eukaryotic host cells and thus block their life cycle. However, another possibility is that these MITEPlu5 elements or their respective RNA play a role in phenotypic heterogeneity of the bacteria and control phenotypic switching. *P. luminescens* is known to exist in two phenotypically different variants that are called primary (1°) and secondary (2°), whereby both differ in a large number of phenotypic but not genotypic traits. 2° cells develop from 1° cells during prolonged cultivation. However, both cells are comparably pathogenic towards insects, while 2° cells lack the ability to support nematode reproduction and development [39]. It has been recently discussed that, besides the activity of different transcription factors, the presence of non-coding RNAs might play a major role in the expression of 1° and 2° specific genes [8]. The protozoon *Euplotes crassus* uses transposon-like elements for precise transcriptional regulation: the Tec*1* and Tec*2* transposon-like element families are excised from the genome during a discrete time period of macronuclear development. With approximately 30,000 copies, these elements are also unusually abundant. *P. luminescens* might employ a similar mechanism during phenotypic switching. However, larger genome rearrangements have never been observed during the *P. luminescens* life cycle and phenotypic switching. Interestingly though, sequence similarity between Tec transposon-like elements and the previously described Tc*1*-IS*630* family of transposases has been observed, which includes ORFs from bacterial, nematode and insect transposons [40]. Our findings also indicate sequence-similarity of MITEPlu5 with a subtype of IS630-type transposons.

## Conclusion

Based on phenotypic and molecular comparison, we conclude that the genome sequence of *P. luminescens* strain DJC is much more divergent to TT01 than previously anticipated. With approximately 13,000 point mutations, 330 frameshifts, and 220 strain-specific regions, covering more than 300,000 bp, this strain is certainly an independent *P. luminescens* isolate. Since both *P. luminescens* strains equally interact with *H. bacteriophora* TT01 nematodes, it would appear that originally there must have been several stocks of “TT01” nematodes with the different bacterial loads. In accordance with David J. Clarke, who had originally isolated the TT01-Rif^R^ strain, the name was changed to DJC.

## Methods

### Materials

Primers used in this study are listed in Additional file 1: Table S9. PCR was performed using Q5 Polymerase or OneTaq Polymerase from New England Biolabs (Frankfurt, Germany). Restriction enzymes and T4 DNA ligase were also purchased from New England Biolabs. Genomic DNA was isolated using the Ultra-Clean Microbial DNA Isolation Kit (MoBio Laboratories, Carlsbad, USA). All other chemicals or reagents were analytical grade and obtained from commercial sources.

### Bacterial strains and growth conditions

*P. luminescens* subsp*. laumondii* TT01-Rif^R^ was obtained from the lab of David J. Clarke (University College Cork, Ireland). *P. luminescens* subsp. *laumondii* TT01 (DSM 15139) was obtained from the Deutsche Sammlung für Mikroorgansimen und Zellkulturen (DSMZ, Braunschweig, Germany). Both *P. luminescens* strains were cultivated aerobically in LB medium [1% (*w/v*) NaCl; 1% (*w/v*) tryptone; 0.5% *(w/v)* yeast extract] or CASO complex medium [5% *(w/v)* NaCl; 1.5% (*w/v*) peptone from casein; 0.5% (*w/v*) peptone from soymeal] at 30 °C. For preparation of agar plates, 1.5% (*w/v*) agar was added to the respective medium. For growth of *P. luminescens* DJC (TT01-Rif^R^), the medium was supplemented with 50 μg/ml rifampicin (Sigma Aldrich, Deisenhofen). *Bacillus subtilis* was obtained from the strain collection of Dr. Marc Bramkamp (LMU München, Germany) and cultivated in LB medium at 30 °C. Luminescence measurements were performed by cultivation of *P. luminescens* in Corning black 96-well plates with transparent bottom (Fisher Scientific, Schwerte), and optical density as well as luminescence was recorded using an Infinite-500 reader (Tecan, Salzburg).

### Caseinate bioassays

For caseinate bioassays, the bacteria were grown over night at 30 °C in LB medium. Then, an aliquot of 30 μl (OD_600_ = 1.0) was dropped onto the middle of a caseinate agar [0.5% (*w/v*) NaCl; 0.5% (*w/v*) meat extract; 0.25% (*w/v*) casein; 0.015% (*w/v*) Ca(OH)_2_; 0.005% (*w/v*) CaCl_2_; 1.35% (*w/v*) agar], and the plates were incubated for 2 d at 30 °C.

### Haemolysis bioassays

For haemolysis bioassays, the bacteria were grown over night at 30 °C in LB medium. Then, an aliquot of 30 μl (OD_600_ = 1.0) was dropped onto the middle of a haemolysis agar [0.5% (*w/v*) NaCl; 1.0% (*w/v*) meat extract; 1.0% (*w/v*) peptone; 0.5% (*v/v*) sheep blood; 1% (*w/v*) agar; pH 7.5]. The plates were incubated for 4 d at 30 °C.

### Antibiotic bioassays

For testing antibiotic activity, we used soft agar plates supplemented with *Bacillus subtilis* as test strain. For that purpose, an overnight culture of *B. subtilis* of an OD_600_ = 2–3 in 1:100 dilution was added to liquid hand-warm soft LB agar with 0.8% (*w/v*) agar. After the plates were polymerized, an aliquot of 30 μl (OD_600_ = 1.0) of the respective *P. luminescens* culture was dropped onto the middle of the agar plate and incubated for 2 d at 30 °C.

### Symbiosis bioassays

An aliquot of 50 μl of the respective *P. luminescens* overnight culture diluted to an OD_600_ of 1.0 was spread in a Z pattern onto the surface of a lipid agar plate [1% (*v*/v) corn syrup; 0.5% (*w/v*) yeast extract; 5% (*v/v*) cod liver; 2% (*w/v*) MgCl_2_ × 6 H_2_O; 2.5% (*w/v*) Difco nutrient agar (Becton Dickinson, Heidelberg)] using an inoculating loop. The plates were incubated at 30 °C for 3 days before adding 50 surface sterilized infective juvenile nematodes (IJs) to the bacterial biomass. Nematodes were surface-sterilized by washing in a solution [0.4% (*w/v*)] of hyamine (Sigma-Aldrich, Deisenhofen)]. The plates were kept at room temperature. Nematode recovery was assessed 7–8 days after addition of IJs by counting the number of hermaphrodites on the lipid agar plate.

### Pathogenicity bioassays

Fifth instar larvae of *Galleria mellonella* (reared in our lab) were incubated on ice for 10 min to reduce movements and surface sterilized in a 70% (*v/v*) ethanol bath followed by a bath of sterile water. Larvae were infected with the respective *P. luminescens* strain by injection of 10 μl cell suspension containing approximately 200 or 200,000 cells subcutaneously using a sterilized micro syringe (Hamilton 1702 RN, 25 μl), and incubated at 25 °C. Mortality rate was determined by counting dead and alive animals at several time points. At the day of larval death, luminescence was monitored using a Chemiluminescence Imager (Peqlab, Erlangen) using 5 min exposure time.

### Biofilm assays

For quantification of bacterial biofilm production, a modification of a published protocol was used [41–43]. *P. luminescens* was cultivated in LB medium over night at 30 °C. Then, the cultures were diluted in CASO medium in a volume of 125 μl per well of a 96-well polystyrene micotiter plate (Sarstedt, Nümbrecht) at a final OD_600_ of 0.5. The microtiter plate was then incubated for 72 h under gentle shaking (150 rpm) at 30 °C. Then, the liquid phase of the culture was removed by turning the plate. The planktonic cells were removed by gently submerging the plate two times in a water tub. After drying for 5 min, 125 μl of 1% (*w/v*) crystal violet (Merck, Darmstadt) was added to the wells. After 15 min incubation at room temperature, unbound crystal violet was removed by gently submerging the plate for two times in water. The plate was then dried over-night at room temperature. For quantification, 125 μl of 30% (*v/v*) acetic acid (Sigma-Aldrich, Deisenhofen) was added to each well to solubilize the crystal violet from the biofilm. After 15 min of incubation at room temperature, absorbance was quantified in a plate reader (Tecan, Salzburg) at 575 nm.

### Polymerase chain reaction (PCR)

To differentiate between *P. luminescens* strain TT01 and DJC five PCR reactions were performed amplifying DNA fragments of different length for the respective strain using identical primer pairs (Additional file 1: Table S9). First, genomic DNA from *P. luminescens* strains TT01 and DJC was isolated using the Ultra-Clean Microbial DNA Isolation Kit (MoBio Laboratories, Carlsbad, USA). PCRs were performed using OneTaq polymerase from New England Biolabs (Frankfurt, Germany) according to the manufacturer’s instructions. Oligonucleotides were purchased from Sigma-Aldrich/Merck KGaA (Darmstadt, Germany).

### Genome sequencing and assembly

Fresh cultures from *P. luminescens* subsp. *laumondii* strains DJC and a TT01 variant, in which one genome region was replaced by an antibiotic cassette, were grown in LB medium at 30 °C and harvested at exponential growth phase (OD_600_ of 2–3) by centrifugation. Genome sequencing, including DNA extraction, long-read library preparation, sequencing on a PacBio RSII sequencer, and genome assembly was performed at the Max-Planck Genome Center Cologne (http://mpgc.mpipz.mpg.de). For genome assembly, the SMRTanalysis pipeline (PacificBiosciences) was used to run HGAP (DAGCON-based hierarchical genome assembly process, RS_HGAP_assembly.2 version 2.3.0) following the steps pre-assembly, de novo assembly with the Celera assembler and final polishing with Quiver. For strain DJC, data originated from 2 SMRT cells, resulting in 300,000 raw reads. After filtering, 154,151 reads with an average length of 9770 bp (1.51 GBp total) were assembled into the chromosome, which was obtained as one contig with an average 194-fold coverage. For the TT01 mutant, data originated from 1 SMRT cell, resulting in 150,000 raw reads. After filtering, 89,346 reads with an average length of 17,496 bp (1.56 GBp total) were assembled into the chromosome, which was obtained as one contig with an average 182-fold coverage. For both genomes, the assembly resulted in a single contig with redundant termini, indicating circularization. The sequences were trimmed and the point of ring opening was shifted in order to match that of the published TT01 genome sequence [6]. A deviating region in the original assembly was converted to the TT01 wildtype sequence (positions 1,700,480–1,708,758), using Sanger sequencing data obtained for the wildtype strain. The Sanger sequencing results indicated identity to the corresponding region in the published TT01 genome.

### Genome sequence validation

In a parallel project, mutant analysis was performed by Illumina sequencing of clonal variants (AL/MA-ZL/FP/RH/BH, unpublished). The Illumina reads were mapped as described elsewhere [19]. The *P. luminescens* DJC genome was used as a reference in this comparison. Besides allowing the detection of a small number of mutations in clonal variants, this analysis also verified the correctness of the strain DJC reference genome for the bulk of the reads.

### Genome comparison

For comparison of closely related genome sequences we had developed a custom tool during the analysis of *Haloquadratum walsbyi* [44]. This tool, here referred to as “mapper”, proved useful to compare the re-sequenced TT01m genome to the originally published genome sequence of strain TT01 [6].

In brief, the mapper tool splits the input sequences into an alternate set of “runs”, defined as subsequences that are completely identical, and “connectors”, which are the divergent sequences that occur between runs. During comparison of the two TT01 genome sequence versions, nearly all of the sequences were found in runs. All encountered differences are listed in Additional file 1: Table S1. Point mutations, one-base indels and few-base differences were taken directly from the mapper output. More complex differences (inversions and long indels) were taken from BLAST analyses as the mapper tool is not capable to delineate exact coordinates.

When using the mapper tool to compare the *P. luminescens* TT01m genome to that of strain DJC, the longest region of complete sequence identity (run) was only 55 kb, indicating extensive dissimilarity. Therefore, a different strategy was applied for genome comparison, which is based on sequence alignments using MAFFT [12]. Overall, the genomes were largely co-linear but toggled between (a) “matching segments” (matchSEGs) with ca 99% sequence identity and (b) “divergent segments” (divSEGs) which were either indels or regions of increased sequence divergence.

Three passes of sequence comparison were performed. In the 1st pass, the genomes were compared in chunks of 200 kb. For each chunk, a suitable start position was selected and the subsequent sequence block of 200 kb was aligned. The beginning of the last “matching sequence” segment was selected as start position for the next chunk. Beginning at the 5′ end of both sequences, this allowed us to completely traverse both chromosomes. In the 2nd pass, individual segments of matching sequence were extracted, based on visual inspection of the aligned 200 kb chunks from the 1st pass. The segment under analysis was extended if no indel longer than 100 bp was detected or no significant increase in sequence dissimilarity was encountered. In such a case, the matchSEG was considered to have terminated. matchSEG boundaries were trimmed such that they terminated at the end with a matching base. For each matchSEG, the sequences were re-aligned with MAFFT. The resulting data were then subjected to script-based computational checking and computation of statistical data. In the 3rd pass, problems identified by the checking script were resolved. matchSEGs were split if the MAFFT alignments contained indels longer than 100 bp. matchSEGs were fused if they were separated by less than 100 bp in both genomes. All matchSEGs having more than 1% sequence divergence were visually inspected. The corresponding region could represent either a valid matchSEG with increased dissimilarity. Alternatively, it could have been misclassified as a matchSEG but actually represents a conserved but strain-specific sequence. In areas of uncertainty, we attempted to minimize matchSEGs with high divergence; at the same time, we tried not to split the genome into an unnecessarily high number of short matchSEGs.

For matchSEGs, sequence similarity statistics were computed from the MAFFT alignments by a custom script. Each position was classified to be a “match” (m), a “mismatch” (mm), a “gap open” (go) or a “gap extension” (ge) position. Gap extension positions were excluded from subsequent computations. Therefore, sequence difference is calculated as “mm + go/ m + mm + go”.

matchSEGs are separated by divergent segments (divSEGs). These were classified into categories and tagged by content as detailed in the text and in the legend to Additional file 1: Table S2. After finalization of the analysis, it was ensured that each genome position is classified exactly once, either as part of a matchSEG or part of a divSEG. All MAFFT alignments were confirmed to represent the specified genome region. We ensured that each matchSEG starts and ends with a matching base. The complete list of matchSEGs and divSEGs is provided in Additional file 1: Table S2.

“Pairwise position correlation data” were computed for the *P. luminescens* DJC and TT01m genomes. Each genome position was classified into one of three categories: (i) “mapped” to a position in the other genome; these positions are within a matchSEG and the positional correlation is computed from the MAFFT alignment; (ii) “gap”: a position in a matchSEG, is located opposite to a gap in the other genome in the MAFFT alignment; (iii) “strain-specific”; these positions are within a divSEG.

### Genome annotation

An automatic annotation was generated using the NCBI PGAP pipeline upon GenBank submission [45]. The annotation was only partially subjected to further curation (see below).

To support the annotation process, the proteome from strain TT01 was downloaded from UniProt (UP000002514, release 2017_10), as well as from GenBank (accession BX470251) [6].

### Correlation of the theoretical proteomes of the two genome sequences of *P. luminescens* TT01

Using a set of custom scripts combined with manual inspection, the (curated) theoretical proteome of the *P. luminescens* TT01m genome was compared to the published proteome of strain TT01 as extracted from GenBank (accession BX470251). Because genome sequence differences are minor (Additional file 1: Table S1), a “pairwise position correlation” could be easily computed as a tool for ORF correlation.

We attempted to correlate each protein-coding gene [hereafter referred to as open reading frame (ORF)] from the *P. luminescens* TT01m genome version to an ORF from the TT01 genome version. All ORFs, which traverse any of the sequence differences between the TT01 and TT01m genomes (Additional file 1: Table S1) were excluded from automatic analysis and were correlated manually. Automatic ORF matching was based on the detection of corresponding C-terminal positions. The protein sequences of the correlated ORFs must be identical in case of a consistent start codon assignment, given that the genomes are identical except for 30 differences. For inconsistent start codon assignments, the C-terminal fragments must be identical for the length of the shorter ORF if the assigned start codon is an ATG. If the shorter sequence has GTG or TTG assigned as a start codon, the internal Val or Leu of the longer sequence was converted to Met prior to sequence comparison. About 89% of both proteomes could be automatically mapped by this procedure. The remainder of the proteomes was subjected to manual correlation, mainly using the BLAST suite of program [46]. A significant fraction of the ORFs which cannot be automatically mapped were either (a) disrupted and hence pseudogenes or invalidly considered to be disrupted; (b) missing gene calls in the published TT01 genome; (c) not mappable due to missing gene calls by the PGAP annotation pipeline. Such ORFs were post-predicted, except for few short fragments of disrupted genes; (d) spurious ORFs: several ORFs in TT01 were rated to be spurious, i.e. ORFs which are unlikely to be protein-coding genes (for usage of this term see [47]). Such spurious ORFs are typically not predicted by the PGAP pipeline, are short, and have no or extremely few BLAST hits in the UniProt database (as analysed in January 2018). It should be noted that disrupted genes may be annotated as a single ORF in one strain, but a set of two or three ORFs in the other strain.

An exhaustive list with all correlated and non-correlated ORF codes (locus tags) is provided for the genomes from *P. luminescens* strain TT01 (plu numbers), TT01m (PluTT01m numbers) and DJC (PluDJC numbers) as Additional file 2: Table S3b; Additional file 1: Table S3a.

### Correlation of the theoretical *P. luminescens* TT01 and DJC proteomes

The theoretical proteomes predicted for the *P. luminescens* DJC and TT01m genomes by the PGAP pipeline were compared in detail, using custom PERL scripts.

Again, we attempted to correlate each protein-coding gene from one strain to an ORF from the other strain. The mapping was based on positional correlation, using the “pairwise position correlation data” (see above). We first tried to correlate ORFs by their C-terminal positions. For ORFs, which could be correlated by C-terminal position, we checked if the N-terminal position can be correlated as well. For ORFs which could not be correlated by their C-terminal position, we attempted correlation by their N-terminal position. It should be noted that this algorithm allows a correlation only if at least one of the termini is within a matchSEG (see above). When correlation was successful and both termini were within the same matchSEG, the ORF was classified as perfectly correlated. Such perfectly correlated ORFs were excluded from subsequent manual curation unless their protein names differed or they were disrupted genes according to the PGAP pipeline. All ORFs that did not show such a perfect correlation were subjected to manual curation (see below).

Manual curation triggered various annotation updates (e.g. improvement of the protein name or start codon reassignment). Also, some disrupted genes (i.e. pseudogenes) were initially annotated as regular by PGAP and vice versa. Finally, some of the annotated genes were found to be “spurious ORFs”.

For manual curation, ORFs were subjected to BLAST analyses [46]. BLASTp comparisons were made against the theoretical proteomes from the two *P. luminescens* strains DJC and TT01m, as well as the UniProt proteome of strain TT01. BLASTx comparisons were carried out against the DJC, TT01m and TT01 genomes. Protein-coding genes, which were regular in one strain but disrupted in the other (Additional file 1: Table S5) were identified and validated by BLASTx analyses. For some ORFs, positional mapping had failed but BLASTp analysis allowed to identify the correlation. The analysis allowed us to identify missing gene calls, if ORFs initially seemed strain-specific but showed strong BLASTx matches. Such ORFs were post-predicted and correlated manually. Other ORFs were validated to be strain-specific (Additional file 1: Table S4) by BLASTp and BLASTx analyses. Some ORFs predicted in only one strain were rated to be spurious when they were short, a corresponding gene would have been disrupted in the other genome, and there were no or extremely few BLAST hits in UniProt.

### Additional bioinformatics tools

As general tools, MUMMER and the BLAST suite of programs were used for genome comparisons [46]. For ORF post-prediction, we used the Translate Tool from the Expasy Server (https://www.expasy.org). We analysed the *P. luminescens* TT01 and DJC genomes for CRISPRs encoding genes using the CRISPRFinder web server (http://crispr.i2bc.paris-saclay.fr) [48]. Prophages were analysed for all three strains by PhiSpy (http://edwards.sdsu.edu/PhiSpy) [16, 17] and for the newly sequenced *P. luminescens* strains by Prophinder (http://aclame.ulb.ac.be/Tools/Prophinder) [18]. Prophages for the published sequence of TT01 were found pre-computed on the ACLAME web server [18]. Phage-related repeat PhRepA was analysed using the BLAST suite, including BLASTx comparison against the UniProt database. RNA secondary structures were predicted using the RNAfold webserver from the ViennaRNA Web Services (http://rna.tbi.univie.ac.at) [26]. For ANIb computations (based on BLASTn analyses) we used JSpeciesWS (http://jspecies.ribohost.com/jspeciesws) [49].

### Transposon analysis

Transposons were identified by BLASTn and BLASTx comparison to the ISFinder database [20, 24] by a described procedure [19]. Identified transposons were collected in an in-house database and were used for a subsequent iterative transposon analyses using BLAST. Few additional transposons were identified and submitted to ISFinder. In several cases, our analyses showed that the boundaries of the transposons in ISFinder needed to be shifted. This information was forwarded to ISFinder. In addition to canonical transposons, we identified several MITEs (Miniature Inverted-Terminal-repeat Elements), which were submitted to and accepted by ISFinder for their recently introduced MITE subsection.

### Note added in proof

*Photorhabdus luminescens* subsp. *laumondii* has been recently suggested to be renamed as *Photorhabdus laumondii* (Machado et al 2018 10.1099/ijsem.0.002820) [50].

## Additional files

Additional file 1: Text S1. Phage-related repeat PhRepA in *P. luminescens*. Text S2. **Mobile genetic elements in the** ***P. luminescens*** **TT01/TT01m and DJC genomes**. **Table S1.** Differences between the newly sequenced *P. luminescens* genome TT01m and the originally published genome TT01. **Table S2.** Genome comparison between *P. luminescens* strain DJC and TT01 (as represented by the newly sequenced genome TT01m). **Table S3a.** Mapping of gene codes between the TT01, TT01m and DJC genomes. **Table S4.** Strain-specific protein-coding genes. **Table S5.** Genes which are disrupted in only one *P. luminescens* strain. **Table S6.** Prophages as predicted by PhiSpy and Prophinder. **Table S7.** Copies of phage-related Repeat A (PhRepA) in the three *P. luminescens* strains. **Table S8.** Genes and intergenic regions on PhRepA in the element copies. **Table S9.** Oligonucleotides used for *P. luminescens* strain. **Figure S1.** Relative contribution of divergence region classes between *P. luminescens* TT01 and DJC. **Figure S2.** Relative frequency of transposons and other mobile genetic elements in indels and approximate inserts. (PDF 2620 kb)

Additional file 2: **Table S3b.** Mapping of gene codes between the TT01, TT01m and DJC genomes. (XLSX 191 kb)
