# Supplementary material for: Phenotypic and genomic comparison of Photorhabdus luminescens subsp. laumondii TT01 and a widely used rifampicin-resistant Photorhabdus luminescens laboratory strain
Source: BMC Genomics. 2018 Nov 29;19:854. doi: 10.1186/s12864-018-5121-z (PMC6267812; doi:10.1186/s12864-018-5121-z)
Supplement: Supplementary file 1 — Text S1. Phage-related repeat PhRepA in P. luminescens. Text S2. Mobile genetic elements in the P. luminescens TT01/TT01m and DJC genomes. Table S1. Differences between the newly sequenced P. luminescens genome TT01m and the originally published genome TT01. Table S2. Genome comparison between P. luminescens strain DJC and TT01 (as represented by the newly sequenced genome TT01m). Table S3a. Mapping of gene codes between the TT01, TT01m and DJC genomes. Table S4. Strain-specific protein-coding genes. Table S5. Genes which are disrupted in only one P. luminescens strain. Table S6. Prophages as predicted by PhiSpy and Prophinder. Table S7. Copies of phage-related Repeat A (PhRepA) in the three P. luminescens strains. Table S8. Genes and intergenic regions on PhRepA in the element copies. Table S9. Oligonucleotides used for P. luminescens strain. Figure S1. Relative contribution of divergence region classes between P. luminescens TT01 and DJC. Figure S2. Relative frequency of transposons and other mobile genetic elements in indels and approximate inserts. (PDF 2620 kb) [file 12864_2018_5121_MOESM1_ESM.pdf]

Phenotypic and genomic comparison of *Photorhabdus luminescens* subsp. *laumondii* TT01 and a widely used rifampicin-resistant *Photorhabdus luminescens* laboratory strain

Additional file 1

Maria-Antonia Zamora-Lagos[1*], Simone Eckstein[2*], Angela Langer[2], Athanasios Gazanis[2], Friedhelm Pfeiffer[1], Bianca Habermann[1,3#] and Ralf Heermann[2#]

[1]Computational Biology Group, Max-Planck-Institute of Biochemistry, Am Klopferspitz 18, 82152 Martinsried, Germany

[2]Ludwig-Maximilians-Universität München, Biozentrum, Bereich Mikrobiologie, Martinsried, Germany

[3] Aix Marseille Université, CNRS UMR 7288, Computational Biology Group, Developmental Biology Institute of Marseille (IBDM) UMR 7288, CNRS, Marseille 13009, France

* equal contribution; # co-corresponding authors

Corresponding authors addresses:

Dr. Bianca Habermann

Aix Marseille Université, CNRS UMR 7288, Computational Biology Group, Developmental Biology Institute of Marseille (IBDM), Marseille 13009, France

Phone: +33-4-91269236

e-mail: bianca.HABERMANN@univ-amu.fr

PD Dr. Ralf Heermann

Biozentrum, Bereich Mikrobiologie, Ludwig-Maximilians-Universität München, Großhaderner Str. 2-4, 82152 Martinsried, Germany

Phone: +49-89-2180-74506

e-mail: heermann@lmu.de

Additional file 1

Phage-related repeat PhRepA in *P. luminescens*

One prophage covers a repeat, which is a patchwork of highly conserved but also of highly diverse sequences among the analysed strains. We have named these sequences the phage-related repeat A (PhRepA) region, since some of them are in regions assigned to be prophages (Supplementary Table S7). The two large indels between the two versions of TT01 represent extra copies of this repeat, one in each genome (Figure 3a). In general, there are 10 copies present in each of the TT01 genomes. In strain DJC, there are 8 copies of which 4 correspond to those of TT01m/TT01 while the other 4 are specific for strain DJC. The copies of PhRepA in the analysed *P. luminescens* genomes TT01, TT01m and DJC are schematically drawn in Figure 3a and listed in Supplementary Table S7. As it can be seen, the PhRepA repeat has a tendency to form tandem duplications. Only two elements are singlets (copies A and C in both, TT01 and DJC). The other copies occur as tandem duplicates with 2 to 6 copies within each cluster (copies B, D, and E). In those clusters, the terminal copy is complete while the other copies are truncated. Many strain differences are due to heterogeneity in these clusters of tandem duplications. Cluster B has three tandem copies in the two versions of TT01. Cluster D has three tandem copies in the new version of TT01m while the central copy is lacking from the published TT01 genome. *P. luminescens* strain DJC contains only a remnant of clusters B and D. Cluster E contains 4 tandem copies in the published TT01 genome while the second copy is not present in the new TT01m genome. There are six tandem copies in strain DJC but only the first two correspond to those of strain TT01 (Figure 3a).

Theoretically, the observed additional copies of PhRepA could represent genome assembly errors rather than biological differences. However, we consider misassembly of the TT01m genome as unlikely. Though PhRepA elements have extremely high similarity over several kb, the PacBio long read technology was shown to efficiently cope with duplications of that size [1].

PhRepA consists of two subregions that we refer to as the "core region", which is complete in all copies and encodes 6 genes, and the "adhesion region", which is rather diverse between different copies of PhRepA and is affected by truncation. The overview of the “core” and “adhesion” regions present in the TT01/TT01m genomes is

displayed in Figure 3b, copies and organization of these regions in the DJC genome is shown in Figure 3c and details are listed in Supplementary Tables S7 and S8.

The core region codes for a central gene pair, one gene containing a DNA primase (IPR13264 and IPR034151) and the other an integrase/recombinase (IPR011010) domain. This gene pair is highly conserved among all copies of PhRepA, including a 14 bp overlap between the two genes. Such a long overlap is not uncommon for genes with a -2 bp shift. Adjacent to the integrase is a short gene coding for a DNA-binding protein with a Cro/C1-type HTH domain (IPR001387), which is also found in phage lambda repressor. Genes encoding proteins with such a domain are not well conserved among PhRepA copies but belong to 5 distinct subtypes (*plu0545*, *plu1127*, *plu3581*, *plu3715* and PTE_00007). Located next to the gene encoding the DNA-binding protein is a gene coding for a protein with a SymE-like toxin domain (IPR014944), which also occurs in several distinct subtypes. On the other side of the primase gene are the two short, conserved proteins *plu1142-like* and *plu0542*, which both lack assigned domains. The *plu1142* homologs are located in a 550 bp region, in which various ORFs have been assigned in the published TT01 genome. These ORFs are, however, self-contradictory. We selected *plu1142* as the most likely protein-coding gene in this region, although the *plu1142* homolog adjacent to *plu0542* is a missing gene call and several other copies in both strains are disrupted by an in-frame stop codon. The other annotated ORFs would have even more pronounced annotation problems if assigned to all PhRepA copies. Therefore, we consider them to be spurious.

The adhesion region of complete PhRepA elements, which can either be singlets or terminal copies of clusters, codes for a long protein (2135-4582 amino acids) with adhesion-related domains. These include several copies of pectin lyase fold domains (IPR012334) and of hemagglutinin repeats (IPR025157). Between this gene and the core region is a rather variable set of 1 to 7 genes. Adjacent tandem-duplicated copies of the PhRepA repeat have tendency to share the same gene set and may contain an adhesion protein remnant as a truncated gene. The PhRepA remnants B3 and D3 in strain DJC lack the whole adjacent sequence element except for a partial copy of the adhesion protein gene. In cluster B3, the gene is truncated by an ISPlu9 transposon. In cluster D3, a gene fusion couples the N-terminal 2135 residues of the adhesion protein in-frame to the C-terminal 883 residues of the protein preceding the PhRepA cluster. The N-terminal part of the gene in cluster D3 is highly similar to the genes of copy A and of the DJC-specific copy E8. All genes encoded on the different copies are

schematically drawn in Figure 3b (TT01 and TT01m) and Figure 3c (DJC) and are listed in Supplementary Table S7. It should be noted that four of the five clusters (A,B,D,E) include a gene encoding a secretion pathway transporter (also referred to as haemolysin activator), whereby the corresponding gene is located directly adjacent to the gene encoding the adhesion protein. The secretion transporter pathway gene is not covered by our definition of PhRepA.

Text S2: Mobile genetic elements in the *P. luminescens* TT01/TT01m and DJC genomes

We performed a detailed transposon analysis of the *P. luminescens* DJC and both TT01 genomes. According to ISFinder, there are 22 distinct transposons present in *P. luminescens* [2], some of which have been submitted in the course of this study. Some of these have a high number of copies (up to ~20 complete copies). In addition, there are many disrupted transposons. Only few of those have intact termini, but all of those have a disrupted transposase gene, making them unsuitable for ISFinder. We also identified a few types of MITEs.

Transposons identified in the three P. luminescens *genomes.* Many insertions in the indels and approximate inserts represent mobile genetic elements. They commonly include a target site duplication (TSD). The relative frequency of individual transposon classes is shown in Supplementary Figure S2. Initially, we found that ISPlu1 and ISPlu4 seemed to lack a TSD. However, we identified extra bases at the insertion points and the extended elements are bounded by a TSD. Therefore, these elements are a few bases longer than annotated by ISFinder. The extended bases are not palindromic and thus were initially not recognized as part of the inverted terminal repeats.

The transposons with the highest mobility are related to IS630. These belong to the IS630/Tc1/mariner superfamily which is found in both, prokaryotes and eukaryotes [3-5]. Although this class of transposons has been preferentially analysed in plants, such elements have also been identified in nematodes. Upon *in vitro* transposition of a bacterial element it could be shown that a sequence-specific TA dinucleotide is used for integration and that the element generates a 2 bp target site duplication (another TA dinucleotide) [6]. Defining element boundaries exclusively by sequence conservation consequently leads to some ambiguity, because the targeting site/TSD

will be included as part of the element. We categorized IS630-type elements from *P. luminescens* as CCC-type (ISPlu3, ISPlu8, ISPlu19) and as AATAA-type (ISPlu10, ISPlu16), according to characteristic sequences at or very close to the beginning of the element (Supplementary Figure S2).

MITEs identified in the three P. luminescens *genomes.* MITEs are mobile genetic elements, which are too short to carry a transposase gene. However, they have inverted terminal repeats related to other transposons and thus are mobilized *in trans* by the corresponding transposase [7]. During our analysis, we identified 6 new MITE types and submitted these to ISFinder.

The most frequent repeat with 552 complete copies in the TT01 genome and a typical length of 123 bp is MITEPlu5. Of the 552 complete copies, 467 have a length of 123 bp and were used to compute a sequence logo (Figure 4a), and subsequently a consensus sequence. Given the obvious high sequence conservation, it is remarkable that only a few of these elements are truly identical to each other and that none of the copies matches exactly to the consensus sequence. If fragments, targeted elements, and those with internal deletions are summarized, they result in a total of 660 copies (strain TT01; 649 in DJC). 47 of these elements represent indels between the TT01m and the DJC genome. A related element has been described as an ERIC sequence [8]. This element has recently been added to the ISFinder database as MITEEc1, based on our submission. MITEPlu5 shows an extremely strong secondary structure when analysed by RNAfold [9] (Figure 4b) as also previously reported for MITEYpe1 [10]. We analysed this element in more detail (Figure 5). We detected sequence similarities between MITEPlu5 and a subset of the IS630-type transposons with marked conservation of a CCC trinucleotide close to the terminus. This makes it likely that MITEPlu5 is related to the CCC subtype of IS630-type transposons, which is represented by ISPlu3, ISPlu8 and ISPlu19. A similar situation exists for MITEPlu2, which is related to the AATAA subtype of IS630-type transposons represented by ISPlu10 and ISPlu16. Of all transposons/MITEs found inserted in one of the two genomes, 49% belong to the CCC subtype of IS630-type, making this the most active element for transposition.

We consider MITEPlu5 as non-coding. However, some of the copies lack stop codons in some frames. This has resulted in protein coding gene annotation by the PGAP pipeline. We have retained these ORFs but have assigned the protein name

"pseudocoding frame MITEPlu5" as a warning for annotation robots. We also encountered several cases in which MITEPlu5 is fused in-frame with upstream or downstream genes. We consider these genes disrupted and removed the protein sequence segment translated from MITEPlu5. In very few cases the MITEPlu5 is integrated into the original stop codon, as it targets TA dinucleotides. This led to a MITEPlu5-derived C-terminal extension. To avoid annotation problems caused by such extremely well conserved extensions, we truncated the ORFs at the junction between the genomic sequence and MITEPlu5.

Our observations suggest that MITEs and potentially other transposable elements can lead to mis-annotations by the PGAP pipeline. Short ORFs consisting largely of MITEPlu5 and only few bases from adjacent unique genome sequence (<100 bp) were mis-annotated to have specific protein names. The ORFs were annotated as "riboflavin synthase", "chorismate lyase", "addiction toxin module relE", "SprT family protein", "pirin family protein". We performed BLASTx comparisons against the UniProt and NCBI nr databases to validate that the genome-derived section does not support the mis-assigned protein name. In several cases, identical mis-annotations have been made for both genomes. To avoid mis-annotation in the future, we suggest that automated annotation robots should be optimized to deal with such situations.

References

1. Pfeiffer F, Zamora-Lagos M-A, Blettinger M, Yeroslaviz A, Dahl A, Gruber S, et al. The complete and fully assembled genome sequence of *Aeromonas salmonicida* subsp. pectinolytica and its comparative analysis with other *Aeromonas* species: investigation of the mobilome in environmental and pathogenic strains. BMC Genomics. 2018;19:20.

2. Siguier P, Varani A, Perochon J, Chandler M. Exploring bacterial insertion sequences with ISfinder: objectives, uses, and future developments. Methods Mol. Biol. Totowa, NJ: Humana Press; 2012;859:91–103.

3. Tellier M, Bouuaert CC, Chalmers R. Mariner and the ITm superfamily of transposons. Microbiol Spectr. 2015;3:MDNA3–0033–2014.

4. Coy MR, Tu Z. Gambol and Tc1 are two distinct families of DD34E transposons: analysis of the *Anopheles gambiae* genome expands the diversity of the IS630-Tc1-mariner superfamily. Insect Mol. Biol. Blackwell Science Ltd; 2005;14:537–46.

5. Studer N, Frey J, Vanden Bergh P. Clustering subspecies of *Aeromonas salmonicida* using IS630 typing. BMC Microbiol. 2013;13:36.

6. Feng X, Colloms SD. In vitro transposition of ISY100, a bacterial insertion sequence belonging to the Tc1/mariner family. Mol. Microbiol. Blackwell Publishing Ltd; 2007;65:1432–43.

7. Filée J, Siguier P, Chandler M. Insertion sequence diversity in archaea. Microbiol. Mol. Biol. Rev. 2007;71:121–57.

8. Hulton CS, Higgins CF, Sharp PM. ERIC sequences: a novel family of repetitive elements in the genomes of *Escherichia coli*, *Salmonella typhimurium* and other enterobacteria. Mol. Microbiol. 1991;5:825–34.

9. Gruber AR, Lorenz R, Bernhart SH, Neuböck R, Hofacker IL. The Vienna RNA websuite. Nucleic Acids Res. 2008;36:W70–4.

10. De Gregorio E, Silvestro G, Petrillo M, Carlomagno MS, Di Nocera PP. Enterobacterial repetitive intergenic consensus sequence repeats in Yersiniae: genomic organization and functional properties. J. Bacteriol. 2005;187:7945–54.

Supplementary Table S1: Differences between the newly sequenced *P. luminescens* genome TT01m and the originally published genome TT01. A total of 30 differences was detected (nr). For each genome, the differing position/region and the sequence-specific bases (or their length) is provided. Alternatively, the sequence of a tandem repeat with its copy number in parenthesis is given. A deletion is indicated by a hyphen. In this case, the adjacent bases are specified, separated by a slash (/). The type of difference is named as "point" (point mutation), "shift" (one-base indels, eventually as part of a polynucleotide run), "diff" (for adjacent mutations and/or indel of a few bases), "indel" (all of which are longer than 1 kb and are related to the long repeat PhRepA), "inversion", or "copy-nr" (copy number differences for tandem repeats). Note that the 1st difference is an inversion (1a) having an internal "shift" difference (1b).

	***P. luminescens* TT01m**		***P. luminescens* TT01**			
nr	pos	seq/length	pos	seq/length	type	comment
1a	26555-29975	3421 bp (fwd)	29976-26555	3422 bp (rev)	inversion	bounded by a 86 bp inverted repeat; N-termini of outer ORFs within repeat; =-31--> -32-> <--33-- <--34-= (plu0031-plu0034); on prophage R01
1b	28275-28276	GG	28256-28254	GGG	shift	this one-base indel is in the intergenic region between *plu0033* and *plu0034*; on prophage R01
2	43959	T	43960	C	point	point mutation in *plu0049* leading to amino acid replacement G177D
3	282721	A	282722	C	point	point mutation in *plu0267* leading to amino acid replacement N134K
4	544022	G	544023	A	point	point mutation in *plu0497* leading to amino acid replacement K118E
u5	1324641-1324642	AA	1324642	A	shift	gene disrupted by frameshift in TT01; the N-term part of the corresponding protein is annotated as *plu1145*, the C-term part of the corresponding protein as *plu1144*; an undisrupted near-identical gene is *plu3697*
6	1697992	T	1697992	A	point	point mutation in *plu1414* leading to amino acid replacement S191C
7	1714304	G	1714304	A	point	intergenic

8	1714443-1714444	TT	1714443	T	shift	Disrupts the multiply affected gene *sdhC* between codons 36 and 37; in TT01, *sdhC* (*plu1426*) is annotated starting with the TTG for Leu-43
9	1714482	G	1714481	A	point	point mutation in the multiply affected gene *sdhC* (*plu1426*) leading to amino acid replacement G45E
10	2582950	T	2582949-2582950	TT	shift	gene disrupted by frameshift in TT01; the N-term part of the corresponding protein is annotated as *plu2195*, the C-term part of the corresponding protein as *plu2196*
11	2871215	G	2871215	C	point	point mutation in *plu2443* leading to amino acid replacement W139S
12	3103155-3103156	AA	3103155-3103157	AAA	shift	*plu2648* lacks the N-terminal region of the corresponding protein, start codon out of frame
13	3272404-3272407	CCCC	3272405-3272409	CCCCC	shift	*plu2762* contains an aberrant N-terminal region of the corresponding protein
14	3374998-3375301	TTAGTTTA (38)	3375000-3375311	TTAGTTTA (39)	copy-nr	
15	3508347-3514141	5795 bp (fwd)	3514151-3508357	5795 bp (rev)	inversion	bounded by a 35 bp inverted repeat; on prophage R14
16	4207421-4207429	TTTTACAGG (46) TTAAAACTATCG (11)	4207431-4207436	TTTTACAGG (47) TTAAAACTATCG (10)	copy-nr	
17	4236070-4244938	8869 bp	4236076/4236077	-	indel	an insertion in TT01m (PhRepA_D) of which 1492 bp represent a copy of ISPlu9
18	4358242/4358243	-	4349381-4359525	10145 bp	indel	a deletion in TT01m (PhRepA_E) of which 1492 bp represent a copy of ISPlu9; prophage R17
19	4778231-4778342	TAGTTATTG AAGTAAA (7)	4779514-4779641	TAGTTATTG AAGTAAA (8)	copy-nr	*plu4091* terminates within the first copy of the repeat
20	4861732	C	4863031	A	point	within a copy of PhoLumMITEPlu5
21	4863021	C	4864320	A	point	ISPlu20 (deviates from canonical ISPlu20 sequence only in TT01)
22	5259405	A	5260704	T	point	intergenic
23	5259440	C	5260739-5260740	TA	diff	affects the C-terminal region of the corresponding protein of the multiply affected gene *plu4505* in TT01
24	5259451-5259452	AA	5260751-5260753	GAC	diff	leads to an aberrant C-terminal region in the corresponding protein of the multiply affected gene *plu4505* in TT01

25	5259487-5259489	AGC	5260788-5260790	TTT	diff	point mutation in *plu4504* leading to amino acid replacement K62L
26	5261212	C	5262513	T	point	silent mutation in *plu4506*
27	5350422-5350424	CCC	5351723-5351726	CCCC	shift	*plu4585* contains an aberrant region encoding the C-terminal part of the corresponding protein
28	5472344	C	5473646	G	point	in 16S rRNA
29	5529801-5530328	TAAGCAAA (20) TAAGTAAA (46)	5531103-5531638	TAAGCAAA (20) TAAGTAAA (47)	copy-nr	
30	5560378	A	5561688	G	point	silent mutation in *plu4782*

Supplementary Table S2: Genome comparison between *P. luminescens* strain DJC and TT01 (as represented by the newly sequenced genome TT01m). The genome is split into matching segments (matchSEG) and divergent segments (divSEGs). Each matchSEG is identified by its serial number (nr). DivSEGs carry the same number as the subsequent matchSEG. They are categorized into four distinct types: (indel, approximate insert (approxI), replace, copynr) (see below for an explanation). matchSEGs and the type of divSEG are indicated in the "type" column. Between each pair of matchSEGs is a divSEG with few exceptions (matchSEGs 3-5, 147-149, and 214-216). In these cases, the central matchSEG is inverted between the two strains without any separating divSEG (inversions are mention in the comment column). For each strain, the genome position (pos) and the length (length) is given. For inverted matchSEGs, reverse orientation is indicated by /R for the TT01m genome. For matchSEGs, the percent sequence difference is given (%diff). The majority of matchSEGs show less than 1% sequence difference. For the matchSEGs with more than 1% sequence difference, which tend to be relatively short, more details are provided in the comment column (allowed terms are DissimGlobal, DissimLocal, DissimButShort) (see below for an explanation). For each matchSEG, the difference details are provided as a set of four integers (see below for an explanation). All divSEGs have explanatory information in the comment column. Explanation details: (a) terms for matchSEGs with more than 1% sequence difference: DissimButShort indicates a short segment (below 2 kb). Longer matchSEGs may have a cluster of divergence (DissimLocal) or may have a generally increased mutation rate (DissimGlobal); (b) the four numbers for the difference details are the alignment bases, the number of point mutations, the number of gap open characters, and the number of gap extension characters; (c) types of divSEGs are: (c1) indel: there are extra bases in one strain while the genome is contigous in the other, allowing an exact positioning of the insert; (c2) approximate insert: an insert which cannot be exactly positioned; restriction to approximate positioning is due to a few unaligned bases (maximally 10) at the insertion site; (c3) replace: there are additional sequences in both strains and these are too unrelated to be considered a matchSEG; (c4) copynr: there are short tandem repeats (7-12 bp) with a differing number of copies between the strains; (d) explanations of divergent segments: (d1) in case of copynr, the tandem repeat is given and the number of copies in the two genomes (TT01m and DJC). Otherwise, the region is briefly described by a short term; (d2) mobile genetic elements are flagged by MGE:ISPlu, MGE:MITEPlu, or MGE:IRS plus the serial number of the element. ISPlu refers to

elements found in the IS section of ISFinder, MITEPlu to those in the MITE section, while IRS (insertion related sequence) is used for other elements. The extension "+TSD", used only for type indel, indicates that the element is bounded by a target site duplication; (d3) for CRISPRs, counts are provided for strain-specific spacers (tagged extra), for matching spacers (tagged shared) and for multiple occurences of an identical spacer (tagged duplicated). The partial deletion of cas1 is indicated (cas1_truncated as opposed to cas1_complete); (d4) for indel, approximate insert, and replace, the sequences are categorized by terms "specific" (strain-specific, may be completely unrelated or may have up to ca 85% sequence identity), "SeqRelated" (sequences have more than 85% sequence identity but below 98% seq_id), "InternallyRepeated" (a genome-internal duplication so that a highly related sequence is found elsewhere), "HighSimElsewhere" (a near-identical sequence which is found at an unrelated genome position in the other strain, precluding its classification as matchSEG) or "FewAdjacent" (which indicates a short extra region of less than 100 bp outside a mobile genetic element). A small number of divSEGs are tagged by a more extended wording, including a reference to an atypical sequence of 121 bp which is found exclusively in a subset of the 23S rRNAs of strain TT01. For long insertions or long replacement sequences, overlaps with prophage regions (see Supplementary Table S5) are indicated. Whenever appropriate, a relation to the long repeat PhRepA is indicated in the explanations.

		***P. luminescens* TT01m**		***P. luminescens* DJC**				
**nr**	**type**	**pos**	**length**	**pos**	**length**	**%diff**	**difference details**	**comments**
1	matchSEG	1-7476/F	7476	1-7476/F	7476	0.12	7476/9/0/0	-
2	replace	7477-8092/F	616	7477-8220/F	744	-	-	TT01m:InternallyRepeated ; DJC:specific+MGE:MITEPlu5
2	matchSEG	8093-23764/F	15672	8221-23893/F	15673	0.11	15673/17/1/0	-
3	indel	23765-23889/F	125	-	-	-	-	MGE:MITEPlu5+TSD
3	matchSEG	23890-26554/F	2665	23894-26558/F	2665	0.30	2665/8/0/0	-

4	matchSEG	26555-29975/R	3421	26559-29986/F	3428	6.19	3423/210/2/5	DissimLocal+inverted (in TT01m inverted, in both, TT01 and DJC not)(prophage R01)
5	matchSEG	29976-102020/F	72045	29987-102031/F	72045	0.19	72045/138/0/0	-
6	replace	102021-102553/F	533	102032-112272/F	10241	-	-	TT01m:specific+MGE:MITEPlu5 ; DJC:specific+MGE:multi (prophage R01)
6	matchSEG	102554-158090/F	55537	112273-167804/F	55532	0.23	55534/123/4/5	-
7	indel	-	-	167805-167929/F	125	-	-	MGE:MITEPlu5+TSD
7	matchSEG	158091-294110/F	136020	167930-303989/F	136060	0.12	136021/161/6/44	-
8	indel	-	-	303990-304136/F	147	-	-	specific
8	matchSEG	294111-341841/F	47731	304137-351854/F	47718	0.28	47719/131/2/13	-
9	indel	341842-343615/F	1774	-	-	-	-	MGE:ISPlu4+TSD
9	matchSEG	343616-347423/F	3808	351855-355662/F	3808	0.32	3808/12/0/0	-
10	indel	347424-348522/F	1099	-	-	-	-	MGE:ISPlu19+TSD
10	matchSEG	348523-361100/F	12578	355663-368237/F	12575	0.44	12576/54/1/2	-
11	indel	361101-386643/F	25543	-	-	-	-	InternallyRepeated+MGE:multi (prophage R02)
11	matchSEG	386644-415430/F	28787	368238-397024/F	28787	0.01	28787/3/0/0	-
12	indel	415431-416581/F	1151	-	-	-	-	MGE:ISPlu10+TSD
12	matchSEG	416582-430275/F	13694	397025-410718/F	13694	0.15	13694/20/0/0	-
13	indel	430276-431271/F	996	-	-	-	-	MGE:ISPlu6+TSD
13	matchSEG	431272-501718/F	70447	410719-481167/F	70449	0.13	70449/91/2/0	-

14	indel	501719-502869/F	1151	-	-	-	-	MGE:ISPlu10+TSD
14	matchSEG	502870-503343/F	474	481168-481641/F	474	0.00	474/0/0/0	-
15	copynr	503344-503523/F	180	481642-481731/F	90	-	-	CTGTTAAAG(TT01m:20/ DJC:10)
15	matchSEG	503524-572180/F	68657	481732-550388/F	68657	0.02	68657/17/0/0	-
16	indel	-	-	550389-551487/F	1099	-	-	MGE:ISPlu19+TSD
16	matchSEG	572181-590415/F	18235	551488-569749/F	18262	0.53	18224/93/4/53	-
17	replace	590416-598115/F	7700	569750-571766/F	2017	-	-	TT01m: specific+InternallyRepeated+SeqRelated ; DJC:specific+SeqRelated
17	matchSEG	598116-598561/F	446	571767-572207/F	441	4.30	442/18/1/4	DissimButShort
18	indel	-	-	572208-573707/F	1500	-	-	MGE:ISPlu9+TSD
18	matchSEG	598562-610041/F	11480	573708-585187/F	11480	1.65	11480/189/0/0	DissimLocal(PhRepA)
19	indel	-	-	585188-587534/F	2347	-	-	MGE:ISPlu20+TSD
19	matchSEG	610042-633621/F	23580	587535-611066/F	23532	0.37	23533/86/1/47	-
20	approxI	633622-633625/F	4	611067-611193/F	127	-	-	TT01m:FewUnaligned ; DJC:MGE:MITEPlu5
20	matchSEG	633626-641736/F	8111	611194-619304/F	8111	0.49	8111/40/0/0	-
21	replace	641737-641861/F	125	619305-619332/F	28	-	-	TT01m:MGE:MITEPlu5 ; DJC:specific
21	matchSEG	641862-642044/F	183	619333-619515/F	183	11.96	184/20/2/0	DissimButShort
22	indel	-	-	619516-619640/F	125	-	-	MGE:MITEPlu5+TSD
22	matchSEG	642045-642379/F	335	619641-619975/F	335	4.18	335/14/0/0	DissimButShort

23	indel	-	-	619976-621127/F	1152	-	-	MGE:ISPlu16+TSD
23	matchSEG	642380-643274/F	895	621128-622022/F	895	7.71	895/69/0/0	DissimButShort
24	replace	643275-643893/F	619	622023-622085/F	63	-	-	TT01m:InternallyRepeated+MGE:MITEPlu5 ; DJC:remnant of MGE:MITEPlu5
24	matchSEG	643894-727267/F	83374	622086-705477/F	83392	0.32	83375/262/2/18	-
25	indel	-	-	705478-718227/F	12750	-	-	InternallyRepeated
25	matchSEG	727268-804557/F	77290	718228-795513/F	77286	0.03	77287/25/1/3	-
26	replace	804558-804678/F	121	795514-795524/F	11	-	-	TT01m:an atypical 121 bp insert in copies C and E of 23S rRNA ; DJC:contains the typical 11 bp region found in nearly all 23S rRNAs
26	matchSEG	804679-808546/F	3868	795525-799392/F	3868	0.21	3868/8/0/0	-
27	indel	-	-	799393-802317/F	2925	-	-	MGE:ISPlu10+TSD (ISPlu10 targeted by MGE:ISPlu4)
27	matchSEG	808547-827866/F	19320	802318-821637/F	19320	0.04	19320/7/0/0	-
28	replace	827867-829937/F	2071	821638-823445/F	1808	-	-	TT01m:specific+SeqRelated+MGE:ISPlu19+MGE:MITEPlu5 ; DJC:specific+SeqRelated+MGE:MITEPlu5
28	matchSEG	829938-847286/F	17349	823446-840798/F	17353	0.14	17351/23/2/2	-
29	indel	847287-848437/F	1151	-	-	-	-	MGE:ISPlu10+TSD
29	matchSEG	848438-927729/F	79292	840799-920078/F	79280	0.10	79252/72/7/75	-
30	indel	-	-	920079-920203/F	125	-	-	MGE:MITEPlu5+TSD
30	matchSEG	927730-984397/F	56668	920204-976959/F	56756	0.25	56672/137/5/85	-

31	indel	984398-985483/F	1086	-	-	-	-	MGE:ISPlu1+TSD
31	matchSEG	985484-991486/F	6003	976960-982962/F	6003	0.08	6003/5/0/0	-
32	replace	991487-992353/F	867	982963-983901/F	939	-	-	TT01m:specific ; DJC:InternallyRepeated
32	matchSEG	992354-994509/F	2156	983902-986068/F	2167	0.42	2158/7/2/9	-
33	indel	-	-	986069-986171/F	103	-	-	specific
33	matchSEG	994510-994769/F	260	986172-986431/F	260	0.77	260/2/0/0	-
34	indel	-	-	986432-987582/F	1151	-	-	MGE:ISPlu10+TSD
34	matchSEG	994770-994865/F	96	987583-987678/F	96	0.00	96/0/0/0	-
35	copynr	994866-995019/F	154	987679-987762/F	84	-	-	TATAATC(TT01m:22/ DJC:12)
35	matchSEG	995020-1035902/F	40883	987763-1028645/F	40883	0.01	40883/4/0/0	-
36	indel	-	-	1028646-1028770/F	125	-	-	MGE:MITEPlu5+TSD
36	matchSEG	1035903-1057588/F	21686	1028771-1050470/F	21700	0.14	21687/30/1/13	-
37	indel	1057589-1057713/F	125	-	-	-	-	MGE:MITEPlu5+TSD
37	matchSEG	1057714-1063159/F	5446	1050471-1055893/F	5423	0.85	5424/45/1/22	-
38	indel	1063160-1063284/F	125	-	-	-	-	MGE:MITEPlu5+TSD
38	matchSEG	1063285-1065823/F	2539	1055894-1058440/F	2547	2.20	2540/55/1/7	DissimLocal

39	copynr	1065824-1065968/F	145	1058441-1058617/F	177	-	-	CTGTAAAA(TT01m:18/ DJC:22)
39	matchSEG	1065969-1069039/F	3071	1058618-1061688/F	3071	1.56	3071/48/0/0	DissimLocal
40	indel	-	-	1061689-1063451/F	1763	-	-	MGE:IRS113
40	matchSEG	1069040-1069484/F	445	1063452-1063896/F	445	4.72	445/21/0/0	DissimButShort
41	indel	1069485-1072895/F	3411	-	-	-	-	InternallyRepeated
41	matchSEG	1072896-1079629/F	6734	1063897-1070630/F	6734	0.07	6734/5/0/0	-
42	indel	-	-	1070631-1070755/F	125	-	-	MGE:MITEPlu5+TSD
42	matchSEG	1079630-1151793/F	72164	1070756-1142921/F	72166	0.09	72166/66/2/0	-
43	indel	-	-	1142922-1143046/F	125	-	-	MGE:MITEPlu5
43	matchSEG	1151794-1177029/F	25236	1143047-1168308/F	25262	0.18	25237/44/1/25	-
44	replace	1177030-1178363/F	1334	1168309-1170646/F	2338	-	-	TT01m:specific+SeqRelated+MGE:MITEPlu5 ; DJC:specific+SeqRelated+MGE:ISPlu16
44	matchSEG	1178364-1238448/F	60085	1170647-1230731/F	60085	0.03	60085/20/0/0	-
45	indel	1238449-1240795/F	2347	-	-	-	-	MGE:ISPlu20+TSD

45	matchSEG	1240796-1249089/F	8294	1230732-1239025/F	8294	0.01	8294/1/0/0	-
46	indel	1249090-1328832/F	79743	-	-	-	-	specific+InternallyRepeated+MGE:multi (prophage R04)
46	matchSEG	1328833-1459482/F	130650	1239026-1369700/F	130675	0.22	130642/288/4/45	-
47	indel	-	-	1369701-1369822/F	122	-	-	MGE:IRS112+TSD
47	matchSEG	1459483-1494375/F	34893	1369823-1404756/F	34934	0.55	34892/189/4/47	-
48	indel	-	-	1404757-1404881/F	125	-	-	MGE:MITEPlu5+TSD
48	matchSEG	1494376-1534135/F	39760	1404882-1444641/F	39760	0.09	39760/36/0/0	-
49	indel	-	-	1444642-1444766/F	125	-	-	MGE:MITEPlu5+TSD
49	matchSEG	1534136-1561668/F	27533	1444767-1472299/F	27533	0.07	27533/19/0/0	-
50	indel	1561669-1574013/F	12345	-	-	-	-	InternallyRepeated (prophage R05)
50	matchSEG	1574014-1588775/F	14762	1472300-1487069/F	14770	0.01	14763/0/1/7	(prophage R05)
51	indel	1588776-1600599/F	11824	-	-	-	-	InternallyRepeated (prophage R05)
51	matchSEG	1600600-1625285/F	24686	1487070-1511755/F	24686	0.05	24686/13/0/0	-

52	approxI	1625286-1626789/F	1504	1511756-1511758/F	3	-	-	TT01m:FewUnaligned ; DJC:MGE:ISPlu9+TSD
52	matchSEG	1626790-1629188/F	2399	1511759-1514157/F	2399	1.58	2399/38/0/0	DissimLocal(PhRepA) (prophage R05)
53	indel	-	-	1514158-1517742/F	3585	-	-	InternallyRepeated(PhRepA)
53	matchSEG	1629189-1684260/F	55072	1517743-1572884/F	55142	0.14	55073/75/1/69	-
54	indel	-	-	1572885-1572995/F	111	-	-	111-mer tandem repeated in DJC but not in TT01m
54	matchSEG	1684261-1697419/F	13159	1572996-1586154/F	13159	0.15	13159/20/0/0	-
55	approxI	1697420-1697429/F	10	1586155-1586284/F	130	-	-	TT01m:FewUnaligned ; DJC:MGE:MITEPlu5
55	matchSEG	1697430-1698399/F	970	1586285-1587256/F	972	1.85	971/17/1/1	DissimButShort
56	indel	-	-	1587257-1587439/F	183	-	-	MGE:MITEPlu5+TSD
56	matchSEG	1698400-1700078/F	1679	1587440-1589117/F	1678	2.08	1679/34/1/0	DissimLocal
57	approxI	1700079-1700080/F	2	1589118-1590205/F	1088	-	-	TT01m:FewUnaligned ; DJC:MGE:ISPlu1+FewAdjacent
57	matchSEG	1700081-1708742/F	8662	1590206-1598898/F	8693	0.84	8664/71/2/29	-
58	indel	1708743-1709841/F	1099	-	-	-	-	MGE:ISPlu19+TSD

58	matchSEG	1709842-1766768/F	56927	1598899-1655823/F	56925	0.11	56926/59/2/2	-
59	indel	1766769-1766893/F	125	-	-	-	-	MGE:MITEPlu5+TSD
59	matchSEG	1766894-1774954/F	8061	1655824-1663884/F	8061	0.26	8061/21/0/0	-
60	replace	1774955-1776793/F	1839	1663885-1665326/F	1442	-	-	TT01m:specific+MGE:MITEPlu5+MGE:IRS66 ; DJC:specific
60	matchSEG	1776794-1781918/F	5125	1665327-1670451/F	5125	0.23	5125/12/0/0	-
61	indel	1781919-1782047/F	129	-	-	-	-	MGE:MITEPlu5+FewAdjacent
61	matchSEG	1782048-1812837/F	30790	1670452-1701271/F	30820	0.21	30791/65/1/29	-
62	replace	1812838-1815842/F	3005	1701272-1702044/F	773	-	-	TT01m:specific+SeqRelated ; DJC:specific+SeqRelated
62	matchSEG	1815843-1821817/F	5975	1702045-1708015/F	5971	1.66	5974/96/3/1	DissimLocal
63	replace	1821818-1823965/F	2148	1708016-1708419/F	404	-	-	TT01m:specific+MGE:ISPlu14+MGE:MITEPlu5 ; DJC:SeqRelated
63	matchSEG	1823966-1919226/F	95261	1708420-1803599/F	95180	0.05	95181/47/1/80	-
64	replace	1919227-1925020/F	5794	1803600-1860888/F	57289	-	-	TT01m:specific+MGE:multi ; DJC:specific+InternallyRepeated(indel_077)+MGE:IRS1 (prophage R06)

64	matchSEG	1925021-1965328/F	40308	1860889-1901183/F	40295	0.19	40297/74/2/11	-
65	indel	1965329-1966325/F	997	-	-	-	-	MGE:ISPlu6+TSD
65	matchSEG	1966326-2023521/F	57196	1901184-1958391/F	57208	0.10	57197/59/1/11	-
66	indel	2023522-2024620/F	1099	-	-	-	-	MGE:ISPlu19+TSD
66	matchSEG	2024621-2065555/F	40935	1958392-1999329/F	40938	0.05	40931/17/2/13	-
67	indel	-	-	1999330-2000481/F	1152	-	-	MGE:ISPlu16+TSD
67	matchSEG	2065556-2081255/F	15700	2000482-2016174/F	15693	0.31	15694/47/1/6	-
68	approxI	2081256-2081265/F	10	2016175-2016296/F	122	-	-	TT01m:FewUnaligned ; DJC:MGE:MITEPlu5
68	matchSEG	2081266-2081788/F	523	2016297-2016809/F	513	1.56	514/7/1/9	DissimButShort
69	indel	2081789-2083562/F	1774	-	-	-	-	MGE:ISPlu4+TSD
69	matchSEG	2083563-2086293/F	2731	2016810-2019587/F	2778	0.22	2733/4/2/45	-
70	replace	2086294-2086643/F	350	2019588-2019947/F	360	-	-	TT01m:specific+SeqRelated+MGE:MITEPlu5 ; DJC:specific+SeqRelated
70	matchSEG	2086644-2101375/F	14732	2019948-2034686/F	14739	1.26	14708/181/4/59	DissimLocal

71	indel	2101376-2102371/F	996	-	-	-	-	MGE:ISPlu6+TSD
71	matchSEG	2102372-2119117/F	16746	2034687-2051432/F	16746	0.08	16746/13/0/0	-
72	indel	2119118-2119252/F	135	-	-	-	-	MGE:MITEPlu5+FewAdjacent
72	matchSEG	2119253-2126977/F	7725	2051433-2059186/F	7754	0.69	7723/51/2/35	-
73	indel	-	-	2059187-2059306/F	120	-	-	CRISPRdiff(2extra)
73	matchSEG	2126978-2130960/F	3983	2059307-2063228/F	3922	0.59	3924/21/2/59	-
74	indel	2130961-2132059/F	1099	-	-	-	-	MGE:ISPlu3+TSD
74	matchSEG	2132060-2132809/F	750	2063229-2063978/F	750	2.13	751/14/2/0	DissimButShort
75	indel	-	-	2063979-2064566/F	588	-	-	MGE:ISPlu2
75	matchSEG	2132810-2133410/F	601	2064567-2065167/F	601	0.67	601/4/0/0	-
76	replace	2133411-2133558/F	148	2065168-2066732/F	1565	-	-	TT01m:CRISPRdiff(2extra)+cas1_truncated ; DJC:CRISPRdiff(8extra)+cas1_complete
76	matchSEG	2133559-2134732/F	1174	2066733-2067897/F	1165	4.37	1166/50/1/8	DissimButShort
77	indel	-	-	2067898-2100511/F	32614	-	-	specific+InternallyRepeated(replace_064)+MGE:IRS1 (prophage R08)

77	matchSEG	2134733-2142057/F	7325	2100512-2107836/F	7325	0.72	7325/53/0/0	-
78	indel	-	-	2107837-2108076/F	240	-	-	CRISPRdiff(4extra)
78	matchSEG	2142058-2142385/F	328	2108077-2108404/F	328	0.00	328/0/0/0	-
79	indel	2142386-2142685/F	300	-	-	-	-	CRISPRdiff(5extra)
79	matchSEG	2142686-2145305/F	2620	2108405-2111027/F	2623	4.04	2621/105/1/2	DissimLocal
80	indel	-	-	2111028-2111163/F	136	-	-	MGE:MITEPlu5+FewAdjacent
80	matchSEG	2145306-2157410/F	12105	2111164-2123268/F	12105	2.45	12105/296/0/0	DissimGlobal
81	indel	2157411-2157535/F	125	-	-	-	-	MGE:MITEPlu5+TSD
81	matchSEG	2157536-2167317/F	9782	2123269-2133057/F	9789	0.21	9783/20/1/6	-
82	indel	2167318-2168313/F	996	-	-	-	-	MGE:ISPlu6+TSD
82	matchSEG	2168314-2176804/F	8491	2133058-2141548/F	8491	0.00	8491/0/0/0	-
83	indel	2176805-2183354/F	6550	-	-	-	-	specific
83	matchSEG	2183355-2206517/F	23163	2141549-2164711/F	23163	0.02	23163/5/0/0	-

84	indel	2206518-2206640/F	123	-	-	-	-	MGE:MITEPlu5
84	matchSEG	2206641-2207756/F	1116	2164712-2165827/F	1116	0.09	1116/1/0/0	-
85	replace	2207757-2221565/F	13809	2165828-2169455/F	3628	-	-	TT01m:specific+SeqRelated+MGE:multi ; DJC:specific+SeqRelated+MGE:multi
85	matchSEG	2221566-2268059/F	46494	2169456-2215944/F	46489	0.15	46490/69/1/4	-
86	indel	2268060-2271136/F	3077	-	-	-	-	InternallyRepeated
86	matchSEG	2271137-2338602/F	67466	2215945-2283386/F	67442	0.01	67443/6/1/23	-
87	indel	-	-	2283387-2283974/F	588	-	-	MGE:ISPlu2
87	matchSEG	2338603-2373147/F	34545	2283975-2318445/F	34471	0.14	34459/44/4/102	-
88	indel	-	-	2318446-2319033/F	588	-	-	MGE:ISPlu2
88	matchSEG	2373148-2392119/F	18972	2319034-2338005/F	18972	0.01	18972/1/0/0	-
89	replace	2392120-2392179/F	60	2338006-2338366/F	361	-	-	TT01m:CRISPRdiff(1shared) ; DJC:CRISPRdiff(5extra,1shared)
89	matchSEG	2392180-2395499/F	3320	2338367-2341686/F	3320	0.00	3320/0/0/0	-
90	replace	2395500-2395799/F	300	2341687-2341806/F	120	-	-	TT01m:CRISPRdiff(2extra,2shared,1duplicated) ; DJC:CRISPRdiff(2extra,2shared)

90	matchSEG	2395800-2396028/F	229	2341807-2342035/F	229	0.44	229/1/0/0	-
91	indel	-	-	2342036-2376474/F	34439	-	-	specific+InternallyRepeated (prophage R10)
91	matchSEG	2396029-2400961/F	4933	2376475-2381407/F	4933	0.12	4933/6/0/0	-
92	indel	2400962-2401086/F	125	-	-	-	-	MGE:MITEPlu5+TSD
92	matchSEG	2401087-2405369/F	4283	2381408-2385652/F	4245	0.64	4247/25/2/36	-
93	replace	2405370-2406745/F	1376	2385653-2387110/F	1458	-	-	TT01m:specific+SeqRelated ; DJC:specific+SeqRelated
93	matchSEG	2406746-2409581/F	2836	2387111-2389946/F	2836	1.23	2836/35/0/0	DissimLocal
94	replace	2409582-2409724/F	143	2389947-2390866/F	920	-	-	TT01m:MGE:MITEPlu5+FewAdjacent ; DJC:specific+MGE:MITEPlu5
94	matchSEG	2409725-2418046/F	8322	2390867-2399188/F	8322	0.14	8322/12/0/0	-
95	indel	2418047-2419145/F	1099	-	-	-	-	MGE:ISPlu3+TSD
95	matchSEG	2419146-2427929/F	8784	2399189-2407963/F	8775	0.13	8776/10/1/8	-
96	indel	-	-	2407964-2408088/F	125	-	-	MGE:MITEPlu5+TSD
96	matchSEG	2427930-2433968/F	6039	2408089-2414110/F	6022	1.13	6023/67/1/16	DissimLocal

97	indel	-	-	2414111-2414782/F	672	-	-	InternallyRepeated
97	matchSEG	2433969-2475431/F	41463	2414783-2456241/F	41459	0.17	41460/68/1/3	-
98	replace	2475432-2475703/F	272	2456242-2457815/F	1574	-	-	TT01m:CRISPRdiff(5extra) ; DJC:CRISPRdiff(27extra)
98	matchSEG	2475704-2476256/F	553	2457816-2458394/F	579	11.03	553/59/2/28	DissimButShort
99	replace	2476257-2476519/F	263	2458395-2458493/F	99	-	-	TT01m:specific+MGE:MITEPlu5 ; DJC:InternallyRepeated
99	matchSEG	2476520-2484138/F	7619	2458494-2466131/F	7638	0.25	7620/17/2/19	-
100	indel	2484139-2484263/F	125	-	-	-	-	MGE:MITEPlu5+TSD
100	matchSEG	2484264-2528318/F	44055	2466132-2510187/F	44056	0.06	44056/26/1/0	-
101	indel	2528319-2528443/F	125	-	-	-	-	MGE:MITEPlu5
101	matchSEG	2528444-2537638/F	9195	2510188-2519382/F	9195	0.24	9195/22/0/0	-
102	approxI	2537639-2537639/F	1	2519383-2520461/F	1079	-	-	TT01m:OneUnaligned ; DJC:specific
102	matchSEG	2537640-2576718/F	39079	2520462-2559550/F	39089	0.10	39082/35/4/8	-
103	indel	2576719-2576899/F	181	-	-	-	-	specific

103	matchSEG	2576900-2577127/F	228	2559551-2559778/F	228	0.88	228/2/0/0	-
104	indel	2577128-2581898/F	4771	-	-	-	-	specific
104	matchSEG	2581899-2582817/F	919	2559779-2560697/F	919	4.02	920/35/2/0	DissimButShort
105	indel	2582818-2586153/F	3336	-	-	-	-	specific
105	matchSEG	2586154-2586539/F	386	2560698-2561083/F	386	0.52	386/2/0/0	-
106	indel	2586540-2589438/F	2899	-	-	-	-	specific+MGE:IRS112
106	matchSEG	2589439-2597529/F	8091	2561084-2569174/F	8091	3.09	8091/250/0/0	DissimGlobal
107	replace	2597530-2598953/F	1424	2569175-2569232/F	58	-	-	TT01m:InternallyRepeated ; DJC:specific
107	matchSEG	2598954-2600653/F	1700	2569233-2570932/F	1700	4.53	1700/77/0/0	DissimButShort
108	replace	2600654-2604306/F	3653	2570933-2573366/F	2434	-	-	TT01m:specific ; DJC:specific
108	matchSEG	2604307-2604413/F	107	2573367-2573473/F	107	1.87	107/2/0/0	DissimButShort
109	replace	2604414-2604534/F	121	2573474-2573515/F	42	-	-	TT01m:MGE:MITEPlu5 ; DJC: InternallyRepeated

109	matchSEG	2604535-2604961/F	427	2573516-2573884/F	369	8.60	372/29/3/55	DissimButShort
110	indel	2604962-2605086/F	125	-	-	-	-	MGE:MITEPlu5+TSD
110	matchSEG	2605087-2614481/F	9395	2573885-2583279/F	9395	1.03	9395/97/0/0	DissimLocal
111	replace	2614482-2614510/F	29	2583280-2583587/F	308	-	-	TT01m:specific ; DJC:specific
111	matchSEG	2614511-2615185/F	675	2583588-2584367/F	780	2.97	674/16/4/111	DissimButShort
112	indel	-	-	2584368-2585068/F	701	-	-	specific+MGE:MITEPlu5
112	matchSEG	2615186-2620126/F	4941	2585069-2590009/F	4941	0.12	4941/6/0/0	-
113	approxI	2620127-2620128/F	2	2590010-2590147/F	138	-	-	TT01m:FewUnaligned ; DJC:MGE:MITEPlu5+FewAdjacent
113	matchSEG	2620129-2621190/F	1062	2590148-2591209/F	1062	0.47	1062/5/0/0	-
114	replace	2621191-2621351/F	161	2591210-2591278/F	69	-	-	TT01m:specific ; DJC:specific
114	matchSEG	2621352-2646906/F	25555	2591279-2616833/F	25555	0.07	25555/18/0/0	-
115	replace	2646907-2649865/F	2959	2616834-2619783/F	2950	-	-	TT01m:specific+SeqRelated+MGE:ISPlu11+TSD ; DJC:specific+SeqRelated
115	matchSEG	2649866-2663034/F	13169	2619784-2632938/F	13155	0.55	13152/69/3/23	-

116	replace	2663035-2664518/F	1484	2632939-2632968/F	30	-	-	TT01m:specific+MGE:MITEPlu5 ; DJC:specific
116	matchSEG	2664519-2677661/F	13143	2632969-2646110/F	13142	0.04	13143/4/1/0	-
117	indel	2677662-2678747/F	1086	-	-	-	-	MGE:ISPlu1+TSD
117	matchSEG	2678748-2710860/F	32113	2646111-2678187/F	32077	0.26	32079/81/2/34	-
118	indel	2710861-2711959/F	1099	-	-	-	-	MGE:ISPlu3+TSD
118	matchSEG	2711960-2786856/F	74897	2678188-2753064/F	74877	0.20	74878/139/10/28	-
119	replace	2786857-2787652/F	796	2753065-2753148/F	84	-	-	TT01m:specific ; DJC:specific
119	matchSEG	2787653-2834238/F	46586	2753149-2799715/F	46567	0.03	46568/13/1/18	-
120	indel	2834239-2835389/F	1151	-	-	-	-	MGE:ISPlu10+TSD
120	matchSEG	2835390-2883434/F	48045	2799716-2847788/F	48073	0.51	48018/242/5/87	-
121	indel	-	-	2847789-2848376/F	588	-	-	MGE:ISPlu2
121	matchSEG	2883435-2908250/F	24816	2848377-2873192/F	24816	0.01	24816/3/0/0	-
122	approxI	2908251-2918654/F	10404	2873193-2873196/F	4	-	-	TT01m:specific+InternallyRepeated+MGE:multi ; DJC:FewUnaligned

122	matchSEG	2918655-2981457/F	62803	2873197-2935980/F	62784	0.26	62771/152/10/55	-
123	indel	-	-	2935981-2936105/F	125	-	-	MGE:MITEPlu5+TSD
123	matchSEG	2981458-3007796/F	26339	2936106-2962434/F	26329	0.06	26330/15/1/9	-
124	indel	-	-	2962435-2962559/F	125	-	-	MGE:MITEPlu5+TSD
124	matchSEG	3007797-3011590/F	3794	2962560-2966349/F	3790	0.32	3791/11/1/3	-
125	indel	3011591-3011693/F	103	-	-	-	-	MGE: MITEPlu2+TSD
125	matchSEG	3011694-3011792/F	99	2966350-2966448/F	99	1.01	99/1/0/0	DissimButShort
126	indel	3011793-3011917/F	125	-	-	-	-	MGE:MITEPlu5+TSD
126	matchSEG	3011918-3015578/F	3661	2966449-2970109/F	3661	0.68	3661/25/0/0	-
127	replace	3015579-3016374/F	796	2970110-2972199/F	2090	-	-	TT01m:InternallyRepeated+SeqRelated ; DJC:InternallyRepeated+SeqRelated+MGE:ISPlu10
127	matchSEG	3016375-3021310/F	4936	2972200-2977134/F	4935	0.61	4936/29/1/0	-
128	indel	-	-	2977135-2977259/F	125	-	-	MGE:MITEPlu5+TSD
128	matchSEG	3021311-3035442/F	14132	2977260-2991391/F	14132	0.33	14132/46/0/0	-
129	indel	-	-	2991392-2991515/F	124	-	-	MGE:MITEPlu5

129	matchSEG	3035443-3096501/F	61059	2991516-3052575/F	61060	0.30	61061/183/3/0	-
130	indel	-	-	3052576-3053498/F	923	-	-	MGE:ISPlu7+TSD
130	matchSEG	3096502-3103995/F	7494	3053499-3061008/F	7510	4.42	7496/329/2/14	DissimGlobal
131	replace	3103996-3104659/F	664	3061009-3061093/F	85	-	-	TT01m:specific+MGE:MITEPlu5 ; DJC:specific
131	matchSEG	3104660-3105848/F	1189	3061094-3062279/F	1186	6.09	1183/68/4/13	DissimButShort
132	indel	-	-	3062280-3063430/F	1151	-	-	MGE:ISPlu10+TSD
132	matchSEG	3105849-3106370/F	522	3063431-3063978/F	548	7.06	524/33/4/26	DissimButShort
133	approxI	3106371-3106497/F	127	3063979-3063980/F	2	-	-	TT01m:MGE:MITEPlu5 ; DJC:FewUnaligned
133	matchSEG	3106498-3107025/F	528	3063981-3064409/F	429	8.56	432/33/4/97	DissimButShort
134	indel	-	-	3064410-3064997/F	588	-	-	MGE:ISPlu2
134	matchSEG	3107026-3209689/F	102664	3064998-3167722/F	102725	0.72	102670/727/9/58	-
135	indel	-	-	3167723-3167847/F	125	-	-	MGE:MITEPlu5+TSD
135	matchSEG	3209690-3254327/F	44638	3167848-3212485/F	44638	0.11	44638/50/0/0	-
136	indel	3254328-3255426/F	1099	-	-	-	-	MGE:ISPlu19+TSD

136	matchSEG	3255427-3285184/F	29758	3212486-3242243/F	29758	0.09	29758/26/0/0	-
137	copynr	3285185-3285429/F	245	3242244-3242299/F	56	-	-	TTACAGA(TT01m:36/ DJC:16)
137	matchSEG	3285430-3327919/F	42490	3242300-3284773/F	42474	0.01	42460/1/3/47	-
138	indel	-	-	3284774-3285872/F	1099	-	-	MGE:ISPlu3+TSD
138	matchSEG	3327920-3374997/F	47078	3285873-3332950/F	47078	0.01	47078/3/0/0	-
139	copynr	3374998-3375301/F	304	3332951-3333094/F	144	-	-	TTAGTTTA(TT01m:38/ DJC:18)
139	matchSEG	3375302-3392450/F	17149	3333095-3350243/F	17149	0.07	17149/12/0/0	-
140	indel	-	-	3350244-3350389/F	146	-	-	MGE:MITEPlu5+FewAdjacent
140	matchSEG	3392451-3409412/F	16962	3350390-3367392/F	17003	0.24	16963/40/1/40	-
141	indel	3409413-3456242/F	46830	-	-	-	-	specific(prophage R14)
141	matchSEG	3456243-3458735/F	2493	3367393-3369945/F	2553	0.60	2494/14/1/59	-
142	indel	3458736-3459834/F	1099	-	-	-	-	MGE:ISPlu3+TSD
142	matchSEG	3459835-3460194/F	360	3369946-3370304/F	359	4.17	360/14/1/0	DissimButShort

143	replace	3460195-3460321/F	127	3370305-3370584/F	280	-	-	TT01m:specific ; DJC:specific
143	matchSEG	3460322-3463572/F	3251	3370585-3373835/F	3251	0.40	3251/13/0/0	-
144	replace	3463573-3465146/F	1574	3373836-3373911/F	76	-	-	TT01m:specific ; DJC:specific
144	matchSEG	3465147-3468379/F	3233	3373912-3377144/F	3233	1.21	3233/39/0/0	DissimLocal
145	replace	3468380-3468748/F	369	3377145-3378556/F	1412	-	-	TT01m:specific+MGE:MITEPlu5 ; DJC:specific+MGE:MITEPlu5
145	matchSEG	3468749-3470269/F	1521	3378557-3380077/F	1521	0.66	1521/10/0/0	-
146	replace	3470270-3470872/F	603	3380078-3381245/F	1168	-	-	TT01m:specific+MGE:MITEPlu5 ; DJC:specific+MGE:MITEPlu5
146	matchSEG	3470873-3508332/F	37460	3381246-3418688/F	37443	0.57	37445/211/3/16	-
147	replace	3508333-3514107/F	5775	3418689-3421693/F	3005	-	-	TT01m:specific(prophage R14) ; DJC:specific (prophage R14)
147	matchSEG	3514108-3514935/F	828	3421694-3422521/F	828	3.86	828/32/0/0	DissimButShort(prophage)
148	matchSEG	3514936-3522867/R	7932	3422522-3430453/F	7932	0.23	7936/10/8/0	inverted between TT01m and DJC (prophage R14)
149	matchSEG	3522868-3536790/F	13923	3430454-3444375/F	13922	1.60	13924/220/3/0	DissimLocal(prophage R14)

150	indel	3536791-3536915/F	125	-	-	-	-	MGE:MITEPlu5+TSD
150	matchSEG	3536916-3563637/F	26722	3444376-3471105/F	26730	0.21	26713/55/2/28	-
151	indel	-	-	3471106-3472879/F	1774	-	-	MGE:ISPlu4+TSD
151	matchSEG	3563638-3617331/F	53694	3472880-3526573/F	53694	0.38	53694/205/0/0	-
152	indel	3617332-3618482/F	1151	-	-	-	-	MGE:ISPlu10+TSD
152	matchSEG	3618483-3663762/F	45280	3526574-3571849/F	45276	0.23	45272/103/2/14	-
153	indel	3663763-3663887/F	125	-	-	-	-	MGE:MITEPlu5+TSD
153	matchSEG	3663888-3745593/F	81706	3571850-3653542/F	81693	0.16	81696/131/3/10	-
154	indel	-	-	3653543-3653667/F	125	-	-	MGE:MITEPlu5+TSD
154	matchSEG	3745594-3769917/F	24324	3653668-3677982/F	24315	0.58	24306/138/3/30	-
155	approxI	3769918-3770049/F	132	3677983-3677989/F	7	-	-	TT01m:MGE:MITEPlu5 ; DJC:FewUnaligned
155	matchSEG	3770050-3777796/F	7747	3677990-3685739/F	7750	0.67	7748/51/1/2	-
156	indel	3777797-3778793/F	997	-	-	-	-	MGE:ISPlu6+TSD

156	matchSEG	3778794-3835798/F	57005	3685740-3742738/F	56999	0.49	57000/279/1/5	-
157	replace	3835799-3840551/F	4753	3742739-3747501/F	4763	-	-	TT01m:InternallyRepeated ; DJC:InternallyRepeated
157	matchSEG	3840552-3843354/F	2803	3747502-3750304/F	2803	0.89	2803/25/0/0	-
158	approxI	3843355-3843357/F	3	3750305-3750410/F	106	-	-	TT01m:FewUnaligned ; DJC:MGE: MITEPlu2+FewAdjacent
158	matchSEG	3843358-3857816/F	14459	3750411-3764869/F	14459	0.46	14459/67/0/0	-
159	indel	3857817-3860163/F	2347	-	-	-	-	MGE:ISPlu20+TSD
159	matchSEG	3860164-3886545/F	26382	3764870-3791251/F	26382	0.00	26382/0/0/0	-
160	indel	3886546-3887696/F	1151	-	-	-	-	MGE:ISPlu10+TSD
160	matchSEG	3887697-3914434/F	26738	3791252-3817989/F	26738	0.00	26738/0/0/0	-
161	indel	-	-	3817990-3819088/F	1099	-	-	MGE:ISPlu3+TSD
161	matchSEG	3914435-3923547/F	9113	3819089-3828201/F	9113	0.20	9113/18/0/0	-
162	indel	3923548-3924543/F	996	-	-	-	-	MGE:ISPlu6+TSD
162	matchSEG	3924544-3941300/F	16757	3828202-3844961/F	16760	0.22	16758/36/1/2	-

163	indel	3941301-3942223/F	923	-	-	-	-	MGE:ISPlu7+TSD
163	matchSEG	3942224-3948594/F	6371	3844962-3851319/F	6358	0.71	6360/43/2/11	-
164	indel	-	-	3851320-3852313/F	994	-	-	MGE:ISPlu11+TSD
164	matchSEG	3948595-3960593/F	11999	3852314-3864312/F	11999	0.02	11999/2/0/0	-
165	indel	-	-	3864313-3865398/F	1086	-	-	MGE:ISPlu1+TSD
165	matchSEG	3960594-3965592/F	4999	3865399-3870395/F	4997	1.02	4999/49/2/0	DissimLocal
166	indel	3965593-3965717/F	125	-	-	-	-	MGE:MITEPlu5+TSD
166	matchSEG	3965718-3981210/F	15493	3870396-3885889/F	15494	0.16	15494/24/1/0	-
167	indel	3981211-3982309/F	1099	-	-	-	-	MGE:ISPlu19+TSD
167	matchSEG	3982310-3984852/F	2543	3885890-3888432/F	2543	0.04	2543/1/0/0	-
168	indel	3984853-3986003/F	1151	-	-	-	-	MGE:ISPlu10+TSD
168	matchSEG	3986004-4006407/F	20404	3888433-3908836/F	20404	0.03	20404/7/0/0	-
169	indel	4006408-4085197/F	78790	-	-	-	-	specific(prophage R16)+MGE:ISPlu7+MGE:ISPlu19

169	matchSEG	4085198-4205846/F	120649	3908837-4029444/F	120608	0.23	120566/260/13/138	-
170	indel	4205847-4206945/F	1099	-	-	-	-	MGE:ISPlu19+TSD
170	matchSEG	4206946-4207003/F	58	4029445-4029502/F	58	0.00	58/0/0/0	-
171	copynr	4207004-4207549/F	546	4029503-4030123/F	621	-	-	GTTTTACAG(TT01m:46/ DJC:49)+GTTAAAACTATC(TT01m:11/ DJC:15)
171	matchSEG	4207550-4218332/F	10783	4030124-4040906/F	10783	0.00	10783/0/0/0	-
172	indel	4218333-4244937/F	26605	-	-	-	-	InternallyRepeated(PhRepA)+MGE:ISPlu9
172	matchSEG	4244938-4251874/F	6937	4040907-4047843/F	6937	1.99	6937/138/0/0	DissimLocal(PhRepA)
173	indel	4251875-4252224/F	350	-	-	-	-	specific+MGE:MITEPlu5
173	matchSEG	4252225-4260991/F	8767	4047844-4056612/F	8769	1.15	8768/100/1/1	DissimLocal
174	indel	-	-	4056613-4056737/F	125	-	-	MGE:MITEPlu5
174	matchSEG	4260992-4265840/F	4849	4056738-4061589/F	4852	3.34	4821/153/8/67	DissimGlobal
175	approxI	4265841-4266016/F	176	4061590-4061596/F	7	-	-	TT01m:specific+MGE:MITEPlu5 ; DJC:FewUnaligned
175	matchSEG	4266017-4355785/F	89769	4061597-4151386/F	89790	0.13	89770/111/2/21	-

176	indel	-	-	4151387-4153733/F	2347	-	-	MGE:ISPlu20+TSD
176	matchSEG	4355786-4357367/F	1582	4153734-4155315/F	1582	0.00	1582/0/0/0	-
177	indel	-	-	4155316-4156591/F	1276	-	-	InternallyRepeated(PhRepA) (prophage R17)
177	matchSEG	4357368-4358242/F	875	4156592-4157458/F	867	6.32	870/52/3/5	DissimButShort(PhRepA)
178	indel	-	-	4157459-4188522/F	31064	-	-	InternallyRepeated(PhRepA) (prophage R17)
178	matchSEG	4358243-4362674/F	4432	4188523-4192954/F	4432	0.45	4432/20/0/0	-
179	replace	4362675-4373635/F	10961	4192955-4195357/F	2403	-	-	TT01m:InternallyRepeated(PhRepA) ; DJC:InternallyRepeated(PhRepA)
179	matchSEG	4373636-4377315/F	3680	4195358-4199037/F	3680	0.41	3680/15/0/0	-
180	replace	4377316-4379785/F	2470	4199038-4201087/F	2050	-	-	TT01m:InternallyRepeated(PhRepA) ; DJC:InternallyRepeated(PhRepA)
180	matchSEG	4379786-4386331/F	6546	4201088-4207634/F	6547	2.76	6547/180/1/0	DissimGlobal(PhRepA)
181	indel	4386332-4387430/F	1099	-	-	-	-	MGE:ISPlu19+TSD
181	matchSEG	4387431-4394212/F	6782	4207635-4214416/F	6782	0.01	6782/1/0/0	-
182	indel	4394213-4395311/F	1099	-	-	-	-	MGE:ISPlu19+TSD

182	matchSEG	4395312-4399578/F	4267	4214417-4218683/F	4267	0.40	4267/17/0/0	-
183	indel	4399579-4401704/F	2126	-	-	-	-	specific
183	matchSEG	4401705-4402937/F	1233	4218684-4219916/F	1233	0.49	1233/6/0/0	-
184	indel	-	-	4219917-4221002/F	1086	-	-	MGE:ISPlu1+TSD
184	matchSEG	4402938-4449955/F	47018	4221003-4268009/F	47007	0.05	47008/23/1/10	-
185	indel	4449956-4450181/F	226	-	-	-	-	specific
185	matchSEG	4450182-4489799/F	39618	4268010-4307612/F	39603	0.07	39604/25/1/14	-
186	indel	-	-	4307613-4308608/F	996	-	-	MGE:ISPlu6+TSD
186	matchSEG	4489800-4493024/F	3225	4308609-4311833/F	3225	1.36	3225/44/0/0	DissimLocal
187	indel	4493025-4493125/F	101	-	-	-	-	MGE:MITEPlu5
187	matchSEG	4493126-4527061/F	33936	4311834-4345782/F	33949	0.12	33938/37/3/12	-
188	indel	-	-	4345783-4347556/F	1774	-	-	MGE:ISPlu4+TSD
188	matchSEG	4527062-4644133/F	117072	4347557-4464578/F	117022	0.14	117020/160/6/60	-

189	indel	4644134-4645232/F	1099	-	-	-	-	MGE:ISPlu3+TSD
189	matchSEG	4645233-4650992/F	5760	4464579-4470338/F	5760	0.17	5760/10/0/0	-
190	indel	-	-	4470339-4472685/F	2347	-	-	MGE:ISPlu20+TSD
190	matchSEG	4650993-4707665/F	56673	4472686-4529361/F	56676	0.17	56674/94/4/5	-
191	indel	4707666-4709269/F	1604	-	-	-	-	MGE:ISPlu14+TSD
191	matchSEG	4709270-4767693/F	58424	4529362-4587784/F	58423	0.10	58424/60/1/0	-
192	indel	4767694-4769467/F	1774	-	-	-	-	MGE:ISPlu4+TSD
192	matchSEG	4769468-4815443/F	45976	4587785-4633710/F	45926	0.06	45928/24/2/48	-
193	indel	4815444-4816440/F	997	-	-	-	-	MGE:ISPlu6+TSD
193	matchSEG	4816441-4862212/F	45772	4633711-4679510/F	45800	0.01	45773/2/1/27	-
194	indel	4862213-4864559/F	2347	-	-	-	-	MGE:ISPlu20+TSD
194	matchSEG	4864560-4919862/F	55303	4679511-4734812/F	55302	0.04	55303/22/1/0	-
195	indel	4919863-4921363/F	1501	-	-	-	-	MGE:ISPlu9+TSD

195	matchSEG	4921364-4938114/F	16751	4734813-4751572/F	16760	1.01	16752/168/1/8	DissimLocal
196	indel	-	-	4751573-4760045/F	8473	-	-	InternallyRepeated
196	matchSEG	4938115-4958917/F	20803	4760046-4780848/F	20803	0.42	20803/88/0/0	-
197	indel	-	-	4780849-4781934/F	1086	-	-	MGE:ISPlu1+TSD
197	matchSEG	4958918-4960398/F	1481	4781935-4783415/F	1481	0.20	1481/3/0/0	-
198	replace	4960399-4962789/F	2391	4783416-4783532/F	117	-	-	TT01m:InternallyRepeated+MGE:MITEPlu2 ; DJC:MGE:MITEPlu5
198	matchSEG	4962790-4996051/F	33262	4783533-4816795/F	33263	0.33	33253/107/2/21	-
199	indel	-	-	4816796-4816920/F	125	-	-	MGE:MITEPlu5+TSD
199	matchSEG	4996052-5000336/F	4285	4816921-4821205/F	4285	0.09	4285/4/0/0	-
200	indel	5000337-5001331/F	995	-	-	-	-	MGE:ISPlu11+TSD
200	matchSEG	5001332-5013171/F	11840	4821206-4833044/F	11839	0.58	11840/68/1/0	-
201	indel	5013172-5013295/F	124	-	-	-	-	MGE:MITEPlu5
201	matchSEG	5013296-5038846/F	25551	4833045-4858596/F	25552	0.37	25552/94/1/0	-

202	indel	5038847-5039842/F	996	-	-	-	-	MGE:ISPlu6+TSD
202	matchSEG	5039843-5043876/F	4034	4858597-4862661/F	4065	1.89	4031/73/3/40	DissimLocal
203	replace	5043877-5045202/F	1326	4862662-4866066/F	3405	-	-	TT01m:specific+SeqRelated ; DJC:specific+SeqRelated+MGE:ISPlu19+MGE:ISPlu7
203	matchSEG	5045203-5049836/F	4634	4866067-4870700/F	4634	0.41	4634/19/0/0	-
204	replace	5049837-5059155/F	9319	4870701-4878173/F	7473	-	-	TT01m:specific+SeqRelated+MGE:MITEPlu5 ; DJC:specific+SeqRelated+MGE:ISPlu2
204	matchSEG	5059156-5065926/F	6771	4878174-4884945/F	6772	0.09	6772/5/1/0	-
205	indel	5065927-5066922/F	996	-	-	-	-	MGE:ISPlu6+TSD
205	matchSEG	5066923-5118865/F	51943	4884946-4936898/F	51953	0.03	51945/11/2/8	-
206	approxI	5118866-5118868/F	3	4936899-4937026/F	128	-	-	TT01m:FewUnaligned ; DJC:MGE:MITEPlu5+FewAdjacent
206	matchSEG	5118869-5130720/F	11852	4937027-4948893/F	11867	0.04	11853/4/1/14	-
207	indel	-	-	4948894-4949018/F	125	-	-	specific
207	matchSEG	5130721-5140707/F	9987	4949019-4959003/F	9985	0.31	9986/30/1/1	-

208	replace	5140708-5140828/F	121	4959004-4959014/F	11	-	-	TT01m:an atypical 121 bp insert in copies C and E of 23S rRNA ; DJC:contains the typical 11 bp region found in nearly all 23S rRNAs
208	matchSEG	5140829-5153997/F	13169	4959015-4972186/F	13172	0.71	13172/90/4/1	-
209	indel	-	-	4972187-4972311/F	125	-	-	MGE:MITEPlu5+TSD
209	matchSEG	5153998-5247304/F	93307	4972312-5065630/F	93319	0.15	93297/134/2/34	-
210	indel	-	-	5065631-5065751/F	121	-	-	MGE:IRS112+TSD
210	matchSEG	5247305-5248110/F	806	5065752-5066557/F	806	0.37	806/3/0/0	-
211	indel	5248111-5249610/F	1500	-	-	-	-	MGE:ISPlu9+TSD
211	matchSEG	5249611-5270951/F	21341	5066558-5087897/F	21340	0.05	21341/10/1/0	-
212	replace	5270952-5271120/F	169	5087898-5088540/F	643	-	-	TT01m:specific ; DJC:specific
212	matchSEG	5271121-5275254/F	4134	5088541-5092674/F	4134	0.12	4134/5/0/0	-
213	indel	5275255-5276177/F	923	-	-	-	-	MGE:ISPlu7+TSD
213	matchSEG	5276178-5322300/F	46123	5092675-5138797/F	46123	0.00	46123/2/0/0	-
214	indel	5322301-5323399/F	1099	-	-	-	-	MGE:ISPlu9+TSD

214	matchSEG	5323400-5333733/F	10334	5138798-5149131/F	10334	0.01	10334/1/0/0	-
215	matchSEG	5333734-5334723/R	990	5149132-5150121/F	990	0.00	990/0/0/0	inverted between TT01m and DJC
216	matchSEG	5334724-5334801/F	78	5150122-5150199/F	78	0.00	78/0/0/0	-
217	indel	-	-	5150200-5152546/F	2347	-	-	MGE:ISPlu20+TSD
217	matchSEG	5334802-5393324/F	58523	5152547-5211078/F	58532	0.24	58520/140/3/18	-
218	indel	5393325-5393507/F	183	-	-	-	-	MGE:MITEPlu5+TSD
218	matchSEG	5393508-5416451/F	22944	5211079-5234049/F	22971	0.22	22945/48/3/28	-
219	indel	5416452-5418225/F	1774	-	-	-	-	MGE:ISPlu4+TSD
219	matchSEG	5418226-5529800/F	111575	5234050-5345614/F	111565	0.07	111568/74/4/8	-
220	replace	5529801-5531603/F	1803	5345615-5346906/F	1292	-	-	TT01m:TAAGCAAA(20),TAAGTAAA(46),MGE:ISPlu19,TAAGTAAA(22) ; DJC:TAAGCAAA(7),TAAGTAAA(14),MGE:ISPlu6,TAAGTAAA(16)
220	matchSEG	5531604-5559149/F	27546	5346907-5374457/F	27551	0.06	27548/14/3/4	-
221	indel	-	-	5374458-5375114/F	657	-	-	InternallyRepeated

221	matchSEG	5559150-5559200/F	51	5375115-5375165/F	51	0.00	51/0/0/0	-
222	indel	-	-	5375166-5375320/F	155	-	-	InternallyRepeated
222	matchSEG	5559201-5565234/F	6034	5375321-5381365/F	6045	0.65	6036/37/2/9	-
223	indel	-	-	5381366-5416822/F	35457	-	-	specific (prophageR21)
223	matchSEG	5565235-5614692/F	49458	5416823-5466192/F	49370	0.36	49373/175/5/87	-
224	indel	5614693-5615280/F	588	-	-	-	-	MGE:ISPlu2
224	matchSEG	5615281-5662045/F	46765	5466193-5512937/F	46745	0.43	46730/196/3/53	-
225	replace	5662046-5667475/F	5430	5512938-5516337/F	3400	-	-	TT01m:specific+SeqRelated+MGE:multi ; DJC:specific+SeqRelated
225	matchSEG	5667476-5687677/F	20202	5516338-5536539/F	20202	0.52	20202/105/0/0	-

Supplementary Table S3a: Mapping of gene codes between the TT01, TT01m and DJC genomes. This table, which was generated in oder to increase readability of this legend is a sample table of Supplementary table S3b.; Supplementary Table S3b, which is provided as separate file, reports the complete code mapping for protein-coding genes from the *P. luminescens* TT01 genome (*plu* codes) to the TT01m genome (PluTT01m) codes (5110 rows). For informative purposes, the codes from the TT01m genome are also mapped to those from the strain DJC genome. Placeholders (-; dash) indicate that a mapping gene is not annotated in the corresponding genome. It should be noted that missing gene calls, identified during this mapping project, have been post-predicted for the TT01m and DJC

genomes. For the TT01m genome, placeholders are only used when an ORF from the TT01 genome could not be mapped for the reason indicated (see below). Genes from the DJC genome without mapping to the TT01m genome are not listed. Supplementary Table S3a is sorted according to the information flow of this legend to support readability. Supplementary table S3b is sorted along the TT01m genome (indicated by increasing serial numbers) with intercalation of unmapped TT01 ORFs at their genome position. It should be noted that TT01m codes ending with 0 or 5 refer to genes originally annotated by the PGAP pipeline, all other TT01m genes have been post-predicted upon manual curation. The first 13 lines are regular genes with a 1:1:1 mapping between the TT01m, TT01 and DJC genomes. A placeholder in the comment field indicates that there is full annotation consistency between TT01m and TT01; accordingly, the protein sequences are identical. The following special cases were encountered: (a) a gene exists only in the TT01m and TT01 genome but not in the DJC genome; (b) a start codon assignment discrepancy may be encountered between the TT01m and TT01 genomes (comment term: StartDiscrep); (c) the inability to map a TT01m gene to a TT01 gene may be due to a missing gene call (comment term: missingGeneCall); (d) a disrupted gene, which has been called in the TT01m genome may not have been called in the TT01 genome (comment term: pseudoNotCalled); (e,f) automatic gene mapping depends on the positional correlation of the stop codon, which is ensured only for regular genes. Disrupted genes that do not extend up to the stop codon depend on manual mapping; (e) the gene may be disrupted but is stated to be a regular gene in the TT01 genome (comment term: isPseudo); (f) the gene may be regular but is invalidly annotated as disrupted in the TT01 genome (comment term: noPseudo); (g) some gene calls by the PGAP pipeline are ORFs which are considered not to code for a protein (spurious ORF). This is indicated only if the corresponding ORF has not been called for the TT01 genome (comment term: spurious); (h) a special case are "pseudocoding frames", which are gene calls traversing or covering the short (123 bp) mobile genetic element MITEPlu5, but with only short extensions into the regular genome (comment term: pseudoCoding); (i) if a pseudocoding frame has been called for both, the TT01m and TT01 genomes, the start codon assignment may be disrepanct (comment term: StartDiscrep_pseudocoding) (j,k,l) all gene calls in the TT01 genome which cannot be mapped to the TT01m genome have been evaluated and genes were post-predicted for the TT01m genome. Exceptions are (j) TT01 ORFs which are rated to be spurious (comment term: spurious); (k) short remnants of disrupted genes, typically with a homology region of less than 50 codons

(comment term: pseudoSkipped); (l) pseudocoding frames annotated only in the TT01 genome; (m) in some cases, a single TT01m gene maps to more than one TT01 gene, indicated by multiple TT01 codes (comment term: multiTT01). Typical cases are disrupted genes where the N-terminal partial coding region is called as one gene and a C-terminal partial coding region is called as an independent gene which an internal ATG codon mis-assigned to be a start codon. It should be noted that the underlying genome sequence is identical between the TT01m and TT01 genomes for the cases marked by this flag; (n) in some cases, a single TT01m gene is mapped to more than one DJC gene; in such cases, the DJC gene has been disrupted and has been targeted by a mobile genetic element; (o) similarly, if the TT01m gene has been targeted by a mobile genetic element, a single DJC gene maps to more than one TT01m gene. Because this table is based on TT01m genes, this situation is indicated by adding "(part)" after the DJC code; (p,q) gene disruption may generate chimeric genes; (p) these are listed only if discrepantly annotated in the TT01m and TT01 genomes (comment term: chimericPseudo); (q) this complication may be combined with mapping of a single TT01m gene to multiple TT01 genes (comment term: multiTT01+chimericPseudo); (r,s,t,u,v,w,x) while all the above refer to genes encoded on regions of sequence identity between the TT01m and TT01 genomes, the genome differences (see Supplementary Table S1) lead to gene differences; (r) some genes were not automatically mapped because they are encoded on an inverted genome region (comment term: onInversion); (s) genes encoded on an insertion in TT01m cannot be mapped (comment term: TT01m_insertion); (t) likewise, genes encoded on an insertion in TT01 cannot be mapped (comment term: TT01_insertion); (u) some genes carry a point mutation (comment term: e.g. *plu0049*(G177D)); (v) in case of a frameshift, a single TT01m gene may map to more than one TT01 gene, indicated by multiple TT01 codes (comment term: e.g. splitORFs(*plu1144*+*plu1145*)); (w) a frameshift may lead to an aberrant terminal sequence (comment term: e.g. aberrantN(plu2762)); (x) genes may be affected by more than one genome difference (comment term: e.g. severalDiffs(*plu1426*); (y,z) the genome inversions between TT01m and TT01 may affect protein coding genes traversing the junction; (y) in one scenario, automatic mapping failed but manual mapping shows that the encoded proteins are identical, probably due to the sequence duplication outside the inversion (comment term: [seqid;traverseJunction]); (z) in one case, the inversion resulted in gene truncation (comment term: traversesInversionJunction+truncatedAtN(PluTT01m_15460)); (Aa) an inserted sequence may affect a gene encoded across the

integration point (comment term: divergentN_byTT01m_insertion); (Ab) one disrupted gene could not be automatically mapped because a CTG codon had been mis-assigned to be a start codon (translated as Met) in the TT01 genome (comment term: *plu4790*(CTGcodon_notStart)). This sample tables lists all of the exceptions and all combinations thereof. The complete Supplementary table S3b is provided in two formats: as a space-separated list (*.txt) and in Excel format (*.xlsx).

TT01m code	TT01 code	DJC code	comment
PluTT01m_00005	*plu0001*	PluDJC_00005	-
PluTT01m_00010	*plu0002*	PluDJC_00010	-
PluTT01m_00015	*plu0003*	PluDJC_00015	-
PluTT01m_00020	*plu0004*	PluDJC_00020	-
PluTT01m_00025	*plu0005*	PluDJC_00025	-
PluTT01m_00035	*plu0006*	PluDJC_00040	-
PluTT01m_00045	*plu0008*	PluDJC_00050	-
PluTT01m_00050	*plu0009*	PluDJC_00055	-
PluTT01m_00055	*plu0010*	PluDJC_00060	-
PluTT01m_00060	*plu0011*	PluDJC_00065	-
PluTT01m_00070	*plu0013*	PluDJC_00075	-
PluTT01m_00075	*plu0014*	PluDJC_00080	-
PluTT01m_00080	*plu0015*	PluDJC_00085	-
PluTT01m_01630	*plu0319*	-	-
PluTT01m_00040	*plu0007*	PluDJC_00045	StartDiscrep
PluTT01m_01740	*plu0340*	-	StartDiscrep
PluTT01m_00100	-	PluDJC_00105	missingGeneCall
PluTT01m_00030	-	-	missingGeneCall
PluTT01m_00027	-	PluDJC_00027	pseudoNotCalled
PluTT01m_03705	-	-	pseudoNotCalled
PluTT01m_00665	*plu0128*	PluDJC_00720	isPseudo

PluTT01m_01725	*plu0337*	-	isPseudo
PluTT01m_00310	*plu0062*	PluDJC_00315	noPseudo
PluTT01m_01390	-	PluDJC_01440	spurious
PluTT01m_01425	-	-	spurious
PluTT01m_00835	-	PluDJC_00890	pseudoCoding
PluTT01m_11435	-	-	pseudoCoding
PluTT01m_06915	*plu1346*	PluDJC_06495	StartDiscrep_pseudocoding
-	*plu0016*	-	spurious
-	*plu0083*	-	pseudoSkipped
-	*plu0725*	-	pseudoCoding
PluTT01m_00355	*plu0066*+*plu0067*	PluDJC_00360	multiTT01
PluTT01m_05515	*plu1076*+*plu1077*	-	multiTT01
PluTT01m_00785	*plu0153*	PluDJC_00840+PluDJC_00845	-
PluTT01m_00600	*plu0116*	PluDJC_00650+PluDJC_00655	StartDiscrep
PluTT01m_02765	-	PluDJC_02672+PluDJC_02677	missingGeneCall
PluTT01m_12025	*plu2334*	PluDJC_12365+PluDJC_12370	noPseudo
PluTT01m_15170	*plu2953*	PluDJC_15080(part)	StartDiscrep
PluTT01m_04385	*plu0853*	PluDJC_04355(part)	isPseudo
PluTT01m_16775	-	PluDJC_16650(part)	pseudoNotCalled
PluTT01m_05615	*plu1101*	-	chimericPseudo
PluTT01m_05620	*plu1102*+*plu1101*(N-term)	-	multiTT01+chimericPseudo
PluTT01m_00165	*plu0032*	PluDJC_00165	onInversion
PluTT01m_15470	*plu3011*	-	onInversion
PluTT01m_00155	*plu0034*	PluDJC_00175	onInversion+StartDiscrep
PluTT01m_15465	*plu3012*	-	onInversion+StartDiscrep
PluTT01m_18435	-	-	TT01m_insertion
-	*plu3696*	-	TT01_insertion
PluTT01m_00245	*plu0049*	PluDJC_00250	*plu0049*(G177D)
PluTT01m_01370	*plu0267*	PluDJC_01420	*plu0267*(N134K)

PluTT01m_02545	*plu0497*	PluDJC_02460	*plu0497*(K118E)
PluTT01m_07300	*plu1414*	PluDJC_06915	StartDiscrep+plu1414(S191C)
PluTT01m_12570	*plu2443*	PluDJC_12895	*plu2443*(W139S)
PluTT01m_21290	*plu4163*	-	*plu4163*(H253Q)
PluTT01m_05840	*plu1144+plu1145*	-	splitORFs(plu1144+plu1145)
PluTT01m_11330	*plu2195+plu2196*	-	splitORFs(plu2195+plu2196)
PluTT01m_14175	*plu2762*	PluDJC_14485	aberrantN(plu2762)
PluTT01m_23445	*plu4585*	PluDJC_22725	aberrantC(plu4585)
PluTT01m_07370	*plu1426*	PluDJC_06985	severalDiffs(plu1426)
PluTT01m_23020	*plu4505*	PluDJC_22300	severalDiffs(plu4505)
PluTT01m_18475	*plu3594*	PluDJC_17655	[seqid;traverseJunction]
PluTT01m_15460	*plu3013*	-	traversesInversionJunction+ truncatedAtN(PluTT01m_15460)
PluTT01m_18430	*plu3593*	-	divergentN_byTT01m_insertion
PluTT01m_24585	*plu4790*	PluDJC_23855	*plu4790*(CTGcodon_notStart)

Supplementary Table S4: Strain-specific protein-coding genes. In the 1st part, genes specific for *P. luminescens* strain DJC are listed, in the 2nd part those for strain TT01m. Proteins are sorted by protein name, except that those annotated as "hypothetical protein" have been moved to the end of the list. If assigned, gene names are given in parenthesis, preceding the protein name. For convenience, the codes of the corresponding ORFs and therefore the respective proteins in the published TT01 genome (Plu numbers) are provided for TT01m-specific protein-coding genes/proteins. No such mapping is possible for DJC-specific protein-coding genes (indicated by a dash).

**strain-specific proteins in *P. luminescens* DJC**		
**code**	**TT01 equivalent**	**protein name/specificity**
PluDJC_08165	-	DDE endonuclease
PluDJC_09225	-	DDE endonuclease
PluDJC_10625	-	DNA adenine methylase
PluDJC_24075	-	DNA adenine methylase
PluDJC_08030	-	DNA circulation protein
PluDJC_09385	-	DNA circulation protein
PluDJC_10620	-	DNA cytosine methyltransferase
PluDJC_07945	-	DNA primase
PluDJC_08160	-	DNA transposition protein
PluDJC_09230	-	DNA transposition protein
PluDJC_08170	-	DNA-binding protein
PluDJC_09220	-	DNA-binding protein
PluDJC_10590	-	DNA-binding protein
PluDJC_09430	-	DNA-invertase
PluDJC_21410	-	DUF1837 domain-containing protein
PluDJC_08055	-	DUF2635 domain-containing protein
PluDJC_09360	-	DUF2635 domain-containing protein
PluDJC_08085	-	DUF2730 domain-containing protein
PluDJC_09300	-	DUF2730 domain-containing protein
PluDJC_24085	-	DUF2732 domain-containing protein
PluDJC_08145	-	DUF3164 domain-containing protein
PluDJC_09245	-	DUF3164 domain-containing protein
PluDJC_08220	-	HNH endonuclease
PluDJC_07955	-	XRE family transcriptional regulator
PluDJC_10580	-	XRE family transcriptional regulator
PluDJC_24105	-	XRE family transcriptional regulator
PluDJC_03685	-	(higA) addiction module antidote protein, HigA family
PluDJC_07950	-	antitermination protein
PluDJC_06610	-	colicin immunity protein
PluDJC_11810	-	growth inhibitor PemK
PluDJC_07940	-	helicase
PluDJC_21420	-	helicase

PluDJC_10565	-	integrase
PluDJC_23915	-	late control protein D
PluDJC_11755	-	methionine repressor-like protein
PluDJC_10670	-	oxidoreductase
PluDJC_24035	-	oxidoreductase
PluDJC_08110	-	peptidoglycan-binding protein
PluDJC_09280	-	peptidoglycan-binding protein
PluDJC_10635	-	replication endonuclease
PluDJC_21380	-	restriction endonuclease subunit S
PluDJC_07925	-	transcriptional regulator
PluDJC_08115	-	transcriptional regulator
PluDJC_09275	-	transcriptional regulator
PluDJC_15135	-	transcriptional regulator
PluDJC_23910	-	transcriptional regulator
PluDJC_11985	-	type II toxin-antitoxin system Phd/YefM family antitoxin
PluDJC_11990	-	type II toxin-antitoxin system RelE/ParE family toxin
PluDJC_10660	-	type II toxin-antitoxin system YafO family toxin
PluDJC_11485	-	type VI secretion system tube protein Hcp
PluDJC_08090	-	zinc finger domain protein
PluDJC_00035	-	hypothetical protein
PluDJC_00550	-	hypothetical protein
PluDJC_00555	-	hypothetical protein
PluDJC_00560	-	hypothetical protein
PluDJC_00570	-	hypothetical protein
PluDJC_00580	-	hypothetical protein
PluDJC_00585	-	hypothetical protein
PluDJC_02660	-	hypothetical protein
PluDJC_03695	-	hypothetical protein
PluDJC_05100	-	hypothetical protein
PluDJC_07325	-	hypothetical protein
PluDJC_07920	-	hypothetical protein
PluDJC_07935	-	hypothetical protein
PluDJC_07960	-	hypothetical protein
PluDJC_07965	-	hypothetical protein
PluDJC_07970	-	hypothetical protein
PluDJC_07975	-	hypothetical protein
PluDJC_08000	-	hypothetical protein
PluDJC_08005	-	hypothetical protein
PluDJC_08010	-	hypothetical protein
PluDJC_08015	-	hypothetical protein
PluDJC_08035	-	hypothetical protein
PluDJC_08040	-	hypothetical protein
PluDJC_08065	-	hypothetical protein
PluDJC_08070	-	hypothetical protein
PluDJC_08095	-	hypothetical protein
PluDJC_08100	-	hypothetical protein

PluDJC_08105	-	hypothetical protein
PluDJC_08120	-	hypothetical protein
PluDJC_08125	-	hypothetical protein
PluDJC_08130	-	hypothetical protein
PluDJC_08135	-	hypothetical protein
PluDJC_08140	-	hypothetical protein
PluDJC_08150	-	hypothetical protein
PluDJC_08155	-	hypothetical protein
PluDJC_08175	-	hypothetical protein
PluDJC_08200	-	hypothetical protein
PluDJC_08205	-	hypothetical protein
PluDJC_08210	-	hypothetical protein
PluDJC_08215	-	hypothetical protein
PluDJC_08225	-	hypothetical protein
PluDJC_08265	-	hypothetical protein
PluDJC_08270	-	hypothetical protein
PluDJC_08285	-	hypothetical protein
PluDJC_08310	-	hypothetical protein
PluDJC_08330	-	hypothetical protein
PluDJC_09215	-	hypothetical protein
PluDJC_09235	-	hypothetical protein
PluDJC_09240	-	hypothetical protein
PluDJC_09250	-	hypothetical protein
PluDJC_09255	-	hypothetical protein
PluDJC_09260	-	hypothetical protein
PluDJC_09265	-	hypothetical protein
PluDJC_09270	-	hypothetical protein
PluDJC_09285	-	hypothetical protein
PluDJC_09295	-	hypothetical protein
PluDJC_09310	-	hypothetical protein
PluDJC_09315	-	hypothetical protein
PluDJC_09320	-	hypothetical protein
PluDJC_09345	-	hypothetical protein
PluDJC_09350	-	hypothetical protein
PluDJC_09355	-	hypothetical protein
PluDJC_09375	-	hypothetical protein
PluDJC_09380	-	hypothetical protein
PluDJC_09400	-	hypothetical protein
PluDJC_09405	-	hypothetical protein
PluDJC_09410	-	hypothetical protein
PluDJC_09415	-	hypothetical protein
PluDJC_10570	-	hypothetical protein
PluDJC_10575	-	hypothetical protein
PluDJC_10585	-	hypothetical protein
PluDJC_10595	-	hypothetical protein
PluDJC_10600	-	hypothetical protein
PluDJC_10605	-	hypothetical protein

PluDJC_10610	-	hypothetical protein
PluDJC_10615	-	hypothetical protein
PluDJC_10630	-	hypothetical protein
PluDJC_10640	-	hypothetical protein
PluDJC_10645	-	hypothetical protein
PluDJC_10650	-	hypothetical protein
PluDJC_10655	-	hypothetical protein
PluDJC_10755	-	hypothetical protein
PluDJC_10790	-	hypothetical protein
PluDJC_10795	-	hypothetical protein
PluDJC_10800	-	hypothetical protein
PluDJC_11765	-	hypothetical protein
PluDJC_11820	-	hypothetical protein
PluDJC_11980	-	hypothetical protein
PluDJC_11995	-	hypothetical protein
PluDJC_15130	-	hypothetical protein
PluDJC_15370	-	hypothetical protein
PluDJC_16590	-	hypothetical protein
PluDJC_20855	-	hypothetical protein
PluDJC_23875	-	hypothetical protein
PluDJC_23880	-	hypothetical protein
PluDJC_23885	-	hypothetical protein
PluDJC_23995	-	hypothetical protein
PluDJC_24045	-	hypothetical protein
PluDJC_24055	-	hypothetical protein
PluDJC_24060	-	hypothetical protein
PluDJC_24080	-	hypothetical protein
PluDJC_24090	-	hypothetical protein
PluDJC_24095	-	hypothetical protein
PluDJC_24100	-	hypothetical protein
**strain-specific proteins in *P. luminescens* TT01m**		
**code**	**TT01 equivalent**	**protein name/specificity**
PluTT01m_09610	Plu1869	3-isopropylmalate dehydratase large subunit
PluTT01m_12700	Plu2470	ABC transporter permease
PluTT01m_14980	Plu2917	ATP-dependent helicase
PluTT01m_09630	Plu1873	ATP-grasp domain-containing protein
PluTT01m_09635	Plu1874	ATP-grasp domain-containing protein
PluTT01m_11335	Plu2197	ATP-grasp domain-containing protein
PluTT01m_04775	Plu0928	AbrB/MazE/SpoVT family DNA-binding domain-containing protein
PluTT01m_14805	Plu2885	Arc family DNA-binding protein
PluTT01m_01730	Plu0338	DNA cytosine methyltransferase
PluTT01m_15115	Plu2942	DNA methyltransferase
PluTT01m_14975	Plu2916	DNA primase
PluTT01m_17850	Plu3472	DNA replication protein DnaC
PluTT01m_17805	Plu3461	DNA-binding protein
PluTT01m_05510	Plu1075	DUF1016 domain-containing protein

PluTT01m_15080	Plu2937	DUF1317 domain-containing protein
PluTT01m_17675	Plu3435	DUF1320 domain-containing protein
PluTT01m_17830	Plu3468	DUF1367 domain-containing protein
PluTT01m_01800	Plu0354	DUF1795 domain-containing protein
PluTT01m_14855	Plu2896	DUF2184 domain-containing protein
PluTT01m_17495	Plu3401	DUF2184 domain-containing protein
PluTT01m_17680	Plu3436	DUF2190 domain-containing protein
PluTT01m_14865	Plu2898	DUF2213 domain-containing protein
PluTT01m_17505	Plu3403	DUF2213 domain-containing protein
PluTT01m_02730	Plu0535	DUF2247 domain-containing protein
PluTT01m_07715	Plu1482	DUF2384 domain-containing protein
PluTT01m_14950	Plu2912	DUF2591 domain-containing protein
PluTT01m_14760	Plu2875	DUF2612 domain-containing protein
PluTT01m_17405	Plu3383	DUF2612 domain-containing protein
PluTT01m_17780	Plu3456	DUF3102 domain-containing protein
PluTT01m_14825	plu2890	DUF3277 domain-containing protein
PluTT01m_14830	Plu2891	DUF3383 domain-containing protein
PluTT01m_17465	Plu3395	DUF3383 domain-containing protein
PluTT01m_17715	Plu3443	DUF3486 domain-containing protein
PluTT01m_22110	Plu4326	DUF3732 domain-containing protein
PluTT01m_14850	Plu2895	DUF4054 domain-containing protein
PluTT01m_17485	Plu3399	DUF4054 domain-containing protein
PluTT01m_04435	Plu0863	DUF4065 domain-containing protein
PluTT01m_16715	Plu3245	DUF4123 domain-containing protein
PluTT01m_09460	Plu1836	DUF4297 domain-containing protein
PluTT01m_17540	Plu3411	DUF4868 domain-containing protein
PluTT01m_17705	Plu3441	DUF935 domain-containing protein
PluTT01m_12725	Plu2474	GTP cyclohydrolase II RibA
PluTT01m_17775	Plu3455	IclR family transcriptional regulator
PluTT01m_09600	Plu1886	LLM class flavin-dependent oxidoreductase
PluTT01m_07880	Plu1514	MFS transporter
PluTT01m_09640	Plu1875	MFS transporter
PluTT01m_07885	Plu1515	MccB protein
PluTT01m_09625	Plu1872	N-acetyltransferase
PluTT01m_13600	Plu2650	N-acetyltransferase
PluTT01m_04780	Plu0929	PIN domain-containing protein
PluTT01m_05630	Plu1105	PLP-dependent aminotransferase family protein
PluTT01m_01750	Plu0342	SMI1/KNR4 family protein
PluTT01m_01775	Plu0348	SMI1/KNR4 family protein
PluTT01m_05530	Plu1080	TIGR03745 family integrating conjugative element membrane protein
PluTT01m_05540	none	TIGR03746 family integrating conjugative element protein
PluTT01m_05505	Plu1074	TIGR03747 family integrating conjugative element membrane protein
PluTT01m_05545	Plu1082	TIGR03749 family integrating conjugative element protein
PluTT01m_05535	Plu1081	TIGR03750 family conjugal transfer protein

PluTT01m_05555	Plu1084	TIGR03751 family conjugal transfer lipoprotein
PluTT01m_05550	Plu1083	TIGR03752 family integrating conjugative element protein
PluTT01m_05570	Plu1087	TIGR03756 family integrating conjugative element protein
PluTT01m_05565	Plu1086	TIGR03757 family integrating conjugative element protein
PluTT01m_05525	Plu1079	TIGR03758 family integrating conjugative element protein
PluTT01m_12730	Plu2475	WD40 repeat domain-containing protein
PluTT01m_05600	Plu1095	XRE family transcriptional regulator
PluTT01m_15000	Plu2921	XRE family transcriptional regulator
PluTT01m_15005	Plu2922	XRE family transcriptional regulator
PluTT01m_17385	Plu3379	XRE family transcriptional regulator
PluTT01m_17575	Plu3418	XRE family transcriptional regulator
PluTT01m_17580	Plu3419	XRE family transcriptional regulator
PluTT01m_17765	Plu3453	XRE family transcriptional regulator
PluTT01m_11690	Plu2266	alpha/beta hydrolase
PluTT01m_11350	Plu2200	amidase
PluTT01m_14940	Plu2911	antitermination protein
PluTT01m_11425	Plu2215	class I SAM-dependent methyltransferase
PluTT01m_09445	Plu1831	conjugal transfer TraD family protein
PluTT01m_05585	Plu1090	conjugal transfer protein TraG
PluTT01m_05560	Plu1085	conjugative transfer ATPase
PluTT01m_17905	Plu3486	excisionase
PluTT01m_15075	Plu2936	exonuclease
PluTT01m_15480	Plu3010	glycosyltransferase family 1 protein
PluTT01m_05625	Plu1104	glyoxalase
PluTT01m_05660	Plu1111	(ltrA) group II intron reverse transcriptase/maturase
PluTT01m_17855	Plu3473	helix-turn-helix domain-containing protein
PluTT01m_17785	Plu3457	integrase
PluTT01m_05575	Plu1088	integrating conjugative element protein
PluTT01m_05520	Plu1078	integrative conjugative element protein, RAQPRD family
PluTT01m_17555	Plu3414	lysozyme
PluTT01m_17735	Plu3447	lytic transglycosylase domain-containing protein
PluTT01m_05635	Plu1106	mandelate dehydrogenase
PluTT01m_17425	Plu3388	oxidoreductase
PluTT01m_14800	Plu2883	phage repressor protein
PluTT01m_17760	Plu3452	prepilin peptidase
PluTT01m_15145	Plu2947	recombinase
PluTT01m_15070	Plu2935	recombinase RecT
PluTT01m_17845	Plu3471	replicative DNA helicase
PluTT01m_14970	none	restriction alleviation protein, Lar family
PluTT01m_17745	Plu3449	restriction endonuclease subunit M
PluTT01m_12735	Plu2476	serine/threonine protein kinase
PluTT01m_17865	Plu3475	tRNA-(guanine-N1)-methyltransferase
PluTT01m_17450	none	transcription elongation factor GreA
PluTT01m_14985	Plu2918	transcriptional regulator

PluTT01m_15030	Plu2928	transcriptional regulator
PluTT01m_17770	Plu3454	transcriptional regulator
PluTT01m_19140	Plu3733	transcriptional regulator
PluTT01m_17390	Plu3380	type I toxin-antitoxin system SymE family toxin
PluTT01m_01805	Plu0355	type VI secretion system tip protein VgrG
PluTT01m_01735	Plu0339	very short patch repair endonuclease
PluTT01m_00030	none	hypothetical protein
PluTT01m_01740	Plu0340	hypothetical protein
PluTT01m_01745	Plu0341	hypothetical protein
PluTT01m_01770	Plu0347	hypothetical protein
PluTT01m_01785	Plu0351	hypothetical protein
PluTT01m_01790	Plu0352	hypothetical protein
PluTT01m_02740	Plu0538	hypothetical protein
PluTT01m_02750	Plu0540	hypothetical protein
PluTT01m_02755	Plu0541	hypothetical protein
PluTT01m_05120	Plu0994	hypothetical protein
PluTT01m_05580	Plu1089	hypothetical protein
PluTT01m_05590	Plu1091	hypothetical protein
PluTT01m_05595	Plu1092	hypothetical protein
PluTT01m_05597	Plu1093	hypothetical protein
PluTT01m_05610	Plu1097	hypothetical protein
PluTT01m_05645	Plu1108+Plu1109	hypothetical protein
PluTT01m_05655	none	hypothetical protein
PluTT01m_05715	Plu1122	hypothetical protein
PluTT01m_05720	Plu1123	hypothetical protein
PluTT01m_07720	Plu1483	hypothetical protein
PluTT01m_09440	Plu1830	hypothetical protein
PluTT01m_09450	Plu1833	hypothetical protein
PluTT01m_09455	Plu1834	hypothetical protein
PluTT01m_09615	Plu1870	hypothetical protein
PluTT01m_09645	Plu1876	hypothetical protein
PluTT01m_11315	Plu2192	hypothetical protein
PluTT01m_11330	Plu2195+plu2196	hypothetical protein
PluTT01m_11420	Plu2214	hypothetical protein
PluTT01m_11430	Plu2216	hypothetical protein
PluTT01m_11635	Plu2256	hypothetical protein
PluTT01m_11640	Plu2257	hypothetical protein
PluTT01m_12205	Plu2373	hypothetical protein
PluTT01m_12690	Plu2468	hypothetical protein
PluTT01m_14765	Plu2876	hypothetical protein
PluTT01m_14770	Plu2877	hypothetical protein
PluTT01m_14780	Plu2879	hypothetical protein
PluTT01m_14785	Plu2880	hypothetical protein
PluTT01m_14790	Plu2881	hypothetical protein
PluTT01m_14802	Plu2884	hypothetical protein
PluTT01m_14810	Plu2886	hypothetical protein
PluTT01m_14815	Plu2887	hypothetical protein

PluTT01m_14820	Plu2889	hypothetical protein
PluTT01m_14835	Plu2892	hypothetical protein
PluTT01m_14840	Plu2893	hypothetical protein
PluTT01m_14845	Plu2894	hypothetical protein
PluTT01m_14860	Plu2897	hypothetical protein
PluTT01m_14870	Plu2899	hypothetical protein
PluTT01m_14875	Plu2900	hypothetical protein
PluTT01m_14885	Plu2902	hypothetical protein
PluTT01m_14890	Plu2903	hypothetical protein
PluTT01m_14895	none	hypothetical protein
PluTT01m_14900	Plu2904	hypothetical protein
PluTT01m_14925	Plu2909	hypothetical protein
PluTT01m_14930	none	hypothetical protein
PluTT01m_14935	Plu2910	hypothetical protein
PluTT01m_14945	none	hypothetical protein
PluTT01m_14955	Plu2913	hypothetical protein
PluTT01m_14960	Plu2914	hypothetical protein
PluTT01m_14965	none	hypothetical protein
PluTT01m_14995	Plu2920	hypothetical protein
PluTT01m_15010	Plu2923	hypothetical protein
PluTT01m_15015	Plu2924	hypothetical protein
PluTT01m_15020	Plu2926	hypothetical protein
PluTT01m_15025	Plu2927	hypothetical protein
PluTT01m_15035	Plu2929	hypothetical protein
PluTT01m_15040	Plu2930	hypothetical protein
PluTT01m_15045	Plu2931	hypothetical protein
PluTT01m_15050	Plu2932	hypothetical protein
PluTT01m_15055	Plu2933	hypothetical protein
PluTT01m_15060	none	hypothetical protein
PluTT01m_15065	Plu2934	hypothetical protein
PluTT01m_15085	Plu2938	hypothetical protein
PluTT01m_15090	Plu2939	hypothetical protein
PluTT01m_15095	Plu2940	hypothetical protein
PluTT01m_15100	none	hypothetical protein
PluTT01m_15105	Plu2941	hypothetical protein
PluTT01m_15110	none	hypothetical protein
PluTT01m_15120	Plu2943	hypothetical protein
PluTT01m_15125	Plu2944	hypothetical protein
PluTT01m_15130	none	hypothetical protein
PluTT01m_15135	Plu2945	hypothetical protein
PluTT01m_15140	Plu2946	hypothetical protein
PluTT01m_15470	Plu3011	hypothetical protein
PluTT01m_15490	Plu3008	hypothetical protein
PluTT01m_17400	Plu3382	hypothetical protein
PluTT01m_17410	Plu3384	hypothetical protein
PluTT01m_17415	Plu3385	hypothetical protein
PluTT01m_17417	Plu3386	hypothetical protein

PluTT01m_17420	Plu3387	hypothetical protein
PluTT01m_17430	Plu3389	hypothetical protein
PluTT01m_17435	Plu3390	hypothetical protein
PluTT01m_17440	Plu3391	hypothetical protein
PluTT01m_17445	Plu3392	hypothetical protein
PluTT01m_17455	Plu3393	hypothetical protein
PluTT01m_17460	Plu3394	hypothetical protein
PluTT01m_17470	Plu3396	hypothetical protein
PluTT01m_17475	Plu3397	hypothetical protein
PluTT01m_17480	Plu3398	hypothetical protein
PluTT01m_17490	Plu3400	hypothetical protein
PluTT01m_17500	Plu3402	hypothetical protein
PluTT01m_17515	Plu3405	hypothetical protein
PluTT01m_17525	Plu3407	hypothetical protein
PluTT01m_17530	Plu3408	hypothetical protein
PluTT01m_17545	Plu3412	hypothetical protein
PluTT01m_17565	Plu3416	hypothetical protein
PluTT01m_17625	none	hypothetical protein
PluTT01m_17630	Plu3428	hypothetical protein
PluTT01m_17650	none	hypothetical protein
PluTT01m_17665	Plu3433	hypothetical protein
PluTT01m_17670	Plu3434	hypothetical protein
PluTT01m_17685	Plu3437	hypothetical protein
PluTT01m_17710	Plu3442	hypothetical protein
PluTT01m_17720	Plu3444	hypothetical protein
PluTT01m_17725	Plu3445	hypothetical protein
PluTT01m_17730	Plu3446	hypothetical protein
PluTT01m_17740	Plu3448	hypothetical protein
PluTT01m_17750	Plu3450	hypothetical protein
PluTT01m_17755	Plu3451	hypothetical protein
PluTT01m_17790	Plu3458	hypothetical protein
PluTT01m_17795	Plu3459	hypothetical protein
PluTT01m_17815	Plu3463	hypothetical protein
PluTT01m_17835	Plu3469	hypothetical protein
PluTT01m_17860	Plu3474	hypothetical protein
PluTT01m_17870	Plu3476	hypothetical protein
PluTT01m_17875	Plu3477	hypothetical protein
PluTT01m_17880	Plu3479	hypothetical protein
PluTT01m_17890	Plu3482	hypothetical protein
PluTT01m_17895	Plu3483	hypothetical protein
PluTT01m_17900	Plu3484	hypothetical protein
PluTT01m_17902	Plu3485	hypothetical protein
PluTT01m_21670	Plu4240	hypothetical protein
PluTT01m_22095	Plu4324	hypothetical protein
PluTT01m_22115	Plu4327	hypothetical protein
PluTT01m_22120	Plu4328	hypothetical protein
PluTT01m_22130	Plu4330	hypothetical protein

Supplementary Table S5: Genes which are disrupted in only one *P. luminescens* strain. In the 1st part, genes disrupted only in strain DJC are listed, in the 2nd part those disrupted only in strain TT01m. Proteins are sorted by code and thus along the genome. The disruption type is either targeted or pseudo. Type pseudo may have resulted from an in-frame stop codon, a frameshift, or gene truncation at one of its ends. Targeted means that the gene has suffered integration of a transposon or a MITE so that the coding region becomes non-contiguous. The fragments of the Cas3f protein, which has probably been targeted by a prophage (see Supplementary table S5), are shown in consecutive lines. In PGAP annotation, the fragments of a targeted gene are annotated as independent ORFs, each with its own code. For convenience, the codes of the corresponding ORFs in the published TT01 genome (*plu* numbers) are provided.

	**genes with are disrupted only in *P. luminescens* strain DJC**			
**regular gene**	**disrupted gene**	**TT01 equivalent**	**type**	**protein name/function**
PluTT01m_09245	PluDJC_09435	*plu1791*	pseudo	(cas3f) type I-F CRISPR-associated helicase Cas3
PluTT01m_09245	PluDJC_09210	*plu1791*	pseudo	(cas3f) type I-F CRISPR-associated helicase Cas3
PluTT01m_11310	PluDJC_11685	*plu2191*	pseudo	(ectB) diaminobutyrate--2-oxoglutarate transaminase
PluTT01m_11700	PluDJC_12045	*plu2268*	pseudo	DNA-binding protein
PluTT01m_11645	PluDJC_11997	none	pseudo	DUF4184 domain-containing protein
PluTT01m_17090	PluDJC_16945+PluDJC_16935	*plu3324*	targeted	MARTX multifunctional-autoprocessing repeats-in-toxin holotoxin RtxA
PluTT01m_02890	PluDJC_02820+PluDJC_02830	*plu0567*	targeted	MFS transporter
PluTT01m_11345	PluDJC_11703	*plu2199*	pseudo	MFS transporter
PluTT01m_11320	PluDJC_11690	*plu2193*	pseudo	TauD/TfdA family dioxygenase
PluTT01m_04120	PluDJC_04090	*plu0801*	pseudo	UPF0261 family protein
PluTT01m_13885	PluDJC_14195+PluDJC_14200	*plu2707*	targeted	XRE family transcriptional regulator
PluTT01m_04770	PluDJC_04770+PluDJC_04750	*plu0927*	targeted	MFS transporter
PluTT01m_19430	PluDJC_18720	*plu3788*	pseudo	cysteine protease
PluTT01m_15520	PluDJC_15410	*plu3018*	pseudo	glycosyltransferase family 2 protein
PluTT01m_00785	PluDJC_00840+PluDJC_00845	*plu0153*	targeted	helicase
PluTT01m_19150	PluDJC_18430+PluDJC_18440	*plu3735*	targeted	helix-turn-helix transcriptional regulator
PluTT01m_12615	PluDJC_12940+PluDJC_12950	*plu2453*	targeted	hemagglutinin
PluTT01m_00600	PluDJC_00650+PluDJC_00655	*plu0116*	targeted	hypothetical protein

PluTT01m_12025	PluDJC_12365+PluDJC_12370	*plu2334*	targeted	hypothetical protein
PluTT01m_13610	PluDJC_13915+PluDJC_13905	*plu2652*	targeted	hypothetical protein
PluTT01m_19765	PluDJC_19070+PluDJC_19060	*plu3854*	targeted	hypothetical protein
PluTT01m_21825	PluDJC_21130+PluDJC_21120	*plu4270*	targeted	nucleotidyl transferase AbiEii/AbiGii toxin family protein
PluTT01m_22190	PluDJC_21485+PluDJC_21480	*plu4344*	targeted	hypothetical protein
PluTT01m_07305	PluDJC_06920	*plu1415*	pseudo	hypothetical protein
PluTT01m_09465	PluDJC_09625	*plu1837*	pseudo	hypothetical protein
PluTT01m_15515	PluDJC_15415	*plu3017*	pseudo	hypothetical protein
PluTT01m_20440	PluDJC_19750	*plu3987*	pseudo	isopentenyl-diphosphate Delta-isomerase
PluTT01m_07670	PluDJC_07285	*plu1474*	pseudo	molybdenum-dependent transcriptional regulator
PluTT01m_11340	PluDJC_11700	*plu2198*	pseudo	phosphoribosylamine--glycine ligase
PluTT01m_22075	PluDJC_21390	*plu4320*	pseudo	restriction endonuclease subunit S
PluTT01m_22070	PluDJC_21365+PluDJC_21375	*plu4319*	targeted	type I restriction-modification system subunit M
	**genes with are disrupted only in *P. luminescens* strain TT01**			
**regular gene**	**disrupted gene**	**TT01 equivalent**	**type**	**protein name/function**
PluDJC_07045	PluTT01m_07430	*plu1438*	pseudo	(ilvE) branched-chain-amino-acid aminotransferase
PluDJC_20205	PluTT01m_20907+PluTT01m_20900	*plu4083*+*plu4081*	targeted	3-hydroxy-3-methylglutaryl-CoA lyase
PluDJC_21425	PluTT01m_22100	none	pseudo	AAA family ATPase
PluDJC_17095	PluTT01m_17245+PluTT01m_17235	*plu3354*+*plu3352*	targeted	ABC transporter ATP-binding protein
PluDJC_11815	PluTT01m_11467	none	pseudo	DUF3018 domain-containing protein
PluDJC_15080	PluTT01m_15180+PluTT01m_15170	*plu2955*+*plu2953*	targeted	DUF4431 domain-containing protein
PluDJC_04355	PluTT01m_04395+PluTT01m_04385	*plu0855*+*plu0853*	targeted	HlyD family secretion protein
PluDJC_09005	PluTT01m_09025+PluTT01m_09035	*plu1745*+*plu1747*	targeted	L,D-transpeptidase
PluDJC_06470	PluTT01m_06880+PluTT01m_06885	*plu1339*+*plu1340*	targeted	MARTX multifunctional-autoprocessing repeats-in-toxin holotoxin RtxA
PluDJC_16400	PluTT01m_16525+PluTT01m_16515	*plu3209*+*plu3207*	targeted	MARTX multifunctional-autoprocessing repeats-in-toxin holotoxin RtxA
PluDJC_11770	PluTT01m_11433	none	pseudo	RES domain-containing protein
PluDJC_11530	PluTT01m_11155	*plu2160*	pseudo	alpha/beta hydrolase
PluDJC_18410	PluTT01m_19120+PluTT01m_19110	*plu3729*+none	targeted	helix-turn-helix transcriptional regulator
PluDJC_11480	PluTT01m_11112	*plu2152*	pseudo	hypothetical protein

PluDJC_11490	PluTT01m_11115	none	pseudo	hypothetical protein
PluDJC_11775	PluTT01m_11437	none	pseudo	hypothetical protein
PluDJC_12115	PluTT01m_11770	*plu2281*	pseudo	hypothetical protein
PluDJC_12755	PluTT01m_12415+PluTT01m_12425	*plu2413+plu2415*	targeted	hypothetical protein
PluDJC_15835	PluTT01m_15940	none	pseudo	hypothetical protein
PluDJC_17105	PluTT01m_17265+PluTT01m_17255	*plu3358+plu3356*	targeted	hypothetical protein
PluDJC_17250	PluTT01m_17940	*plu3493+plu3494*	pseudo	hypothetical protein
PluDJC_20580	PluTT01m_21285+PluTT01m_21302	*plu4162+plu4166*	targeted	hypothetical protein
PluDJC_20785	PluTT01m_21515+PluTT01m_21505	*plu4209+plu4207*	targeted	hypothetical protein
PluDJC_22345	PluTT01m_23065	none	pseudo	hypothetical protein
PluDJC_23045	PluTT01m_23772+PluTT01m_23765	none+*plu4648*	targeted	hypothetical protein
PluDJC_08520	PluTT01m_08540+PluTT01m_08530	*plu1647+plu1645*	targeted	photopexin B
PluDJC_12360	PluTT01m_12020	*plu2331+plu2332*	pseudo	plasmid stabilization protein ParE
PluDJC_22365	PluTT01m_23085+PluTT01m_23095	*plu4517+plu4520*	targeted	sensor histidine kinase
PluDJC_09205	PluTT01m_09240	*plu1790*	pseudo	subtype I-F CRISPR-associated endonuclease Cas1
PluDJC_21460	PluTT01m_22160+PluTT01m_22170	*plu4338+plu4340*	targeted	type 2 isopentenyl-diphosphate Delta-isomerase
PluDJC_15065	PluTT01m_15155	*plu2949+plu2950*	pseudo	type II toxin-antitoxin system HicA family toxin
PluDJC_16650	PluTT01m_16795+PluTT01m_16775	*plu3259*+none	targeted	type VI secretion protein ImpK

Supplementary Table S6: Prophages as predicted by PhiSpy and Prophinder. A total of 21 prophage regions have been identified (Region). Regions are non-overlapping except for the longest prediction in R16 which overlaps with prophages in R15. Method terms consist of a letter indicating the method, a serial number (by method), and a genome indicator. Alternatively, the term "targeting" is assigned (see below). DJC refers to strain DJC, TT01m to the newly sequenced version of strain TT01 and TT01 to the published version of TT01. The letter S indicates a PhiSpy prediction, the letter A a prediction with Prophinder from the ACLAME server. PhiSpy predictions of the newly sequenced genomes were computed with the initial, non-curated PGAP annotation. For the published strain TT01, the Prophinder prediction was retrieved from the ACLAME server rather than being recomputed. For each prediction, the position in the corresponding genome and the predicted prophage length are indicated. For prophages from strain DJC and from the published version of the TT01 genome, the corresponding positions of the TT01m genome were identified to permit positional comparisons (column "relocated to TT01m"). Prophages in DJC inserts do not have an equivalent on the TT01m genome (indicated by "none"). Coordinates in parenthesis indicate that the prophage terminus could not be located on the TT01m genome as it is not located on a matchSEG. The methods term "targeting" refers to a manual assignment of prophage boundardies, which is based on analysis of splitting (or targeting) of protein-coding genes. Relevant details are mentioned in the comment column, commonly referenced to supplementary tables S1 and S2, including the serial number of that table.

Region	Method	relocated to TT01m	assigned to TT01m	length	assigned to TT01	length	assigned to DJC	length	comment
R01	S.01.TT01m	11631-29965	11631-29965	18335					TabS1#1 and TabS2#4: inversion
	A.01.TT01m	10251-28978	10251-28978	18728					
	S.01.TT01	1627-40946			1627-40947	39321			
	A.01.TT01	10251-30131			10251-29999	19881			
	S.01.DJC	11631-29792					11760-28906	17147	
	A.01.DJC	10251-29792					10380-30039	19660	
R02	S.02.TT01m	354332-398796	354332-398796	44465					

	S.02.TT01	345438-372676			345439-372677	27239			TabS2#11: 25 kb insert in TT01m
R03	S.03.TT01	985469-1029860			985470-1029861	44392			
R04	S.03.TT01m	1131700-1236267	1131700-1236267	104568					TabS2#46: 79 kb insert in TT01m
	S.04.TT01m	1256241-1344812	1256241-1344812	88572					
	S.04.TT01	1205149-1342771			1205150-1342771	137622			
	S.02.DJC	1194937-1238177					1187220-1230460	43241	
R05	S.05.TT01m	1553214-1629188	1553214-1629188	75975					TabS2#50+51: 11+12 kb inserts in TT01m
	S.05.TT01	1552827-1642788			1552827-1642788	89962			
R06	S.03.DJC	(1925016)-1933095					1805394-1868951	63558	TabS2#64: a 57 kb seq in DJC replaces a 5 kb seq in TT01m; within the 57 kb seq, 25 kb integrated into holin (PluTT01m_14920) at pos 79 (PluDJC_07980 + PluDJC_08180)
	A.02.DJC	none					1803738-1860591	56854	
	targeting	none					1811642-1837064	25423	
R07	S.06.TT01m	1983764-2058103	1983764-2058103	74340					
	A.02.TT01	1899374-2067669			1899373-2067668	168296			
	S.04.DJC	1983764-2058103					1918622-1991868	73247	
R08	S.05.DJC	2060560-(2134738)					1994325-2094935	100611	TabS2#77: 32 kb insert in DJC; 32 kb integrated into Cas3f (PluTT01m_09245) at pos 348/350 (PluDJC_09210 + PluDJC_09435)
	A.03.DJC	none					2068170-2100179	32010	
	targeting	-					2067895-2100517	32623	
R09	S.06.TT01	2182872-2197280			2182871-2197279	14409			
R10	S.07.TT01m	2361471-2406629	2361471-2406629	45159					TabS2#91: 34 kb insert in DJC
	S.06.DJC	2338244-2406629					2283028-2386921	103894	
	A.04.DJC	2372414-(2395527)					2317712-2375670	57959	
R11	S.07.TT01	2675668-2690815			2675668-2690815	15148			
R12	S.08.TT01m	2800483-2864064	2800483-2864064	63582					
	S.07.DJC	2800483-2864064					2765960-2828418	62459	
R13	S.09.TT01m	2973221-2980853	2973221-2980853	7633					
	S.08.TT01	2973651-2990529			2973651-2990529	16879			

	S.08.DJC	2973221-2980853					2927782-2935387	7606	
R14	S.10.TT01m	3409521-3483231	3409521-3483231	73711					TabS1#18: inversion; TabS2#141: 46 kb insert in TT01m; TabS2#147: a 3 kb seq in DJC replaces a 5 kb seq in TT01m; TabS2#148: inversion
	S.11.TT01m	3486008-3545456	3486008-3545456	59449					
	A.02.TT01m	3403657-3466572	3403657-3466572	62916					
	A.03.TT01m	3512862-3535872	3512862-3535872	23011					
	S.09.TT01	3410147-3482744			3410157-3482754	72598			
	S.10.TT01	3508312-3545456			3508362-3545466	37105			
	A.03.TT01	3403657-3456009			3403667-3456019	52353			
	A.04.TT01	3459809-3468139			3459819-3468149	8331			
	A.05.TT01	3508312-3537375			3508362-3537385	29064			
	S.09.DJC	3457221-3555249					3368371-3462728	94358	
	A.05.DJC	3523645-3537375					3431232-3444835	13604	
R15	A.06.TT01	3955218-3963518			3955228-3963528	8301			
	S.10.DJC	3941105-3963518					3844766-3868321	23556	
R16	S.12.TT01m	3954877-4099465	3954877-4099465	144589					TabS2#169: 78 kb insert in TT01m; 33 kb integrated into a probable restriction methylase at pos 179/180 (PluTT01m_17810 + PluTT01m_17570)
	A.04.TT01m	4007641-4076272	4007641-4076272	68632					
	S.11.TT01	3993859-4097445			3993869-4097455	103587			
	A.07.TT01	4007641-4076272			4007651-4076282	68632			
	A.08.TT01	4086051-4092697			4086061-4092707	6647			
	targeting	4035291-4068784		33494	-				
R17	S.11.DJC	4350893-4360656			.	.	4146494-4189653	43160	TabS2#178: 31 kb insert in DJC
R18	A.09.TT01	4367130-4421804			4368413-4423087	54675			
R19	S.12.TT01	4563989-4567477			4565272-4568760	3489			
R20	S.13.TT01	4721243-4744725			4722526-4746008	23483			
R21	S.12.DJC	(5559201)-(5565234)					5375312-5416648	41337	TabS2#223: 35 kb insert in DJC
	A.06.DJC	none					5384214-5405709	21496	

Supplementary Table S7: Copies of phage-related Repeat A (PhRepA) in the three *P. luminescens* strains. All copies of PhRepA are listed for the three analyzed genomes. The absence of a copy is indicated by dashes. Comments refer to the published version of strain TT01 (TT01), the new version of strain TT01 (TT01m) or strain DJC (DJC). For a description of the remnants B3 and D3 in strain DJC see the main text. Targeting by transposons (ISPlu9 or ISPlu20) is indicated.

	***P. luminescens* TT01**		***P. luminescens* TT01m**		***P. luminescens* DJC**		
	position	length	position	length	position	length	comment
A	598129-618518	20390	598128-618517	20390	571779-596010	24232	intergenic ISPlu20 and adhesion protein targeted by ISPlu20 (DJC)
B1	1304044-1313484	9441	1304043-1313483	9441	-	-	
B2	1313485-1321148	7664	1313484-1321147	7664	-	-	
B3	1321149-1336602	15454	1321148-1336602	15455	1239023-1246825	-	remnant in DJC
C	1620317-1638691	18375	1620317-1638691	18375	1506787-1527245	20459	primase targeted by ISPlu9 (TT01/ TT01m); ISPlu20 targets a hypothetical protein (DJC)
D1	4218342-4227209	8868	4218335-4227202	8868	-	-	intergenic ISPlu9 (TT01/ TT01m)
D2	-	-	4227203-4236070	8868	-	-	intergenic ISPlu9 (TT01m)
D3	4227210-4242480	15271	4236071-4251342	15272	4040907-4047311	6405	intergenic ISPlu9 (TT01/ TT01m); remnant in DJC
E1	4341138-4348505	7368	4350000-4357367	7368	4145601-4156591	10991	
E2	4348506-4357149	8644	-	-	4156592-4165227	8636	
E3	4357150-4366039	8890	4357368-4364756	7389	-	-	intergenic ISPlu9 (in TT01 but not in TT01m)
E4	4366040-4383140	17101	4364757-4381857	17101	-	-	intergenic ISPlu9 (TT01/ TT01m)
E5	-	-	-	-	4165228-4172583	7356	
E6	-	-	-	-	4172584-4179942	7359	
E7	-	-	-	-	4179943-4187655	7713	
E8	-	-	-	-	4187656-4203159	15504	

Supplementary Table S8: Genes and intergenic regions on PhRepA in the element copies. All genes and intergenic regions (as specified in the column "blocktype") occurring in copies of PhRepA are listed. These are the raw data from which Fig. 4b and 4c have been drawn. (a) for intergenic regions, there is commonly only one line for each strain which contains the distance between the adjacent genes in the corresponding column. Negative numbers indicate gene overlaps. The corresponding genes are commonly in the adjacent gene block but in few cases they are several blocks apart. The final intergenic block defines the distance to the start of the element. For some element copies, there is no upstream gene. In this case, the value represents the distance to the end of the element copy. In the few cases where a linetype 2 is used for an intergenic region, this specifies a transposon which has targetted this region. (b) For genes, the column "master_gene" lists the code of a protein which represents this gene. The column "description" lists the key domains (from InterPro) if assigned. The "adhesion" protein is referenced as such and not through the assigned domains. If no domain is assigned, the code under "master_gene" is repeated with the extension "-like". Genes are classified into a "subthread". At one end of the element are the "core" genes, on the other end of complete elements is the "adhesion" gene. There are five main threads (a: *plu0547*; b: *plu1129* plus *plu1130*; c: immunity_8 (imm8); d: VENN set 1; e: VENN set 2). Due to considerable heterogeneity, subthread b is subdivided into b1-b5. This subthread shows hybrid element formation as evident by comparing elements from *P. luminescens* strains TT01 and DJC. Threads and subthreads are indicated in Fig.4b and 4c. For each gene, there are multiple lines. Linetype 1 always refers to the protein code (ordered locus tag) of the protein from the corresponding strain. The term "notAnno" indicates that the corresponding ORF is not annotated in the TT01 genome; for the TT01m and DJC, notAnno cannot be assigned as we have post-predicted these ORFs if necessary. Linetype 2 always lists the subtype of the gene as reflected by an ORF code. Linetype 3 always lists the length of the gene in bp. For the adhesion protein, linetype 4 lists the length in codons, unless the gene is truncated. Linetype 5 indicates protein characteristics ("ok": a complete, probably functional protein; "Cfrag": most of the N-terminal region is truncated away, which is typical for non-terminal clustered element copies; "Nfrag": missing C-terminus due to truncation; "disrupted": the gene is nearly full-length but a few terminal residues are missing; "split_Plu20": the gene has been targeted by transposon ISPlu20; "DomainFused": the N-terminal region of the corresponding protein represents an adhesion factor that is fused to a C-terminal Plu1130-like domain. For other protein-coding genes, linetype 4 may indicate targetting by a transposon (ISPlu9, ISPlu20) or may point to gene disruption (Cfrag) of a secondary copy of a core gene.

blocktype	strain	linetype	subthread	copies of PhRepA																description	master_gene
				A	B1	B2	B3	C	D1	D2	D3	E1	E2	E3	E4	E5	E6	E7	E8		
gene	TT01	1	adhesion	*plu0548*	-	*plu1140*	*plu1149*	*plu1367*	-	-	*plu3594*	-	-	-	*plu3718*	-	-	-	-	adhesion	*plu0548*
		2		*plu0548*	-	*plu1149*	*plu1149*	*plu1367*	-	-	*plu3594*	-	-	-	*plu3718*	-	-	-	-		
		3		13749	-	1023	8814	8886	-	-	6408	-	-	-	9084	-	-	-	-		
		4		4582	-	-	2937	2961	-	-	2135	-	-	-	3027	-	-	-	-		
		5		ok	-	Cfrag	ok	ok	-	-	ok	-	-	-	DomainFused	-	-	-	-		
	TT01m	1		PluTT01m_02795	-	PluTT01m_05820	PluTT01m_05865	PluTT01m_07020	-	-	PluTT01m_18475	-	-	-	PluTT01m_19050	-	-	-	-		
		2		*plu0548*	-	-	*plu1149*	*plu1367*	-	-	*plu3594*	-	-	-	*plu3718*	-	-	-	-		
		3		13749	-	1038	8814	8886	-	-	6408	-	-	-	9084	-	-	-	-		
		4		4582	-	-	2937	2961	-	-	2135	-	-	-	3027	-	-	-	-		

		5		ok	-	Cfrag	ok	ok	-	-	ok	-	-		DomainFused	-	-	-	-		
	DJC	1		PluDJC_02725+ PluDJC_02705	-	-	PluDJC_05470	PluDJC_06630	-	-	PluDJC_17655	-	-	-	-	-	-	PluDJC_18325	PluDJC_18360		
		2		*plu0548*	-	-	-	*plu1367*	-	-	-	-	-	-	-	-	-	*plu1149*	*plu1149*		
		3		8475+5280	-	-	7803	8886	-	-	9057	-	-	-	-	-	-	642	8916		
		4		2825+1759	-	-	-	2961	-	-	3018	-	-	-	-	-	-	-	2971		
		5		split_Plu20	-	-	Nfrag	ok	-	-	gene fusion	-	-	-	-	-	-	Nfrag	ok		
intergenic	TT01	1	E	8	-	8	8	-11	-	-	16	-	-	14	14	-	-	-	-	-	
	TT01m	1		8	-	8	8	-11	-	-	16	-	-	14	14	-	-	-	-	-	
	DJC	1		-	-	-	-	-11	-	-	-	-	-	-	-	66	66	6	6	-	
gene	TT01	1		-	-	-	-	-	-	-	-	-	-	-	-	-	-	-	-	PAU_03360-like	PAU_03360
		2		-	-	-	-	-	-	-	-	-	-	-	-	-	-	-	-		
		3		-	-	-	-	-	-	-	-	-	-	-	-	-	-	-	-		
	TT01m	1		-	-	-	-	-	-	-	-	-	-	-	-	-	-	-	-		
		2		-	-	-	-	-	-	-	-	-	-	-	-	-	-	-	-		
		3		-	-	-	-	-	-	-	-	-	-	-	-	-	-	-	-		
	DJC	1		-	-	-	-	-	-	-	-	-	-	-	-	-	-	PluDJC_18320	PluDJC_18355		
		2		-	-	-	-	-	-	-	-	-	-	-	-	-	-	PAU_03360	PAU_03360		
		3		-	-	-	-	-	-	-	-	-	-	-	-	-	-	390	390		
gene	TT01	1	D	-	-	-	-	-	-	-	-	-	-	-	-	-	-	-	-	VENN	XSR1_930001
		2		-	-	-	-	-	-	-	-	-	-	-	-	-	-	-	-		
		3		-	-	-	-	-	-	-	-	-	-	-	-	-	-	-	-		
	TT01m	1		-	-	-	-	-	-	-	-	-	-	-	-	-	-	-	-		
		2		-	-	-	-	-	-	-	-	-	-	-	-	-	-	-	-		
		3		-	-	-	-	-	-	-	-	-	-	-	-	-	-	-	-		
	DJC	1		-	-	-	-	-	-	-	-	-	-	-	-	PluDJC_18245	PluDJC_18290	-	-		
		2		-	-	-	-	-	-	-	-	-	-	-	-	XSR1_930001	XSR1_930001	-	-		
		3		-	-	-	-	-	-	-	-	-	-	-	-	891	891	-	-		
intergenic	TT01	1		-	-	-	-	-	-	-	-	-	-	-	-	-	-	-	-	-	
	TT01m	1		-	-	-	-	-	-	-	-	-	-	-	-	-	-	-	-	-	
	DJC	1		-	-	-	-	-	-	-	-	-	-	-	-	12	12	-	-	-	
gene	TT01	1	D	-	-	-	-	-	-	-	-	-	-	-	-	-	-	-	-	XSR1_930002-like	XSR1_930002
		2		-	-	-	-	-	-	-	-	-	-	-	-	-	-	-	-		
		3		-	-	-	-	-	-	-	-	-	-	-	-	-	-	-	-		
	TT01m	1		-	-	-	-	-	-	-	-	-	-	-	-	-	-	-	-		
		2		-	-	-	-	-	-	-	-	-	-	-	-	-	-	-	-		
		3		-	-	-	-	-	-	-	-	-	-	-	-	-	-	-	-		
	DJC	1		-	-	-	-	-	-	-	-	-	-	-	-	PluDJC_18235	PluDJC_18280	-	-		

		2		-	-	-	-	-	-	-	-	-	-	-	-	XSR1_930002	XSR1_930002	-	-		
		3		-	-	-	-	-	-	-	-	-	-	-	-	258	258	-	-		
intergenic	TT01	1		-	-	-	-	-	-	-	-	-	21	-	-	-	-	-	-	-	
	TT01m	1		-	-	-	-	-	-	-	-	-	-	-	-	-	-	-	-	-	
	DJC	1		-	-	-	-	-	-	-	-	21	21	-	-	-	-	-	-	-	
gene	TT01	1	C	-	-	-	-	-	-	-	-	-	-	*plu3708*	*plu3717*	-	-	-	-	imm8	*plu3708*
		2		-	-	-	-	-	-	-	-	-	-	*plu3708*	*plu3708*	-	-	-	-		
		3		-	-	-	-	-	-	-	-	-	-	348	348	-	-	-	-		
	TT01m	1		-	-	-	-	-	-	-	-	-	-	PluTT01m_18995	PluTT01m_19045	-	-	-	-		
		2		-	-	-	-	-	-	-	-	-	-	*plu3708*	*plu3708*	-	-	-	-		
		3		-	-	-	-	-	-	-	-	-	-	348	348	-	-	-	-		
	DJC	1		-	-	-	-	-	-	-	-	-	-	-	-	-	-	-	-		
		2		-	-	-	-	-	-	-	-	-	-	-	-	-	-	-	-		
		3		-	-	-	-	-	-	-	-	-	-	-	-	-	-	-	-		
gene	TT01	1	B5	-	-	-	-	-	-	-	-	-	*plu3702*	-	-	-	-	-	-	*plu3702*-like	*plu3702*
		2		-	-	-	-	-	-	-	-	-	*plu3702*	-	-	-	-	-	-		
		3		-	-	-	-	-	-	-	-	-	954	-	-	-	-	-	-		
	TT01m	1		-	-	-	-	-	-	-	-	-	-	-	-	-	-	-	-		
		2		-	-	-	-	-	-	-	-	-	-	-	-	-	-	-	-		
		3		-	-	-	-	-	-	-	-	-	-	-	-	-	-	-	-		
	DJC	1		-	-	-	-	-	-	-	-	PluDJC_18160	PluDJC_18205	-	-	-	-	-	-		

		2		-	-	-	-	-	-	-	-	*plu3702*	*plu3702*	-	-	-	-	-	-		
		3		-	-	-	-	-	-	-	-	990	990	-	-	-	-	-	-		
intergenic	TT01	1		-	-	-	-	-	-	-	-	-	1	-	-	-	-	-	-	-	
	TT01m	1		-	-	-	-	-	-	-	-	-	-	-	-	-	-	-	-	-	
	DJC	1		-	-	-	-	-	-	-	-	1	1	-	-	-	-	-	-	-	
gene	TT01	1	B5	-	-	-	-	-	-	-	-	-	notAnno	-	-	-	-	-	-	XSR1_360056-like	XSR1_360056
		2		-	-	-	-	-	-	-	-	-	XSR1_360056	-	-	-	-	-	-		
		3		-	-	-	-	-	-	-	-	-	288	-	-	-	-	-	-		
	TT01m	1		-	-	-	-	-	-	-	-	-	-	-	-	-	-	-	-		
		2		-	-	-	-	-	-	-	-	-	-	-	-	-	-	-	-		
		3		-	-	-	-	-	-	-	-	-	-	-	-	-	-	-	-		
	DJC	1		-	-	-	-	-	-	-	-	PluDJC_18155	PluDJC_18200	-	-	-	-	-	-		
		2		-	-	-	-	-	-	-	-	XSR1_360056	XSR1_360056	-	-	-	-	-	-		
		3		-	-	-	-	-	-	-	-	288	288	-	-	-	-	-	-		
gene	TT01	1	B4	-	-	-	-	notAnno	-	-	-	-	-	-	-	-	-	-	-	AM629_21040-like	AM629_21040
		2		-	-	-	-	AM629_21040	-	-	-	-	-	-	-	-	-	-	-		
		3		-	-	-	-	633	-	-	-	-	-	-	-	-	-	-	-		
	TT01m	1		-	-	-	-	PluTT01m_07015	-	-	-	-	-	-	-	-	-	-	-		
		2		-	-	-	-	AM629_21040	-	-	-	-	-	-	-	-	-	-	-		
		3		-	-	-	-	633	-	-	-	-	-	-	-	-	-	-	-		
	DJC	1		-	-	-	-	PluDJC_06625	-	-	-	-	-	-	-	-	-	-	-		
		2		-	-	-	-	AM629_21040	-	-	-	-	-	-	-	-	-	-	-		
		3		-	-	-	-	639	-	-	-	-	-	-	-	-	-	-	-		
gene	TT01	1	B3	-	-	-	-	-	-	-	-	-	-	-	-	-	-	-	-	Colicin_D	Ppb6_03314
		2		-	-	-	-	-	-	-	-	-	-	-	-	-	-	-	-		
		3		-	-	-	-	-	-	-	-	-	-	-	-	-	-	-	-		
		1		-	-	-	-	-	-	-	-	-	-	-	-	-	-	-	-		

	TT01m	2		-	-	-	-	-	-	-	-	-	-	-	-	-	-	-	-		
		3		-	-	-	-	-	-	-	-	-	-	-	-	-	-	-	-		
	DJC	1		-	-	-	-	PluDJC_06615	-	-	-	-	-	-	-	-	-	-	-		
		2		-	-	-	-	Ppb6_03314	-	-	-	-	-	-	-	-	-	-	-		
		3		-	-	-	-	882	-	-	-	-	-	-	-	-	-	-	-		
intergenic	TT01	1		-	-	-	-	-	-	-	-	-	-	-	-	-	-	-	-	-	
	TT01m	1		-	-	-	-	-	-	-	-	-	-	-	-	-	-	-	-	-	
	DJC	1		-	-	-	-	-4	-	-	-	-	-	-	-	-	-	-	-	-	
gene	TT01	1	B3	-	-	-	-	-	-	-	-	-	-	-	-	-	-	-	-	Colicin_D_immunity	Ppb6_03315
		2		-	-	-	-	-	-	-	-	-	-	-	-	-	-	-	-		
		3		-	-	-	-	-	-	-	-	-	-	-	-	-	-	-	-		
	TT01m	1		-	-	-	-	-	-	-	-	-	-	-	-	-	-	-	-		
		2		-	-	-	-	-	-	-	-	-	-	-	-	-	-	-	-		
		3		-	-	-	-	-	-	-	-	-	-	-	-	-	-	-	-		
	DJC	1		-	-	-	-	PluDJC_06610	-	-	-	-	-	-	-	-	-	-	-		
		2		-	-	-	-	Ppb6_03315	-	-	-	-	-	-	-	-	-	-	-		
		3		-	-	-	-	276	-	-	-	-	-	-	-	-	-	-	-		
intergenic	TT01	1		-	-	-	-	-	-	-	-	-	-	-	-	-	-	-	-	-	
	TT01m	1		-	-	-	-	-	-	-	-	-	-	-	-	-	-	-	-	-	
	DJC	1		-	-	-	-	79	-	-	-	-	-	-	-	-	-	-	-	-	
gene	TT01	1	B3	-	-	-	-	-	-	-	-	-	-	-	-	-	-	-	-	Toxin_SymE	*plu0546*
		2		-	-	-	-	-	-	-	-	-	-	-	-	-	-	-	-		
		3		-	-	-	-	-	-	-	-	-	-	-	-	-	-	-	-		
	TT01m	1		-	-	-	-	-	-	-	-	-	-	-	-	-	-	-	-		
		2		-	-	-	-	-	-	-	-	-	-	-	-	-	-	-	-		
		3		-	-	-	-	-	-	-	-	-	-	-	-	-	-	-	-		
	DJC	1		-	-	-	-	PluDJC_06605	-	-	-	-	-	-	-	-	-	-	-		
		2		-	-	-	-	*plu0546*	-	-	-	-	-	-	-	-	-	-	-		
		3		-	-	-	-	111	-	-	-	-	-	-	-	-	-	-	-		
		4		-	-	-	-	Cfrag	-	-	-	-	-	-	-	-	-	-	-		
intergenic	TT01	1		-	-	-	-	-	-	-	-	-	-	-	-	-	-	-	-	-	
	TT01m	1		-	-	-	-	-	-	-	-	-	-	-	-	-	-	-	-	-	
	DJC	1		-	-	-	-	1	-	-	-	-	-	-	-	-	-	-	-	-	
gene	TT01	1	B3	-	-	-	-	-	-	-	-	-	-	-	-	-	-	-	-	*plu0542*-like	*plu0542*
		2		-	-	-	-	-	-	-	-	-	-	-	-	-	-	-	-		
		3		-	-	-	-	-	-	-	-	-	-	-	-	-	-	-	-		
	TT01m	1		-	-	-	-	-	-	-	-	-	-	-	-	-	-	-	-		
		2		-	-	-	-	-	-	-	-	-	-	-	-	-	-	-	-		
		3		-	-	-	-	-	-	-	-	-	-	-	-	-	-	-	-		

	DJC	1		-	-	-	-	PluDJC_06602	-	-	-	-	-	-	-	-	-	-	-		
		2		-	-	-	-	*plu0542*	-	-	-	-	-	-	-	-	-	-	-		
		3		-	-	-	-	111	-	-	-	-	-	-	-	-	-	-	-		
		4		-	-	-	-	Cfrag	-	-	-	-	-	-	-	-	-	-	-		
intergenic	TT01	1		-	33	-	-	-	-	-	-	-	-	-	-	-	-	-	-	-	
	TT01m	1		-	33	-	-	-	-	-	-	-	-	-	-	-	-	-	-	-	
	DJC	1		-	-	-	-	87	-	-	-	-	-	-	-	-	-	-	-	-	
gene	TT01	1	B2	-	*plu1133*	-	-	-	-	-	-	-	-	-	-	-	-	-	-	*plu1133-like*	*plu1133*
		2		-	*plu1133*	-	-	-	-	-	-	-	-	-	-	-	-	-	-		
		3		-	621	-	-	-	-	-	-	-	-	-	-	-	-	-	-		
	TT01m	1		-	PluTT01m_05780	-	-	-	-	-	-	-	-	-	-	-	-	-	-		
		2		-	*plu1133*	-	-	-	-	-	-	-	-	-	-	-	-	-	-		
		3		-	669	-	-	-	-	-	-	-	-	-	-	-	-	-	-		
	DJC	1		-	-	-	-	PluDJC_06600	-	-	-	-	-	-	-	-	-	-	-		
		2		-	-	-	-	*plu1133*	-	-	-	-	-	-	-	-	-	-	-		
		3		-	-	-	-	669	-	-	-	-	-	-	-	-	-	-	-		
intergenic	TT01	1		-	-11	-	-	-	-	-	-	-	-	-	-	-	-	-	-	-	
	TT01m	1		-	-11	-	-	-	-	-	-	-	-	-	-	-	-	-	-	-	
	DJC	1		-	-	-	-	-11	-	-	-	-	-	-	-	-	-	-	-	-	
gene	TT01	1	B2	-	*plu1132*	-	-	-	-	-	-	-	-	-	-	-	-	-	-	*plu1132-like*	*plu1132*
		2		-	*plu1132*	-	-	-	-	-	-	-	-	-	-	-	-	-	-		
		3		-	264	-	-	-	-	-	-	-	-	-	-	-	-	-	-		
	TT01m	1		-	PluTT01m_05775	-	-	-	-	-	-	-	-	-	-	-	-	-	-		
		2		-	*plu1132*	-	-	-	-	-	-	-	-	-	-	-	-	-	-		
		3		-	264	-	-	-	-	-	-	-	-	-	-	-	-	-	-		
	DJC	1		-	-	-	-	PluDJC_06595	-	-	-	-	-	-	-	-	-	-	-		
		2		-	-	-	-	*plu1132*	-	-	-	-	-	-	-	-	-	-	-		
		3		-	-	-	-	264	-	-	-	-	-	-	-	-	-	-	-		
intergenic	TT01	1		-	74	-	-	-	-	-	-	-	-	-	-	-	-	-	-	-	
	TT01m	1		-	74	-	-	-	-	-	-	-	-	-	-	-	-	-	-	-	
	DJC	1		-	-	-	-	74	-	-	-	-	-	-	-	-	-	-	-	-	
gene	TT01	1	B2	-	*plu1131*	-	-	-	-	-	-	-	-	-	-	-	-	-	-	*plu1131-like*	*plu1131*
		2		-	*plu1131*	-	-	-	-	-	-	-	-	-	-	-	-	-	-		
		3		-	621	-	-	-	-	-	-	-	-	-	-	-	-	-	-		
	TT01m	1		-	PluTT01m_05770	-	-	-	-	-	-	-	-	-	-	-	-	-	-		
		2		-	*plu1131*	-	-	-	-	-	-	-	-	-	-	-	-	-	-		
		3		-	621	-	-	-	-	-	-	-	-	-	-	-	-	-	-		
	DJC	1		-	-	-	-	PluDJC_06590	-	-	-	-	-	-	-	-	-	-	-		

		2		-	-	-	-	*plu1131*	-	-	-	-	-	-	-	-	-	-	-		
		3		-	-	-	-	621	-	-	-	-	-	-	-	-	-	-	-		
intergenic	TT01	1		-	4	-	-	-	-	-	-	-	-	-	-	-	-	-	-	-	
	TT01m	1		-	4	-	-	-	-	-	-	-	-	-	-	-	-	-	-	-	
	DJC	1		-	-	-	-	4	-	-	-	-	-	-	-	-	-	-	-	-	
gene	TT01	1		-	notAnno	-	-	-	-	-	-	-	-	-	-	-	-	-	-		
		2		-	Ppb6_03757	-	-	-	-	-	-	-	-	-	-	-	-	-	-		
		3		-	237	-	-	-	-	-	-	-	-	-	-	-	-	-	-		
	TT01m	1		-	PluTT01m_05765	-	-	-	-	-	-	-	-	-	-	-	-	-	-		
		2	B2	-	Ppb6_03757	-	-	-	-	-	-	-	-	-	-	-	-	-	-	Ppb6_03757-like	Ppb6_03757
		3		-	237	-	-	-	-	-	-	-	-	-	-	-	-	-	-		
	DJC	1		-	-	-	-	PluDJC_06585	-	-	-	-	-	-	-	-	-	-	-		
		2		-	-	-	-	Ppb6_03757	-	-	-	-	-	-	-	-	-	-	-		
		3		-	-	-	-	237	-	-	-	-	-	-	-	-	-	-	-		
intergenic	TT01	1		-	259	-	-	16	21	-	-	21	33	-	-	-	-	-	-	-	
	TT01m	1		-	259	-	-	16	21	21	-	21	-	-	-	-	-	-	-	-	
	DJC	1		-	-	-	-	259	-	-	-	33	-	-	-	-	-	-	-	-	
gene	TT01	1		-	*plu1130*	-	-	*plu1366*	*plu3584*	-	*plu3593*	*plu3693*	*plu3701*	*plu3709*	-	-	-	-	-		
		2		-	*plu1130*	-	-	*plu1130*	*plu3584*	-	*plu3584*	*plu1130*	*plu1130*	*plu3718*	-	-	-	-	-		
		3		-	876	-	-	876	860	-	860	876	876	873	-	-	-	-	-		
	TT01m	1		-	PluTT01m_05755	-	-	PluTT01m_07010	PluTT01m_18390	PluTT01m_18430	PluTT01m_18470	PluTT01m_18960	-	PluTT01m_19000	-	-	-	-	-		
		2	B1	-	*plu1130*	-	-	*plu1130*	*plu3584*	*plu3584*	*plu3584*	*plu1130*	-	*plu3718*	-	-	-	-	-	*plu1130*-like	*plu1130*
		3		-	912	-	-	909	900	900	891	912	-	888	-	-	-	-	-		
	DJC	1		-	-	-	-	PluDJC_06580	-	-	-	PluDJC_18150	PluDJC_18195	-	-	-	-	-	-		
		2		-	-	-	-	*plu1130*	-	-	-	*plu1130*	*plu1130*	-	-	-	-	-	-		
		3		-	-	-	-	876	-	-	-	876	876	-	-	-	-	-	-		
intergenic	TT01	1		-	-4	-	-	-4	-	-	-	-4	-4	-	-	-	-	-	-	-	
	TT01m	1		-	-4	-	-	-4	-	-	-	-4	-	-	-	-	-	-	-	-	
	DJC	1		-	-	-	-	-4	-	-	-	-4	-4	-	-	-	-	-	-	-	
gene	TT01	1		-	*plu1129*	-	-	*plu1365*	*plu3583*	-	*plu3592*	*plu3692*	*plu3700*	-	-	-	-	-	-		
		2		-	*plu1129*	-	-	*plu1129*	*plu3583*	-	*plu3583*	*plu1129*	*plu1129*	-	-	-	-	-	-		
		3		-	318	-	-	318	312	-	312	318	318	-	-	-	-	-	-		
	TT01m	1		-	PluTT01m_05750	-	-	PluTT01m_07005	PluTT01m_18385	PluTT01m_18425	PluTT01m_18465	PluTT01m_18955	-	-	-	-	-	-	-		
		2	B1	-	*plu1129*	-	-	*plu1129*	*plu3583*	*plu3583*	*plu3583*	*plu1129*	-	-	-	-	-	-	-	*plu1129*-like	*plu1129*
		3		-	318	-	-	318	312	312	312	318	-	-	-	-	-	-	-		
	DJC	1		-	-	-	-	PluDJC_06575	-	-	-	PluDJC_18145	PluDJC_18190	-	-	-	-	-	-		
		2		-	-	-	-	*plu1129*	-	-	-	*plu1129*	*plu1129*	-	-	-	-	-	-		

		3		-	-	-	-	318	-	-	-	318	318	-	-	-	-	-	-		
gene	TT01	1	A	*plu0547*	-	*plu1139*	*plu1148*	-	-	-	-	-	-	-	-	-	-	-	-	*plu0547*-like	*plu0547*
		2		*plu0547*	-	*plu0547*	*plu0547*	-	-	-	-	-	-	-	-	-	-	-	-		
		3		420	-	420	420	-	-	-	-	-	-	-	-	-	-	-	-		
	TT01m	1		PluTT01m_02790	-	PluTT01m_05815	PluTT01m_05855	-	-	-	-	-	-	-	-	-	-	-	-		
		2		*plu0547*	-	*plu0547*	*plu0547*	-	-	-	-	-	-	-	-	-	-	-	-		
		3		420	-	420	420	-	-	-	-	-	-	-	-	-	-	-	-		
	DJC	1		PluDJC_02700	-	-	-	-	-	-	-	-	-	-	-	-	-	-	-		
		2		*plu0547*	-	-	-	-	-	-	-	-	-	-	-	-	-	-	-		
		3		420	-	-	-	-	-	-	-	-	-	-	-	-	-	-	-		
intergenic	TT01	1		45	44	45	45	44	42	-	42	44	44	52	52	-	-	-	-	-	
	TT01m	1		45	44	45	45	44	42	42	42	44	-	52	52	-	-	-	-	-	
	DJC	1		45	-	-	-	44	-	-	-	44	44	-	-	47	47	78	78	-	
gene	TT01	1	core	*plu0546*	*plu1128*	*plu1138*	*plu1147*	*plu1364*	*plu3582*	-	*plu3591*	*plu3691*	*plu3699*	*plu3707*	*plu3716*	-	-	-	-	Toxin_SymE	*plu0546*
		2		*plu0546*	*plu1128*	*plu0546*	*plu0546*	*plu1128*	*plu0546*	-	*plu0546*	*plu1128*	*plu1128*	*plu3707*	*plu3707*	-	-	-	-		
		3		243	243	243	243	243	243	-	243	243	243	228	228	-	-	-	-		
	TT01m	1		PluTT01m_02785	PluTT01m_05745	PluTT01m_05810	PluTT01m_05850	PluTT01m_07000	PluTT01m_18380	PluTT01m_18420	PluTT01m_18460	PluTT01m_18950	-	PluTT01m_18990	PluTT01m_19040	-	-	-	-		
		2		*plu0546*	*plu1128*	*plu0546*	*plu0546*	*plu1128*	*plu0546*	*plu0546*	*plu0546*	*plu1128*	-	*plu3707*	*plu3707*	-	-	-	-		
		3		243	243	243	243	243	243	243	243	243	-	228	228	-	-	-	-		
	DJC	1		PluDJC_02695	-	-	-	PluDJC_06570	-	-	-	PluDJC_18140	PluDJC_18185	-	-	PluDJC_18230	PluDJC_18275	PluDJC_18315	PluDJC_18350		
		2		*plu0546*	-	-	-	*plu1128*	-	-	-	*plu1128*	*plu1128*	-	-	*plu0546*	*plu0546*	*plu1128*	*plu1128*		
		3		243	-	-	-	243	-	-	-	243	243	-	-	243	243	243	243		
intergenic	TT01	1		98	92	98	98	92	102	-	102	92	92	72	72	-	-	-	-	-	
	TT01m	1		98	92	98	98	92	102	102	102	92	-	72	72	-	-	-	-	-	
	DJC	1		98	-	-	-	92	-	-	-	16+84	92	-	-	98	98	74	74	-	
		2		-	-	-	-	-	-	-	-	Plu20	-	-	-	-	-	-	-	-	
gene	TT01	1	core	*plu0545*	*plu1127*	*plu1137*	*plu1146*	*plu1363*	*plu3581*	-	*plu3590*	*plu3690*	*plu3698*	*plu3706*	*plu3715*	-	-	-	-	Cro/C1-type_HTH	*plu0545*
		2		*plu0545*	*plu1127*	*plu0545*	*plu0545*	*plu1127*	*plu3581*	-	*plu3581*	*plu1127*	*plu1127*	*plu3706*	*plu3706*	-	-	-	-		
		3		336	339	336	336	339	387	-	387	339	339	363	363	-	-	-	-		
	TT01m	1		PluTT01m_02780	PluTT01m_05740	PluTT01m_05805	PluTT01m_05845	PluTT01m_06995	PluTT01m_18375	PluTT01m_18415	PluTT01m_18455	PluTT01m_18945	-	PluTT01m_18985	PluTT01m_19035	-	-	-	-		
		2		*plu0545*	*plu1127*	*plu0545*	*plu0545*	*plu1127*	*plu3581*	*plu3581*	*plu3581*	*plu1127*	-	*plu3706*	*plu3706*	-	-	-	-		
		3		372	339	339	339	339	387	387	387	339	-	363	363	-	-	-	-		
	DJC	1		PluDJC_02690	-	-	-	PluDJC_06565	-	-	-	PluDJC_18120	PluDJC_18180	-	-	PluDJC_18225	PluDJC_18270	PluDJC_18310	PluDJC_18345		
		2		*plu0545*	-	-	-	*plu1127*	-	-	-	*plu1127*	*plu1127*	-	-	*plu0545*	*plu0545*	PTE_00007	PTE_00007		
		3		372	-	-	-	339	-	-	-	339	339	-	-	339	339	399	399		
int	TT01	1		187	132	187	187	132	101	-	101	132	132	132	132	-	-	-	-	-	

	TT01m	1		187	132	187	187	132	101	101	101	132	-	132	132	-	-	-	-	-	
	DJC	1		187	-	-	-	132	-	-	-	132	132	-	-	187	187	99	99	-	
gene	TT01	1	core	*plu0544*	*plu1126*	*plu1136*	*notAnno*	*notAnno*	*plu3580*	-	*plu3589*	*plu3689*	*plu3697*	*plu3705*	*plu3714*	-	-	-	-	DNA_primase	*plu0544*
		2		*plu0544*	*plu0544*	*plu0544*	*plu0544*	*plu0544*	*plu0544*	-	*plu0544*	*plu0544*	*plu0544*	*plu0544*	*plu0544*	-	-	-	-		
		3		3315	3315	3315	3314	2986+341	3315	-	3315	3315	3315	3315	3318	-	-	-	-		
		4		-	-	-	-	Plu9	-	-	-	-	-	-	-	-	-	-	-		
	TT01m	1		PluTT01m_02775	PluTT01m_05735	PluTT01m_05800	PluTT01m_05840	PluTT01m_06980+ PluTT01m_06990	PluTT01m_18370	PluTT01m_18410	PluTT01m_18450	PluTT01m_18940	-	PluTT01m_18980	PluTT01m_19030	-	-	-	-		
		2		*plu0544*	*plu0544*	*plu0544*	*plu0544*	*plu0544*	*plu0544*	*plu0544*	*plu0544*	*plu0544*	-	*plu0544*	*plu0544*	-	-	-	-		
		3		3315	3315	3315	3315	2986+341	3315	3315	3316	3315	-	3315	3318	-	-	-	-		
		4		-	-	-	-	Plu9	-	-	-	-	-	-	-	-	-	-	-		
	DJC	1		PluDJC_02685	-	-	-	PluDJC_06560	-	-	-	PluDJC_18115	PluDJC_18175	-	-	PluDJC_18220	PluDJC_18265	PluDJC_18305	PluDJC_18340		
		2		*plu0544*	-	-	-	*plu0544*	-	-	-	*plu0544*	*plu0544*	-	-	plu0544	plu0544	plu0544	plu0544		
		3		3315	-	-	-	3318	-	-	-	3315	3315	-	-	3315	3315	3315	3315		
intergenic	TT01	1		-14	-14	-14	-14	-14	-14	-	-14	-14	-14	-14	-14	-	-	-	-	-	
	TT01m	1		-14	-14	-14	-14	-14	-14	-14	-14	-14	-	-14	-14	-	-	-	-	-	
	DJC	1		-14	-	-	-	-14	-	-	-	-14	-14	-	-	-14	-14	-14	-14	-	
gene	TT01	1	core	*plu0543*	*plu1125*	*plu1135*	*plu1143*	*plu1359*	*plu3579*	-	*plu3588*	*plu3688*	*plu3696*	*plu3704*	*plu3713*	-	-	-	-	integrase	*plu0543*
		2		*plu0543*	*plu0543*	*plu0543*	*plu0543*	*plu0543*	*plu0543*	-	*plu0543*	*plu0543*	*plu0543*	*plu0543*	*plu0543*	-	-	-	-		
		3		1131	1113	1131	1131	1131	1131	-	1131	1113	1131	1131	1131	-	-	-	-		
	TT01m	1		PluTT01m_02770	PluTT01m_05730	PluTT01m_05795	PluTT01m_05835	PluTT01m_06975	PluTT01m_18365	PluTT01m_18405	PluTT01m_18445	PluTT01m_18935	-	PluTT01m_18975	PluTT01m_19025	-	-	-	-		
		2		*plu0543*	*plu0543*	*plu0543*	*plu0543*	*plu0543*	*plu0543*	*plu0543*	*plu0543*	*plu0543*	-	*plu0543*	*plu0543*	-	-	-	-		
		3		1131	1113	1131	1131	1131	1131	1131	1131	1113	-	1131	1131	-	-	-	-		
	DJC	1		PluDJC_02680	-	-	-	PluDJC_06555	-	-	-	PluDJC_18110	PluDJC_18170	-	-	PluDJC_18215	PluDJC_18260	PluDJC_18300	PluDJC_18335		
		2		*plu0543*	-	-	-	*plu0543*	-	-	-	*plu0543*	*plu0543*	-	-	*plu0543*	*plu0543*	*plu0543*	*plu0543*		
		3		1131	-	-	-	1131	-	-	-	1113	1113	-	-	1131	1131	1113	1113		
intergenic	TT01	1		230	248	230	230	230	231	231	231	248	248	230	230	-	-	-	-	-	
		2																			
	TT01m	1		230	248	230	230	230	231	231	231	248	-	230	230	-	-	-	-	-	
		2																			
	DJC	1		230	-	-	-	230	-	-	-	248	248	-	-	230	230	248	248	-	
		2																			
gene	TT01	1		notAnno	notAnno	notAnno	*plu1142*	notAnno	notAnno	-	notAnno	notAnno	notAnno	notAnno	notAnno					plu1142-like	*plu1142*
		2		*plu1142*	*plu1142*	*plu1142*	*plu1142*	*plu1142*	*plu1142*	-	*plu1142*	*plu1142*	*plu1142*	*plu1142*	*plu1142*						
		3																			
	TT01m	1		PluTT01m_02765	PluTT01m_05727	PluTT01m_05790	PluTT01m_05830	PluTT01m_06970	PluTT01m_18362	PluTT01m_18402	PluTT01m_18442	PluTT01m_18930	-	PluTT01m_18970	PluTT01m_19017						
		2		*plu1142*	*plu1142*	*plu1142*	*plu1142*	*plu1142*	*plu1142*	*plu1142*	*plu1142*	*plu1142*		*plu1142*	*plu1142*						
		3		273	273	273	273	276	273	273	273	276		276	273						
	DJC	1		PluDJC_02672+ PluDJC_02677	-	-	-	PluDJC_06550	-	-	-	PluDJC_18105	PluDJC_18167	-	-	PluDJC_18212	PluDJC_18257	PluDJC_18297	PluDJC_18332		

		2		*plu1142*				*plu1142*				*plu1142*	*plu1142*			*plu1142*	*plu1142*	*plu1142*	*plu1142*		
		3		72+210				276				276	273			273	279	273	273		
		4		Plu9																	
intergenic	TT01	1		56	51	56	56	56	18+42	18+42	18+42	56	56	56	19+47	-	-	-	-	-	
		2		-	-	-	-	-	Plu9	-	Plu9	-	-	Plu9	Plu9	-	-	-	-	-	
	TT01m	1		56	51	56	56	56	18+42	18+42	18+42	56	-	56	19+47	-	-	-	-	-	
		2		-	-	-	-	-	Plu9	Plu9	Plu9	-	-	-	Plu9	-	-	-	-	-	
	DJC	1		51	-	-	-	56	-	-	-	56	51	-	-	51	48	51	51	-	
		2			-	-	-	-	-	-	-	-	-	-	-	-	-	-	-	-	
gene	TT01	1	core	*plu0542*	*plu1124*	*plu1134*	*plu1141*	*plu1357*	notAnno	-	*plu3586*	notAnno	notAnno	notAnno	*plu3711*	-	-	-	-	*plu0542-like*	*plu0542*
		2		*plu0542*	*plu0542*	*plu0542*	*plu0542*	*plu0542*	*plu0542*	-	*plu0542*	*plu0542*	*plu0542*	*plu0542*	*plu0542*	-	-	-	-		
		3		267	267	267	267	267	267	-	267	267	267	267	267	-	-	-	-		
	TT01m	1		PluTT01m_02757	PluTT01m_05725	PluTT01m_05782	PluTT01m_05822	PluTT01m_06965	PluTT01m_18355	PluTT01m_18395	PluTT01m_18435	PluTT01m_18925	-	PluTT01m_18965	PluTT01m_19010	-	-	-	-		
		2		*plu0542*	*plu0542*	*plu0542*	*plu0542*	*plu0542*	*plu0542*	*plu0542*	*plu0542*	*plu0542*	-	*plu0542*	*plu0542*	-	-	-	-		
		3		261	261	261	261	261	261	261	261	261	-	261	261	-	-	-	-		
	DJC	1		PluDJC_02670	-	-	-	PluDJC_06545	-	-	-	PluDJC_18100	PluDJC_18165	-	-	PluDJC_18210	PluDJC_18250	PluDJC_18295	PluDJC_18330		
		2		*plu0542*	-	-	-	*plu0542*	-	-	-	*plu0542*	*plu0542*	-	-	*plu0542*	*plu0542*	*plu0542*	*plu0542*		
		3		261	-	-	-	261	-	-	-	261	261	-	-	261	261	261	261		
intergenic	TT01	1		54	54	54	54	54	54	-	54	54	54	54	54	-	-	-	-	-	
	TT01m	1		54	54	54	54	54	54	54	54	54	-	54	54	-	-	-	-	-	
	DJC	1		54	-	-	-	54	-	-	-	54	54	-	-	54	54	54	54	-	

Supplementary Table S9: Oligonucleotides used for *P. luminescens* strain diagnostics to differentiate between strain TT01 and DJC.

Primer name	Sequence 5' → 3'
candidate1_fwd	TTCTGTAGTATTACTTCGCAGTTAAAG
candidate1_rev	TTGTTATTAATGCTGACGTTCCTATTTATG
candidate2_fwd	ATATCTTGGGATGGGTCTCTTTAGGTTTTG
candidate2_rev	GGTAAATGCAAAATATTCACCTACTG
candidate3_fwd	TTCTTAGAAACTGTTAACGTTCCTATTG
candidate3_rev	ATGGCTACTAATACAGAACGTAAAGAGAC
candidate4_fwd	CAGGAGCTACGCGACAAGACCTTGC
candidate4_rev	GCGATCGTAAACTTCTATTTG
candidate5_fwd	TGTCGATGACCGAATTCAGTGAATG
candidate5_rev	CGTGCCACCTGACTCAATTGTTG

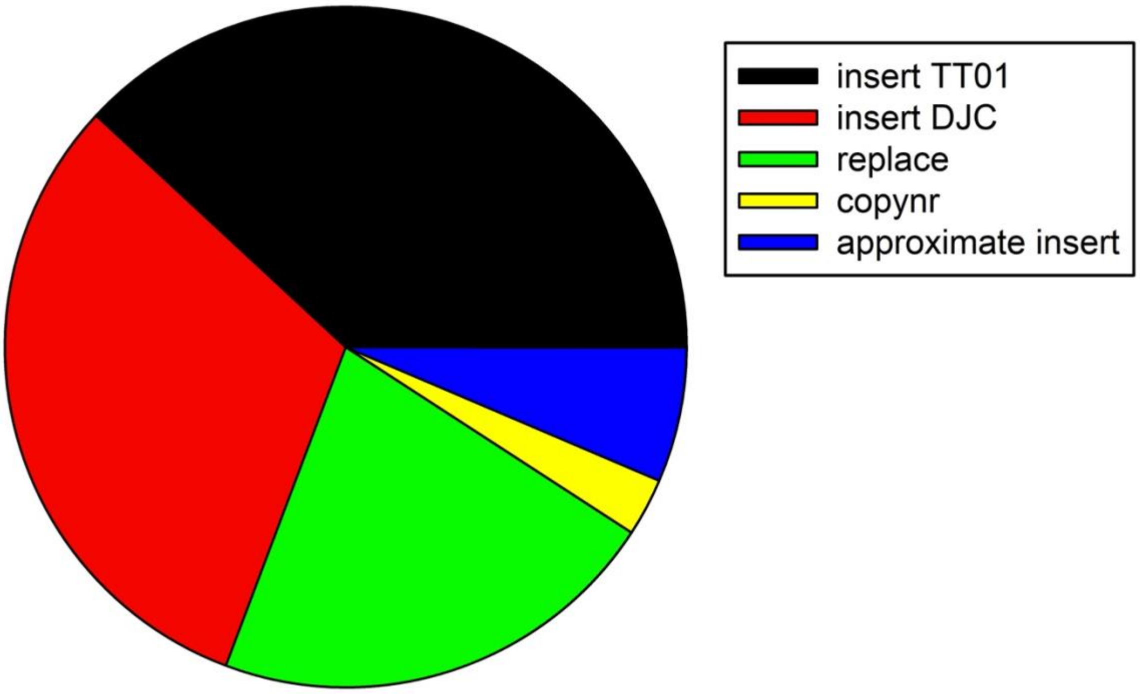

Figure S1: Relative contribution of divergence region classes between *P. luminescens* TT01 and DJC. For indels and approximate inserts, strain-specific data are shown. The few approximate inserts (13 total, 8 in DJC, 5 in TT01m) are not graphically separated. All other classes are symmetric between the two strains.

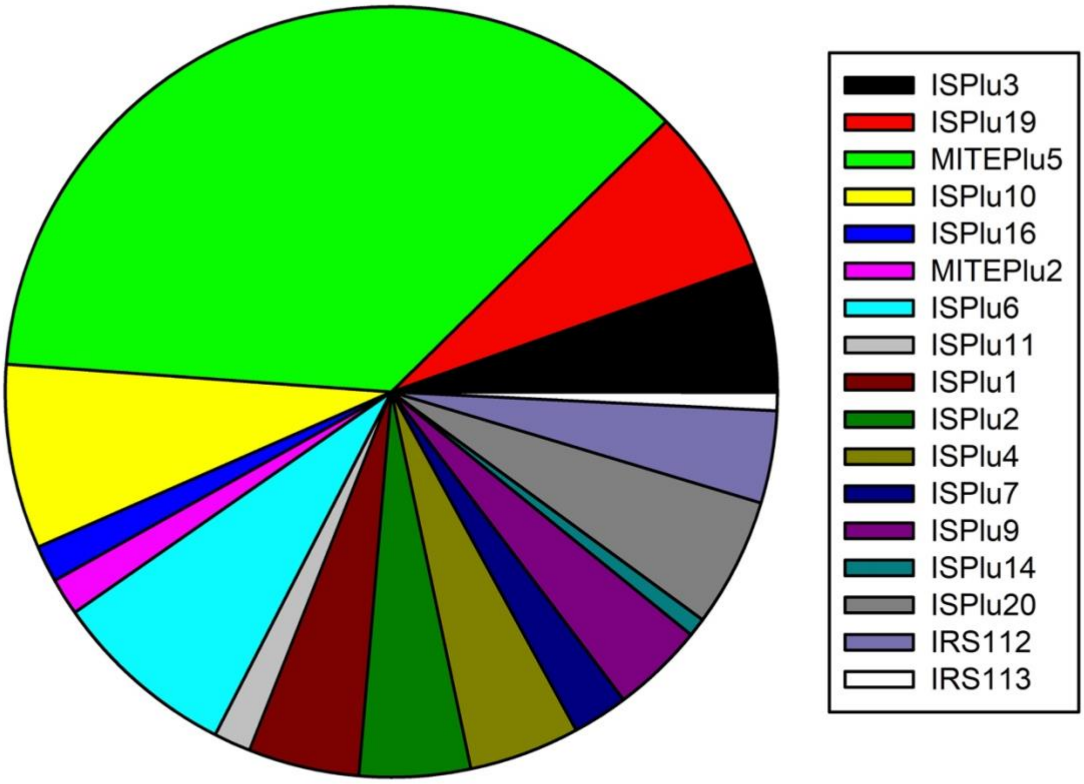

Figure S2: Relative frequency of transposons and other mobile genetic elements in indels and approximate inserts. ISPlu3, ISPlu19 and MITEPlu5 correspond to the CCC subtype of IS630-type elements (63 total of which 47 are MITEPlu5), ISPlu10+ISPlu16+MITEPlu2 correspond to the AATAA subtype of IS630-type elements (14 total), and ISPlu6 and ISPlu11 are both IS982-type elements (12 total). All other elements are unrelated to each other.
